# Photoswitchable COX-2-Selective Inhibitors as Light-Regulated Anti-Inflammatory Agents

**DOI:** 10.1021/jacs.6c03529

**Published:** 2026-04-28

**Authors:** Amanda Morales, Alejandro Cruz, Álex Pérez-Sánchez, Danilo D’Avino, Gaia Galassi, Erica Ginevra Milano, Irene Bernareggi, Carla Arenós-Bach, Giovanni Grazioso, Ramon Alibés, Jordi Hernando, Pau Gorostiza, Antonietta Rossi, Anna Pistocchi, Carlo Matera, Félix Busqué, Àngels González-Lafont, José M. Lluch

**Affiliations:** † Departament de Química, 16719 Universitat Autònoma de Barcelona, Bellaterra (Barcelona) 08193, Spain; ‡ Departament d’Enginyeria Química (EQ), ETSEIB, 5116 Universitat Politècnica de Catalunya - BarcelonaTech (UPC), Campus Sud, Edif. PG, Av. Diagonal, 647, Barcelona 08028, Spain; § Department of Pharmacy, School of Medicine and Surgery, University of Naples Federico II, Naples 80131, Italy; ∥ Department of Medical Biotechnology and Translational Medicine, L.I.T.A., 9304 University of Milan, Via Fratelli Cervi 93, Segrate, Milano 20054, Italy; ⊥ 9304 Catalan Institution for Research and Advanced Studies (ICREA), Barcelona 08010, Spain; # Institute for Bioengineering of Catalonia (IBEC), 284118 The Barcelona Institute for Science and Technology, Barcelona 08028, Spain; ∇ Biomedical Research Networking Center in Bioengineering, Biomaterials, and Nanomedicine (CIBER-BBN), Madrid 28029, Spain; ○ Department of Pharmaceutical Sciences, University of Milan, Milan 20133, Italy

## Abstract

Despite their extensive use, the therapeutic potential of nonsteroidal anti-inflammatory drugs (NSAIDs) remains significantly constrained by adverse side effects. This limitation primarily arises from insufficient selectivity, as current NSAIDs inhibit not only the inducible cyclooxygenase-2 (COX-2) isoform at sites of inflammation but also constitutive COX-2 in healthy tissues and, frequently, cyclooxygenase-1. To address this challenge, we developed photoswitchable NSAIDs that combine COX-2 selectivity with light-controlled activity to enable spatiotemporal confinement of the therapeutic effect at inflamed tissues. Following computational design and screening of a library of azoaromatic derivatives of celecoxib—the most widely used COX-2 selective NSAID—three photoswitchable analogues were synthesized, which exhibited reversible and efficient *trans*-*cis* photoconversion. Light-controlled and selective COX-2 inhibition was demonstrated for these compounds *in vitro*, reaching up to 5-fold potency enhancement in macrophage assays upon photoisomerization from the initial, dark-adapted *trans* isomer to the *cis* state. The best candidate displayed *in vivo* efficacy in a zebrafish model of acute inflammation, where administration of the photoinduced *cis* form reduced leukocyte recruitment at the wound site. These findings position photoswitchable NSAIDs as a promising alternative to conventional drugs for treating inflammation and related conditions, including cancer.

## Graphical abstract

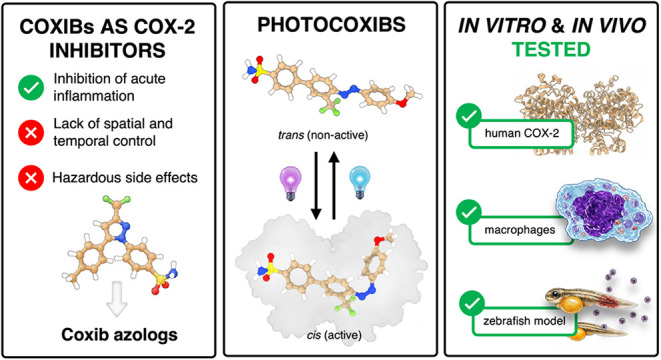

## Introduction

Inflammation is a fundamental protective mechanism of the human body, initiated by the immune system to eliminate pathogens, restore homeostasis, and repair damaged tissue.^1–3^ However, failure to activate resolution pathways perpetuates the inflammatory response, ultimately leading to chronic inflammation—a long-term immune reaction causing numerous harmful effects. In fact, chronic inflammatory states are recognized as major contributors to global mortality, accounting for more than 50% of deaths associated with inflammation-related pathologies.^2,3^ Consequently, anti-inflammatory drugs have become an essential pillar of modern medicine.^4^

Commonly prescribed anti-inflammatory drugs primarily act on the initial (acute) phase of inflammation, which is triggered by key pro-inflammatory enzymes that convert arachidonic acid (AA) into lipid mediators such as prostaglandins (PG).^5–7^ Among these enzymes, cyclooxygenase (COX)-2, which is transiently expressed at sites of inflammation, plays a pivotal role and is therefore the principal target of widely used nonsteroidal anti-inflammatory drugs (NSAIDs).^8^ However, NSAIDs exhibit limited pharmacological selectivity, often causing severe side effects and contributing to immunosuppression.^9^ Classical NSAIDs, such as aspirin and ibuprofen, also inhibit the enzyme COX-1, an isoform that is constitutively expressed in most tissues to regulate essential physiological processes.^10^ For instance, this is the case with gastric mucosa protection, which is detrimentally affected by NSAID activity on COX-1, eventually leading to gastric ulceration and internal bleeding in a significant number of users.^11^

To mitigate these issues, COX-2-selective NSAIDs—known as coxibs—were developed, including celecoxib (CEL) and rofecoxib.^12,13^ Unfortunately, these drugs also inhibit constitutive COX-2 enzymes that, although less abundant than COX-1, are expressed in healthy tissues to maintain homeostasis—e.g., in the brain, thymus, gut, and kidney.^14^ As a result, coxibs can also cause harmful side effects, such as an increased risk of developing cardiovascular events like strokes, which are typically associated with the unbalanced inhibition of constitutive COX-1 and COX-2.^15,16^ For this reason, some coxibs—e.g., rofecoxib—were withdrawn from the market.^17^ In fact, these cardiovascular side effects are associated with all NSAIDs except aspirin and increase personal risk of having a heart attack or stroke by as much as 30% after only 2 weeks of regular use.^18^

Inflammation is not only linked to common inflammatory diseases but is also recognized as a hallmark of cancer,^19,20^ paving the way for innovative therapeutic strategies in cancer immunotherapy.^21^ Prostaglandins E_2_ (PGE_2_) produced by COX-2 induction in most cancer cells promote tumor growth, proliferation, and metastasis,^22–24^ leading to a poor response of immunotherapy.^25–27^ COX-2 inhibition has been proposed to counteract PGE_2_-mediated immunosuppression and also enhance sensitivity to radio- and chemotherapy. However, the significant side effects of NSAIDs^18,28^ have hampered drug development, limiting the therapeutic potential of COX-2 blockade despite epidemiologic evidence that long-term NSAID use—particularly CEL—reduces cancer incidence and delays progression.^19,20,25,29^ In addition to this challenge, COX-2 expression in cancer tissue is up to 100 times higher than in normal inflammation,^30,31^ necessitating much larger NSAID doses for effective cancer treatment, which would dramatically increase toxicity and render current NSAIDs unsuitable for this purpose. Accordingly, the use of CEL is currently not recommended for cancer treatment.

In light of these limitations, fully harnessing the therapeutic potential of NSAIDs to treat chronic inflammation and other pathologies, such as cancer, requires precise regulation of their inhibitory activity in both space and time; i.e., they should combine COX-2 selectivity with the capacity to engage only the transiently expressed enzyme at inflammatory loci, minimizing unwanted activity in healthy tissues. In this work, we propose to reach this goal through photopharmacology, an emerging field that pursues the optical control of drug activity with spatiotemporal precision.^32–34^ To validate this concept, we pioneered the development of photoswitchable coxib-inspired derivatives that reversibly toggle between two states with distinct COX-2 inhibitory activity under irradiation. To this aim, as a starting point for the design of these photoswitches, we have chosen the CEL structure due to its well-known effectiveness as an anti-inflammatory drug. Through a combined *in* *silico*, synthetic, *in vitro,* and *in vivo* approach, we present evidence that these compounds allow reducing the degree of inflammation in a light-controlled manner, laying the groundwork for the future design of next-generation anti-inflammatory therapies with reduced adverse side effects and possible applications to cancer treatment.

## Results and Discussion

### Computational Design of Photoswitchable COX-2 Inhibitors

The design of photoswitchable coxibs was inspired by the structure of CEL, one of the most popular COX-2-selective NSAIDs. As shown in Figure 1, CEL presents a bent structure consisting of three different aromatic rings: a central trifluoromethylated pyrazole core and two lateral phenyl units bearing sulfonamide and methyl substituents, which enable the compound to selectively inhibit COX-2 without significantly affecting COX-1. Two features are considered critical for the selective binding of CEL (and other coxibs) to COX-2 (Figure 2): (1) the substitution of Ile523 in COX-1 by the smaller residue Val523 in COX-2, which enables the access of the sulfonamide group of CEL to the side pocket of the enzyme; and (2) the selective interaction of this group with residues Gln192 and Arg513 in the side pocket. The latter is clearly illustrated in Figure 2a, which depicts the stable COX-2-CEL complex obtained from molecular dynamics (MD) simulations for human COX-2 (hCOX-2). In this complex, the pyrazole ring and its trifluoromethyl substituent are located near residues Arg120 and Tyr355 found at the entrance of the catalytic cavity of hCOX-2, while the tolyl unit occupies a deeper region of the main pocket, approaching and blocking residues Tyr348, Tyr385, and Ser530 that are involved in the enzyme’s catalytic activity. Overall, CEL occupies most of the hCOX-2 catalytic cavity, thereby hindering AA from binding and, hence, preventing subsequent conversion into the pro-inflammatory prostaglandin lipid mediators.

**Figure 1:**
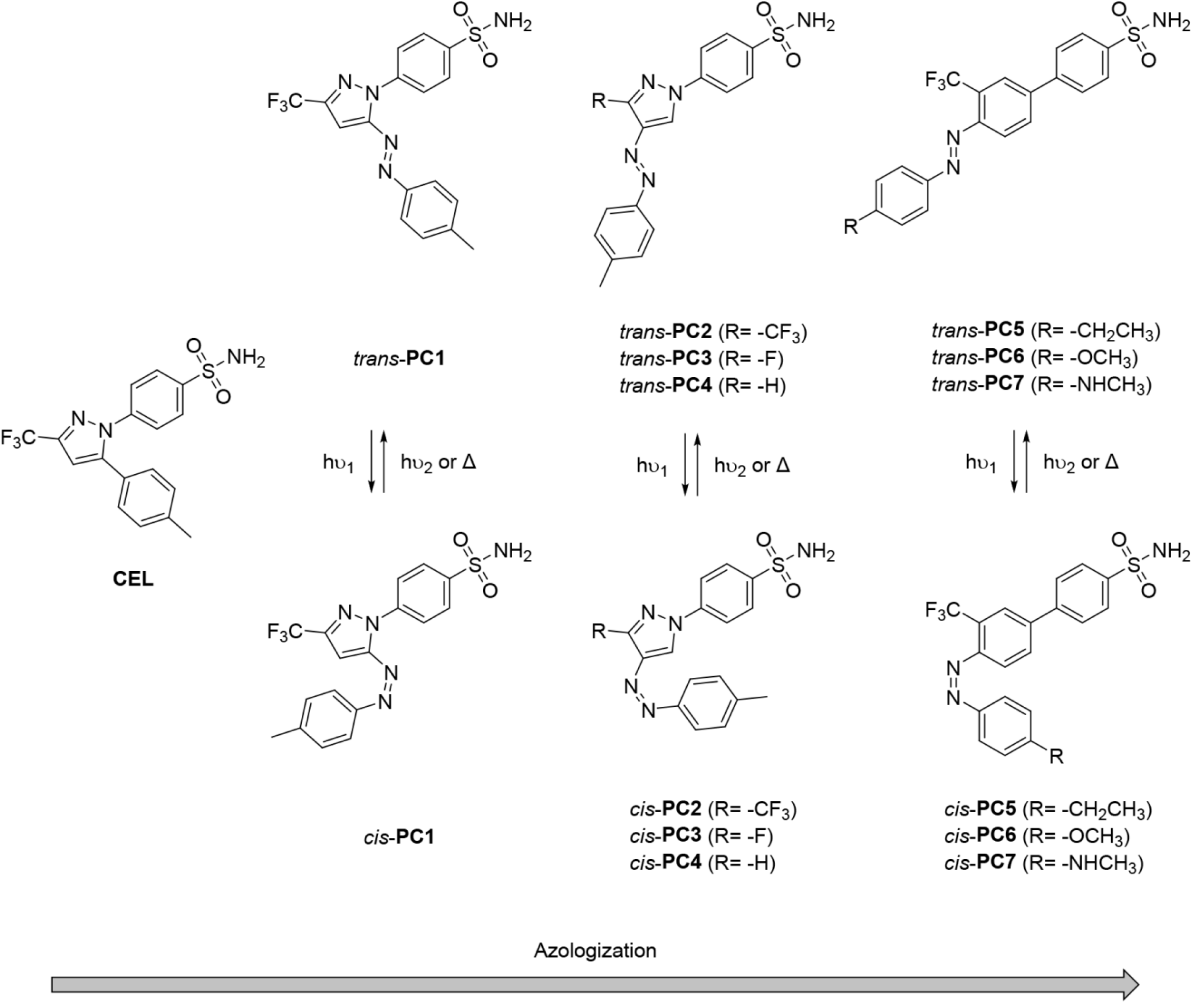
Azologization strategy explored in this work for the development of photoswitchable analogs of celecoxib (**PC1**–**PC7**) aimed at selectively inhibiting COX-2 upon photoisomerization to the *cis* form.

**Figure 2:**
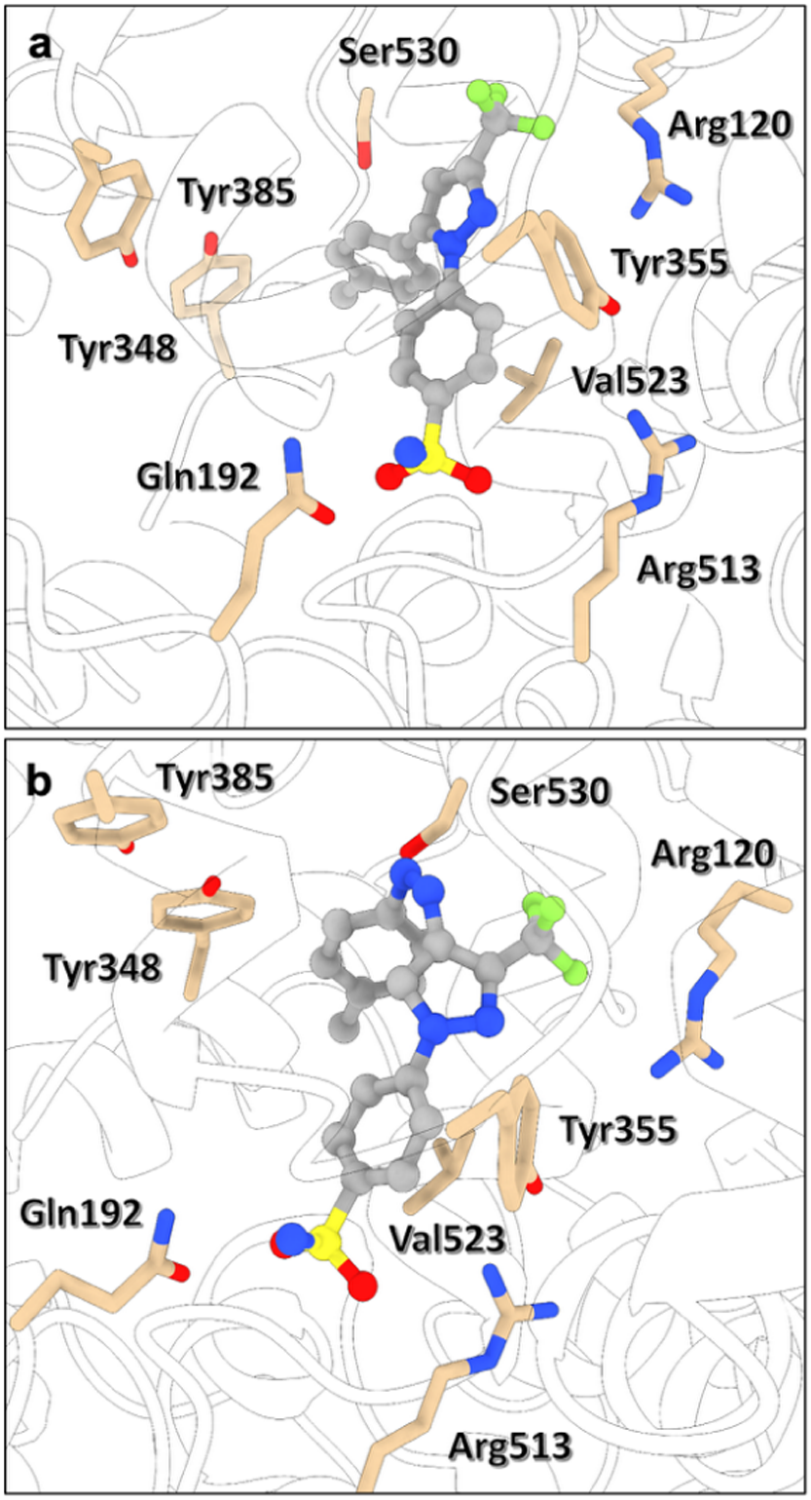
3D representation of (a) CEL and (b) *cis*-**PC2** bound to the active site of hCOX-2. Each complex structure was derived from the last snapshot of its corresponding MD simulation (see text). Key residues within the binding pocket of hCOX-2 are shown in tan, while CEL and *cis*-**PC2** appear in gray. Nitrogen atoms are depicted in blue, oxygen atoms in red, fluorine atoms in green, and the sulfur atom in yellow. Hydrogen atoms are omitted for clarity.

Based on the CEL structure, photoswitchable analogs—or photocoxibs (PCs)—were designed by azologization, a common strategy in photopharcomology that involves introducing an azoaromatic photoswitch into the core of known drugs. This modification allows the resulting azalog to reversibly interconvert between *trans* and *cis* isomers upon irradiation, inducing significant geometric changes that modulate biological activity. For PCs, two main design principles guided azologization: (1) *cis*-PCs should take a bent geometry resembling the CEL structure, thereby enabling strong binding to the catalytic site of COX-2; (2) *trans*-PCs should adopt an elongated structure that prevents favorable interactions with COX-2 active site residues. As a result, the COX-2 inhibition activity of PCs should be minimal in its most thermodynamically stable *trans* state and activated on demand upon photoisomerization to the *cis* form.

To this end, several CEL azalogs were designed (**PC1**-**PC7**, Figure 1), and their binding capabilities as *cis*-selective COX-2 inhibitors were computationally assessed. For this, molecular docking calculations were first performed to generate up to 100 binding modes inside the hCOX-2 catalytic cavity for the *trans* and *cis* isomers of **PC1**-**PC7**. From the top-ranked docking pose for each compound, MD simulations were conducted to evaluate the stability of the hCOX-2-PC complexes (Figures S1–S8) and to compare their structure with that of the hCOX-2-CEL system (Figure 2a). Finally, binding energies (ΔG_binding_) for all complexes were estimated using molecular mechanics Poisson–Boltzmann surface area (MM-PBSA) calculations, providing a quantitative measure of the difference in COX-2 affinity between the *trans* and *cis* states of all designed PCs (Figure 3 and Table S1).

**Figure 3:**
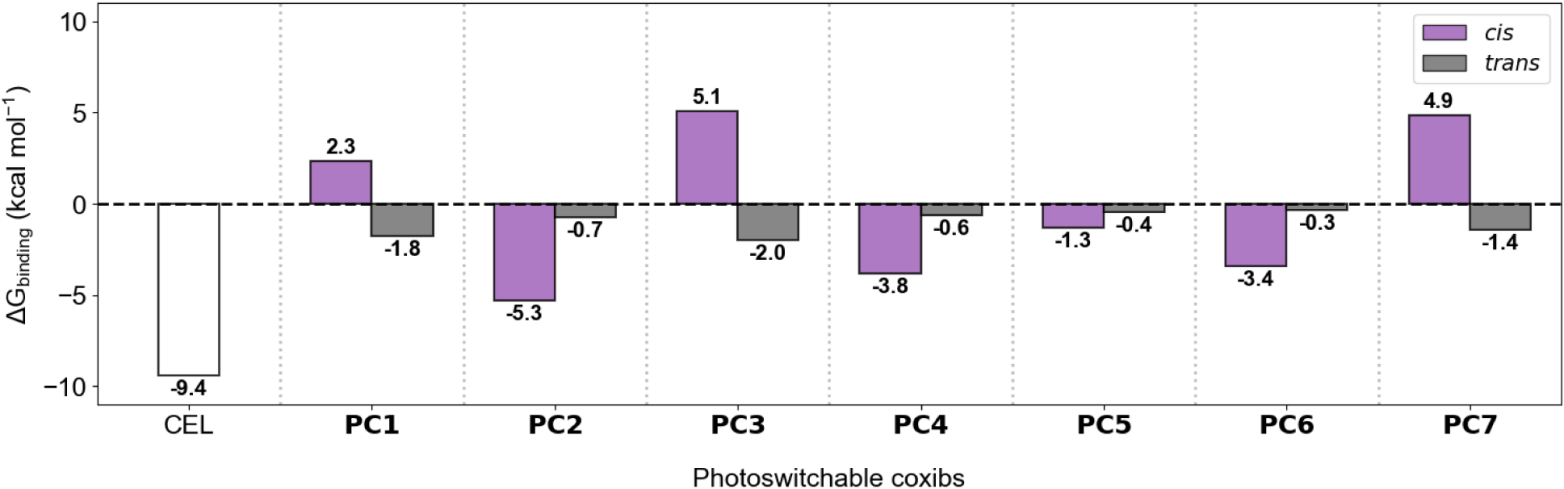
Computed values of ΔG_binding_ between hCOX-2 and the *cis* and *trans* isomers of photocoxibs **PC1**-**PC7**. As a reference, ΔG_binding_ is also provided for the hCOX-2-CEL complex.

Among all PC compounds analyzed, **PC1** exhibits the greatest structural similarity to CEL, as it was obtained by simply introducing an azo group between the pyrazole and tolyl rings of the parent drug; hence, it features a 5-phenylazopyrazole photoswitchable core (Figure 1). However, contrary to our design rationale, the *trans* isomer of this compound showed higher computed binding affinity for hCOX-2 ( ${\Delta G}_{\mathrm{binding}}^{\mathrm{trans}-PC1}=-1.8\;\mathrm{kcal}\;{\mathrm{mol}}^{-1}$ and ${\Delta G}_{\mathrm{binding}}^{cis-PC1}=+2.3\;\mathrm{kcal}\;{\mathrm{mol}}^{-1}$ ), with its complex in the catalytic cavity of the enzyme more closely resembling CEL binding mode (Figure 3 and Figure S1).

To favor a more elongated *trans* isomer structure, **PC2** was designed by moving the phenylazo substituent to position 4 of the pyrazole ring (Figure 1). This modification resulted in a clear geometric distinction between *trans*- and *cis*-**PC2**, with the latter notably resembling the CEL structure and its binding mode within hCOX-2 cavity (Figure 2b). As a result, large stability was computed for the hCOX-2-*cis*-**PC2** complex ${(\Delta G}_{\mathrm{binding}}^{cis-PC2}=-5.3\;\mathrm{kcal}\;{\mathrm{mol}}^{-1})$ , whose less favorable binding energy compared to CEL ${(\Delta G}_{\mathrm{binding}}^{\mathrm{CEL}}=-9.3\;\mathrm{kcal}\;{\mathrm{mol}}^{-1})$ might be ascribed to the partial loss of stabilizing interactions between the trifluoromethyl group of *cis*-**PC2** and residue Arg120 in the enzyme cavity (Figure 2b and Figure 3). Notably, a marked reduction in hCOX-2 binding affinity was determined for the *trans* isomer of **PC2** ( ${\Delta G}_{\mathrm{binding}}^{\mathrm{trans}-\mathrm{PC2}}$ = −0.7 kcal mol^−1^), positioning this compound as a promising photocoxib candidate (Figure S2).

Building on **PC2** as the lead compound, we next evaluated the effect of replacing the trifluoromethyl substituent on the pyrazole ring with other groups such as fluorine (**PC3**) and hydrogen (**PC4**) (Figure 1). For **PC3**, this structural modification completely reversed the binding preference of the two isomers of the system to the catalytic cavity of the enzyme, favoring the formation of the hCOX-2-*trans*-**PC3** complex ( ${\Delta G}_{\mathrm{binding}}^{\mathrm{trans}-\mathrm{PC3}}$ = −2.0 kcal mol^−1^ and ${\Delta G}_{\mathrm{binding}}^{\mathrm{cis}-\mathrm{PC3}}$ = +5.0 kcal mol^−1^) (Figure 3 and Figure S3). In contrast, **PC4** showed the desired modulation in ΔG_binding_, as its *cis* isomer exhibited the highest affinity to interact with the catalytic cavity of hCOX-2 ( ${\Delta G}_{\mathrm{binding}}^{\mathrm{trans}-\mathrm{PC4}}$ = −0.6 kcal mol^−1^ and ${\Delta G}_{\mathrm{binding}}^{\mathrm{cis}-\mathrm{PC4}}$ = −3.8 kcal mol^−1^) (Figure 3 and Figure S4). Therefore, **PC4** was also regarded as a prospective azalog derivative of CEL, although the hCOX-2–*cis*-**PC4** complex was computed to be less stable than for *cis*-**PC2**, likely due to the absence of the additional molecular interactions provided by the trifluoromethyl group.

In the last step, we investigated replacing the phenylazopyrazole photoswitch in **PC1**-**PC4** with a more conventional azobenzene photochrome, which is the principal light-responsive unit employed in photopharmacology.^32–34^ To this end, we designed photocoxibs **PC5**-**PC7** featuring a 4-(4’-sulfamoyl)­phenyl-2-trifluoromethylazobenzene core, in analogy with the substitution pattern of the pyrazole ring of lead compound **PC2** (Figure 1). **PC5**-**PC7** differ in the electron-donating *p*-substituent introduced on the opposite side of the azobenzene core—ethyl (**PC5**), methoxy (**PC6**) and *N*-methylamino (**PC7**) groups—with which we aimed to modulate the photoswitching properties of the resulting photocoxibs.^35^ However, these substituents were also found to exert a significant effect on the interaction with hCOX-2.

For **PC7**, *trans*-favored binding was observed ( ${\Delta G}_{\mathrm{binding}}^{\mathrm{trans}-\mathrm{PC7}}$ = −1.4 kcal mol^−1^ and ${\Delta G}_{\mathrm{binding}}^{\mathrm{cis}-\mathrm{PC7}}$ = +4.9 kcal mol^−1^), discouraging the use of this compound as a photoswitchable analog of CEL (Figure 3 and Figure S7). In contrast, **PC5** and **PC6** exhibited preferential binding of their *cis* isomer to hCOX-2, as targeted (Figure 3 and Figures S5–S6). Particularly noteworthy were the results for **PC6**, which displayed an altered *cis*-binding mode compared to CEL that features different locations for the trifluoromethyl and terminal phenyl groups within the enzyme cavity. Despite this, computed ΔG_binding_ values for **PC6** were comparable to those of phenylazopyrazole-based compound **PC4** $({\Delta G}_{\mathrm{binding}}^{\mathrm{trans}-\mathrm{PC6}}$ = −3.4 kcal mol^−1^ and ${\Delta G}_{\mathrm{binding}}^{cis-PC6}$ = −0.3 kcal mol^−1^), suggesting that strong *cis*-selective hCOX-2 inhibition could be achieved with azobenzene-based photocoxibs.

### Synthesis and Photochemical Characterization

Based on the computational modeling studies performed, we focused our synthetic efforts on the preparation of photocoxibs **PC2**, **PC4**, **PC5,** and **PC6** exhibiting *cis*-preferential binding to hCOX-2. The strategy employed for the construction of the diazene bond present in all candidates relied on the Baeyer–Mills reaction between aromatic amines and freshly synthesized nitrosoarenes, which has been reported to grant access to both arylazopyrazole (**PC2** and **PC4**)^36^ and azobenzene photochromes (**PC5** and **PC6**).^37^

Accordingly, we attempted the preparation of the photochromic cores of **PC2** (*trans*-**3a**) and **PC4** (*trans*-**3b**) by Mills reaction between nitroso derivative **2** and aminopyrazoles **1a** and **1b** (Scheme 1). Compounds **1a** and **1b** were previously synthesized by nitration and subsequent reduction of the corresponding commercial pyrazoles, while nitroso intermediate **2** was freshly prepared by oxidation of *p*-toluidine and later used without further purification (see Supporting Information). However, while satisfactory results were obtained for the synthesis of *trans*-**3b** (69% yield), the formation of *trans*-**3a** by Mills reaction was not observed, probably due to the reduced nucleophilicity of amine **1a** bearing an electron-withdrawing trifluoromethyl group. Attempts to prepare the nitroso derivative of **1a** to make it react with *p*-toluidine as well as other strategies explored for the construction of the arylazopyrazole photochrome of **PC2** were also unsuccessful. Consequently, we abandoned the synthesis of this compound and focused on the preparation of **PC4**. With this aim, intermediate *trans*-**3b** was allowed to react with commercially available boronic acid **4** under *N*-arylation Chan-Lam conditions^38^ using copper diacetate as a catalyst, finally affording the *trans* isomer of candidate **PC4** in 18% yield (Scheme 1). This low yield was ascribed to the cumbersome workup of the reaction necessary to get rid of the copper species.

**Scheme 1:**
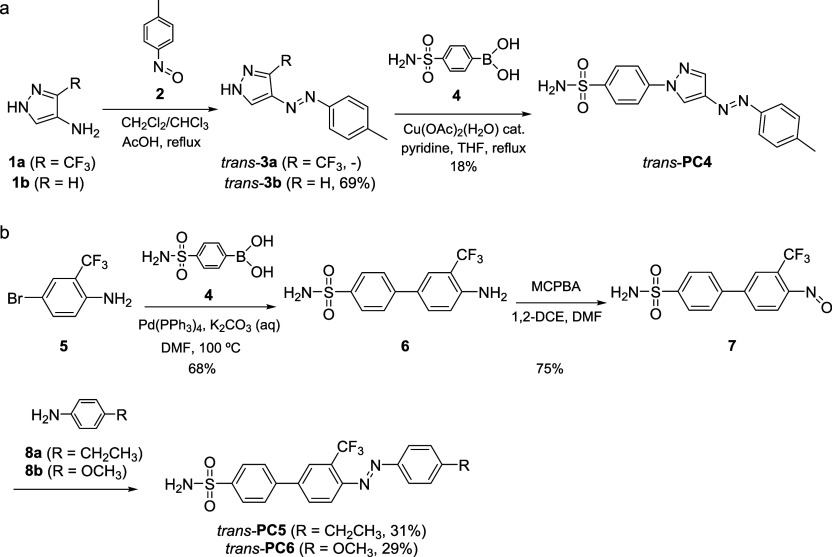
Synthesis of Photoswitchable CEL Analogues (a) **PC4** and (b) **PC5** and **PC6**

The preparation of azobenzene candidates **PC5** and **PC6** started with the coupling between commercially available amine **5** and boronic acid **4** through a Suzuki–Miyaura reaction under palladium catalysis, which afforded amine **6** in 68% yield (Scheme 1). Transformation of **6** to the corresponding nitroso intermediate **7** required for Mills reaction was achieved using *m*CPBA as the oxidant in a mixture of organic solvents. The resulting 25:75 mixture of the starting amine and the corresponding nitroso derivative was directly reacted with commercial amines **8a** and **8b** to provide the target compounds **PC5** and **PC6** in their *trans* configuration in 31% and 29% yields, respectively (Scheme 1). The structural identity of all compounds involved in the synthesis of **PC4**, **PC5,** and **PC6** was confirmed by HRMS and NMR spectroscopy (see Supporting Information).

Next, we investigated the photoswitching properties of **PC4**–**PC6** in aqueous media. According to ^1^H NMR data, these three compounds reside exclusively in their most thermodynamically stable *trans* isomers in the dark and room temperature. In this state, they exhibited very similar absorption spectra in DMSO:water mixtures, where an intense π-π* band at λ_abs,max_ ∼ 345–370 nm and a much weaker n-π* band at λ_abs,max_ ∼ 410–450 nm were identified (Figure 4a, Figure S9, Table S2). These spectral features are characteristic of *trans*-azobenzenes^35^ and *trans*-arylazopyrazoles^36,39,40^ in the absence of strong electron-donating and/or electron-withdrawing substituents, and they are in agreement with TD-DFT calculations performed at the M06–2*X*/6–31+G­(d) level for **PC4**-**PC6** (Figure S13 and Table S3). As a result, these compounds must undergo efficient *trans*→*cis* photoisomerization under UV excitation of their π-π* absorption band. Indeed, irradiation of *trans-* **PC4**-**PC6** at λ_exc_ = 365 nm led to large spectral changes that are compatible with *cis* state formation—i.e., the decrement and hypsochromic shift of the π-π* band as well as the increment of the n-π* band (Figure 4a).^35,36,39^ The efficiency of UV-induced *trans*→*cis* photoisomerization was evaluated by combined ^1^H NMR and UV–vis absorption measurements (Figures S9–S10, Table S3). As previously described,^36,39^ larger photoconversion in the photostationary state (PSS_*t‑c*_) and photoisomerization quantum yields (Φ_*t‑c*_) were measured for the arylazopyrazole photochrome of **PC4** (10:90 *trans*:*cis* ratio, Φ_*t‑c*_ = 0.22) relative to azobenzenes **PC5** (27:73 *trans*:*cis* ratio, Φ_*t‑c*_ = 0.09) and **PC6** (34:66 *trans*:*cis* ratio, Φ_*t‑c*_ = 0.13).

**Figure 4:**
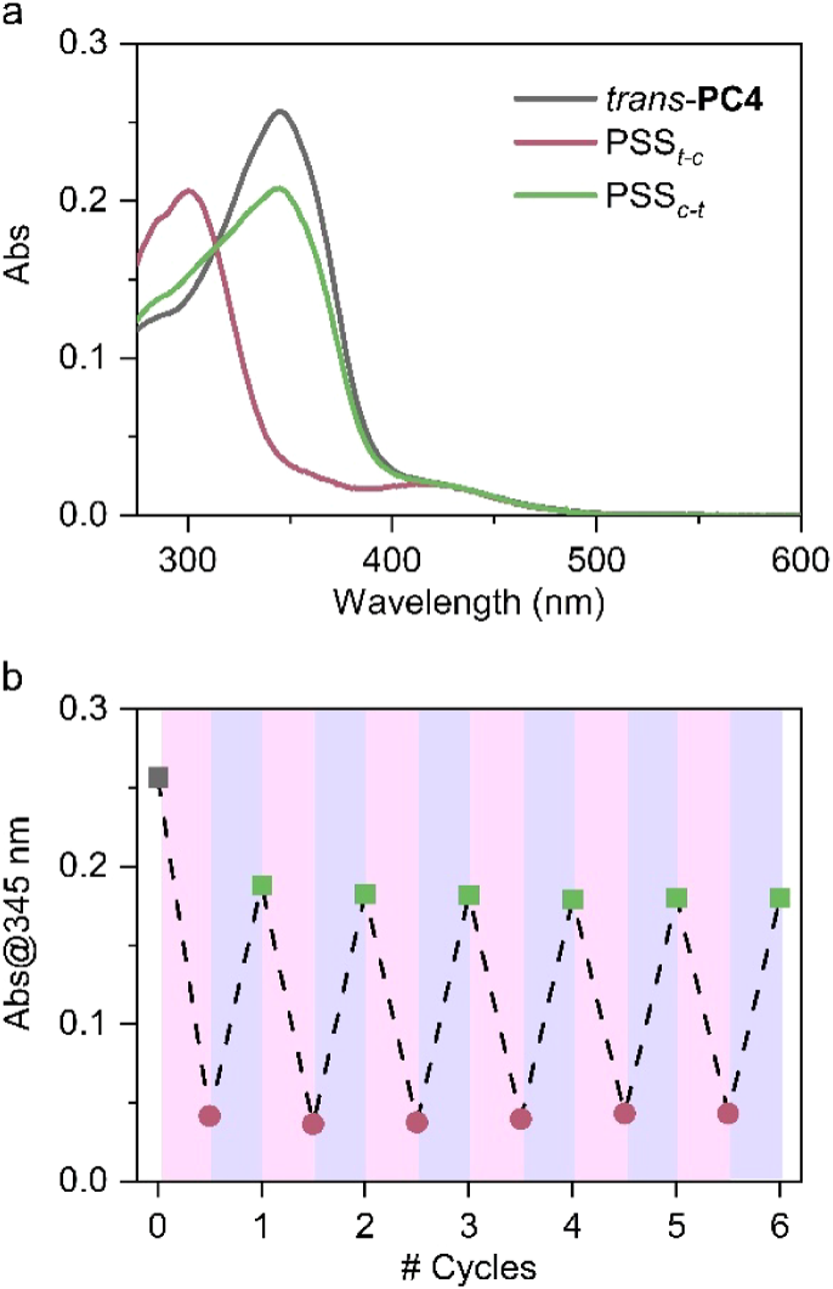
(a) Absorption spectra of *trans*-**PC4** in 75:25 DMSO:H_2_O (*c* = 8.9 μM) and of the photostationary states obtained by irradiation at λ_exc_ = 365 (PSS*_t‑c_*) and 405 nm (PSS*_c‑t_*). (b) Variation of absorbance at λ_abs_ = 345 nm upon irradiation of *trans*-**PC4** in 75:25 DMSO:H_2_O (*c* = 8.9 μM) for six consecutive cycles of UV (λ_exc_ = 365 nm, violet) and visible irradiation (λ_exc_ = 405 nm, blue).

Subsequent irradiation of the *cis* isomer of these compounds with visible light (λ_exc_ = 405 or 445 nm) resulted in *cis*→*trans* back-photoisomerization with rather high quantum yields (Φ_*c‑t*_ = 0.11–0.33), leading to PSS_*c‑t*_ equilibrium mixtures with about 70–80% content in *trans* isomer (70:30, 79:21, and 71:29 *trans*:*cis* ratios for **PC4**, **PC5,** and **PC6**, respectively) (Table S2). Consequently, reversible photoswitching between the *trans* and *cis* states of **PC4**-**PC6** could be accomplished for consecutive cycles via sequential UV and visible light irradiation without apparent signs of photodegradation (Figure 4b, Figure S11). In addition, full conversion to the initial *trans* state of these compounds could be achieved thermally by back-isomerization in the dark, although with very slow kinetics at room temperature (*t*_1/2_ = 37, 17, and 10 h, Figure S12). As a result, a high *cis* content could be preserved for hours in UV-irradiated mixtures of **PC4**-**PC6**, a behavior that was exploited in the biological experiments shown below.

In these experiments, azo-based PCs could be affected by intracellular reductive agents such as glutathione, thereby altering their light-induced biological response.^41,42^ For this reason, we assessed the stability of **PC4**-**PC6** under reductive aqueous conditions (10 mM glutathione and 5 mM tris­(2-carboxyethyl)­phosphine in DMSO:H_2_O mixtures) for both their initial *trans* isomer and their PSS_*t‑c*_ state generated at λ_exc_ = 365 nm. After incubation for 24 h, no significant changes were observed by ^1^H NMR and UV–vis absorption spectroscopy, indicating high resistance to biological reduction (Figure S14).

### *In Vitro* Enzyme Inhibition Assays

To evaluate the biological performance and potential isoform selectivity of the photocoxibs synthesized, we assessed their inhibitory activity against both COX isoforms using the fluorometric Abcam COX-1 and COX-2 screening kits under controlled dark and UV-irradiated (365 nm) conditions; i.e., for their *trans* isomers and *cis*-enriched PSS_*t‑c*_ mixtures, respectively. Inhibition was quantified at a single, relatively high concentration (45 μM), corresponding to the highest soluble concentration under assay conditions. This screening strategy ensured reliable detection of target engagement and minimized false negatives, providing a rapid and robust functional readout sufficient to prioritize compounds for subsequent cellular and *in vivo* studies at physiologically relevant concentrations.

**PC4**–**PC6** were compared alongside the reference inhibitors SC-560 (COX-1) and CEL (COX-2), which served as benchmarks for potency and isoform selectivity. As expected, the reference inhibitors reproduced their characteristic profiles, validating the assay, and establishing a meaningful activity scale for interpreting the photocoxib results. Across the full data set, **PC4**-**PC6** exhibited minimal inhibition of COX-1, regardless of illumination state—except for **PC5**—confirming weak affinity for the constitutive isoform (Figure 5). A different behavior was observed for COX-2, where the three inhibitors increased their activity upon *trans*→*cis* photoisomerization, in agreement with our computational simulations. **PC4** showed modest enhancement upon irradiation, rising from negligible activity in the dark to ∼9% inhibition under 365 nm light. In contrast, **PC5** and **PC6** demonstrated more pronounced light-induced increases in COX-2 inhibition, with activity more than doubling upon photoisomerization: from ∼12% to ∼27% for **PC5**, and from ∼2% to ∼19% for **PC6**. These responses represent the strongest photocontrolled effects within the series, suggesting that the *cis* isomers of **PC5** and **PC6** adopt geometries more compatible with COX-2 binding. By comparison, the *trans* isomers of all three photocoxibs remained weak inhibitors, as anticipated from their elongated, sterically mismatched conformations.

**Figure 5:**
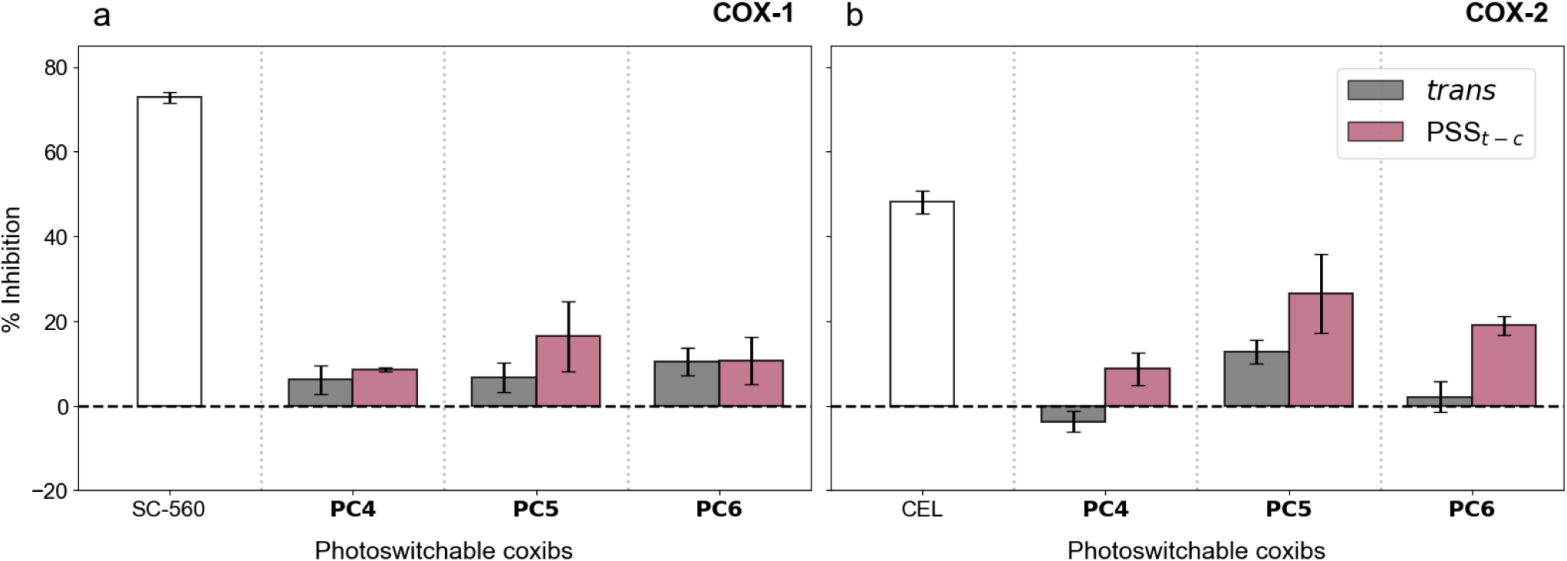
(a) COX-1 and (b) COX-2 inhibition measured using Abcam fluorometric COX assay kits. Bars show the percentage inhibition calculated from end point relative fluorescent unit values after blank subtraction and averaging of technical replicates. Error bars represent propagated standard deviation (SD). SC-560 and CEL are shown only in dark conditions. Photoswitchable inhibitors **PC4**-**PC6** were tested in the dark (*trans*) and after previous 365 nm irradiation to generate the corresponding *cis*-enriched PSS*_t‑c_* mixtures.

Collectively, these results confirm that embedding a photoswitch within the coxib scaffold enables reversible, light-induced inhibition of COX-2 while almost sparing COX-1. In particular, **PC5**, **PC6,** and, in a lesser extent, **PC4** satisfy the two essential design criteria for photopharmacological anti-inflammatory agents: low basal activity in the dark-adapted *trans* state, which minimizes off-target interactions, and robust activation upon irradiation, which enables spatiotemporal precision in COX-2 blockade. Having established their photocontrolled inhibitory profiles *in vitro*, we next sought to determine whether this behavior translates to more complex biological contexts. Therefore, we evaluated the cellular activity of the photocoxibs in macrophages and assessed their ability to modulate inflammation *in vivo* using zebrafish, two complementary models that allow testing both potency and light-dependent functional outcomes under physiologically relevant conditions.

### Photomodulation of PGE_2_ Production in Activated Murine Macrophages

The macrophage-driven production of PG, particularly PGE_2_, makes these cells an ideal platform for investigating the modulation of the COX pathway under both physiologically relevant (COX-1) and inflammatory (COX-2) conditions. J774 murine macrophage cell culture is a well-established *in vitro* assay used to evaluate the potency and selectivity of NSAIDs against COX-1 and COX-2 isoforms.^43,44^ Herein, we applied this methodology to test the inhibitory effects of the two isomers of **PC4**-**PC6** on COX-1 and COX-2. Experiments were conducted at photocoxib concentrations of 0.1, 1, and 10 μM, since cell viability studies revealed that the highest concentration tested for all compounds (50 μM) was toxic to the cells (data not shown).

Under resting conditions, J774 macrophages only express the COX-1 enzyme. To evaluate the effect of the compounds on COX-1 activity, J774 macrophages were stimulated with AA (15 μM) for 30 min after a 15-min preincubation with *trans-* **PC4**-**PC6** or their *cis*-enriched PSS_*t‑c*_ mixtures, which were previously obtained upon irradiation at 365 nm (10 μM). Indomethacin was used as the reference compound (10 μM). Stimulation of J774 macrophages with AA (15 μM) for 30 min induced a significant increase in PGE_2_ levels compared with unstimulated control cells. The three *trans*-PC analogues, as well as their PSS_*t‑c*_ mixtures, did not show any detectable effect on PGE_2_ production by COX-1. Indeed, PGE_2_ levels in the supernatants of cells pretreated with all PC samples were similar to those observed in control cells (Figure 6a). As expected, a strong reduction was instead observed in the supernatant of cells pretreated with indomethacin, a COX-1 inhibitor (75% inhibition). These results indicate that neither the *trans* nor *cis* isomers of **PC4**–**PC6** inhibit COX-1, in agreement with enzymatic studies and as desired for selectivity purposes.

**Figure 6:**
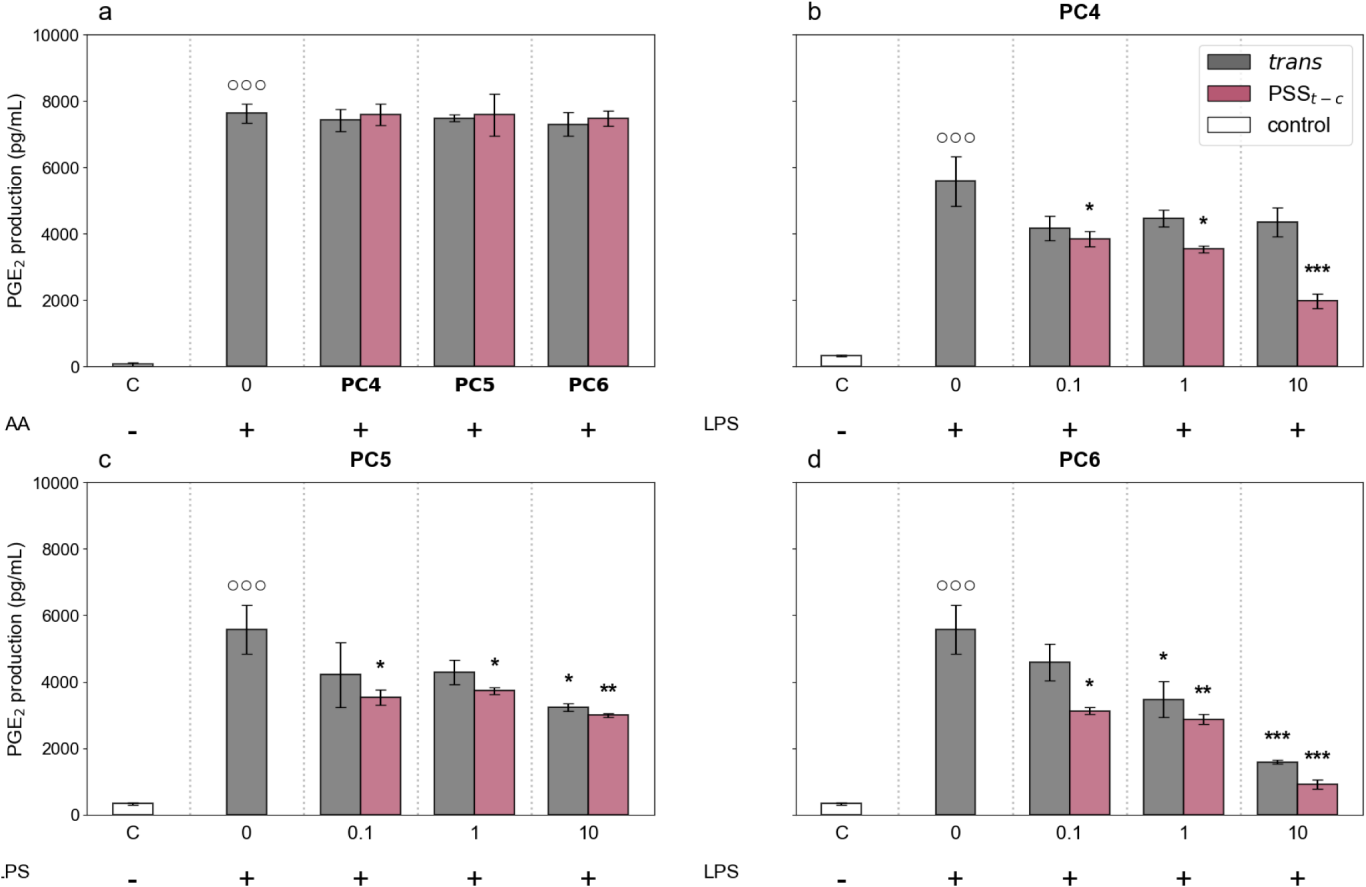
(a) Inhibition of COX-1-mediated PGE_2_ production in J774 cells, which were pretreated with *trans*-**PC4**–**PC6** (dark) or their *cis*-enriched PSS*_t‑c_* mixtures (365 nm) (10 μM) for 15 min and then incubated with arachidonic acid (AA, 15 μM) for 30 min. Control experiments where cells were not incubated with AA or PC samples are indicated as C and 0, respectively. (b-d) Inhibition of COX-2-mediated PGE_2_ production in J774 cells, which were incubated with the *trans* isomer (dark) or *cis*-enriched PSS*_t‑c_* mixture (365 nm) of (b) **PC4**, (c) **PC5,** and (d) **PC6** (0.1–10 μM), and then stimulated for 24 h with LPS (10 μg mL^–1^). Control experiments where cells were not incubated with LPS or PC samples are indicated as C and 0, respectively. In all cases, the supernatants were collected for the measurement of PGE_2_ levels by ELISA assay. Values represent means ± standard error of the means (SEM). Data were analyzed by one-way ANOVA plus Bonferroni. Statistical significance is reported as follows: °°° *p* < 0.001 vs unstimulated cells (C); *p < 0.05, **p < 0.01 and ***p < 0.001 vs LPS alone.

Next, to evaluate the effect of photocoxibs on PGE_2_ production by COX-2, J774 macrophages were stimulated with lipopolysaccharide (LPS, 10 μg mL^–1^) for 24 h in the presence or absence of the tested compounds. Treatment with LPS induced a significant increase in PGE_2_ production as a result of the inflammatory response and, therefore, COX-2 induction promoted in the macrophages.^45^ CEL (0.01 μM) was used as the reference COX-2 selective inhibitor in these experiments, which caused 92% decrement in PGE_2_ production.

In contrast, the capacity to inhibit COX-2-mediated PGE_2_ production by the dark-adapted *trans* isomer of **PC4**-**PC6** was found to be much smaller, only reaching significance for **PC5** at 10 μM and **PC6** at 1 and 10 μM (Figure 6b–d). Thus, we determined that ${\mathrm{IC}}_{50}^{\mathrm{COX‐2}}$ must be greater than 10 μM for *trans-* **PC5** and *trans-* **PC4**, while only *trans-* **PC6** showed measurable potency ${(\mathrm{IC}}_{50}^{\mathrm{COX‐2}}=2.14\,\;\mu M)$ and selective COX-2 inhibitor character ${(\mathrm{IC}}_{50}^{\mathrm{COX‐1}}/{\mathrm{IC}}_{50}^{\mathrm{COX‐2}}>4.6)$ (Table 1). More importantly, we observed that COX-2 inhibition was light-dependent for these compounds. A statistically significant inhibition of PGE_2_ production in LPS-stimulated macrophages was observed for all *cis*-enriched PSS_*t‑c*_ obtained by irradiation at 365 nm at all tested concentrations (0.1, 1, and 10 μM) (Figure 6b–d). Among them, *cis*-**PC5** exhibited the lowest potency, as 50% inhibition of PGE_2_ production was not achieved even at the highest tested concentration. Conversely, *cis-* **PC4** and *cis-* **PC6** showed increased potency, as evidenced by a clear reduction of ${\mathrm{IC}}_{50}^{\mathrm{COX‐2}}$ values for their irradiated PSS_*t‑c*_ mixtures relative to the *trans* isomers: from **>** 10 μM to 2.4 μM for **PC4**, and from 2.14 μM to 0.4 μM for **PC6**, which must be considered lower limits of the COX-2 activity modulation attained between the *trans* and *cis* states of these compounds due to their incomplete *trans*→*cis* photoisomerization at 365 nm (Table 1). In spite of this, these data prove that photocoxibs behave as COX-2-selective, light-regulated anti-inflammatory agents, corroborating our computational predictions that the *cis* form of **PC4**, **PC6,** and, in a lesser extent, **PC5** should bind to the active cavity of the enzyme with higher affinity than their *trans* counterpart. In agreement with enzymatic assays, the best results were achieved for **PC6**, which upon irradiation at 365 nm exhibits COX-2 inhibition with 5-fold more potency than the initial *trans* isomer and an *in vitro* selectivity index higher than 25 over COX-1 (Table 1).

**Table 1:** *In Vitro* COX-1 and COX-2 Inhibitory Activity of Photocoxibs in J774 Murine Macrophage Assay

		*trans*				PSS_*t‑c*_ ^a^			
PC	[PC] (μM)	COX-2 inh. (%)^b^	$IC_{50}^{COX-2}$ (μM)^c^	$IC_{50}^{COX-1}$ (μM)^c^	COX-1/COX-2 selectivity^d^	COX-2 inh. (%)^b^	$IC_{50}^{COX-2}$ (μM)^c^	$IC_{50}^{COX-1}$ (μM)^c^	COX-1/COX-2 selectivity^d^
**PC4**	10	21.6	>10	>10	-	64.3	2.4	>10	
	1	19.9				36.7			>4.1
	0.1	25.2				31.0			
**PC5**	10	41.6	>10	>10	-	45.9	>10	>10	
	1	23.2				32.4			-
	0.1	24.4				35.8			
**PC6**	10	71.1	2.1	>10	>4.6	82.9	0.4	>10	
	1	37.6				48.1			>25
	0.1	17.8				43.3			

a *Cis*-enriched mixtures obtained by irradiation of the *trans* isomers at 365 nm.

b Percentage of inhibition of COX-2-mediated PGE_2_ production by test compounds with respect to control samples. The mean values of 3 experiments are given.

c IC_50_ values were calculated by fitting the experimental data with a sigmoidal dose–response equation (variable slope) using the GraphPad software.

d *In vitro* COX-2 selectivity index obtained as ${\mathrm{IC}}_{50}^{\mathrm{COX‐1}}/{\mathrm{IC}}_{50}^{\mathrm{COX‐2}}$ .

The fact that larger inhibition modulation between the two states of **PC6** was determined in LPS-stimulated J774 macrophages relative to enzymatic measurements could be ascribed to the fundamental differences between both types of experiments. In cellular environments, such as LPS-stimulated macrophages, COX-2 is membrane-bound, can form oligomeric or functional complexes, and may undergo post-translational modifications. These factors alter the enzyme conformation and the accessibility of the active site, often enhancing inhibitor binding compared to the recombinant protein used in enzymatic assays. Furthermore, COX-2 and COX-1 enzymatic experiments primarily measure the peroxidase activity of enzyme rather than the entire cyclooxygenase catalytic cycle. Consequently, they may underestimate inhibition if *cis-* **PC6** primarily interferes with the cyclooxygenase reaction or requires the native membrane-bound conformation of COX-2 to exert its full effect. Furthermore, *cis-* **PC6** may exert additional modulatory effects on upstream inflammatory pathways, such as NF-κB or MAPK signaling, or it may influence the mobilization of AA through cPLA2 activity. These indirect mechanisms can substantially reduce prostaglandin production, thereby amplifying the functional inhibition observed in cellular assays.

### *In Vivo* Evaluation of Anti-Inflammatory Activity on Leukocyte Migration

To investigate the anti-inflammatory potential of photocoxibs *in vivo*, we employed the zebrafish tail-wounding assay, a well-established model that triggers an acute inflammatory response.^46,47^ Following tail amputation, leukocyte accumulation occurs at the wound site; thus, compounds with anti-inflammatory activity can be identified by their ability to reduce immune cell migration to this area. After amputation, larvae were treated for 6 h with either DMSO (vehicle control), CEL, or photocoxibs in their dark-adapted *trans* state or their PSS_*t‑c*_ obtained upon irradiation at 365 nm. Leukocytes recruited to the wound site were subsequently visualized by Sudan Black staining. Based on the results obtained *in vitro* in activated murine macrophages, these experiments were only conducted for the most promising compounds **PC4** and **PC6**.

We compared the tail regions of wounded larvae with those of uncut control embryos (Figure 7a–b). Due to the high variability in the inflammatory response, both between and within individuals, we classified larvae from all experimental groups into four categories based on the number of leukocytes that migrated to the wound area relative to uncut controls (Figure 7c). The categories were defined as follows: class 1, 0–9 leukocytes; class 2, 10–18 leukocytes; class 3, 19–27 leukocytes; and class 4, > 27 leukocytes (Figure 7d–g).

**Figure 7:**
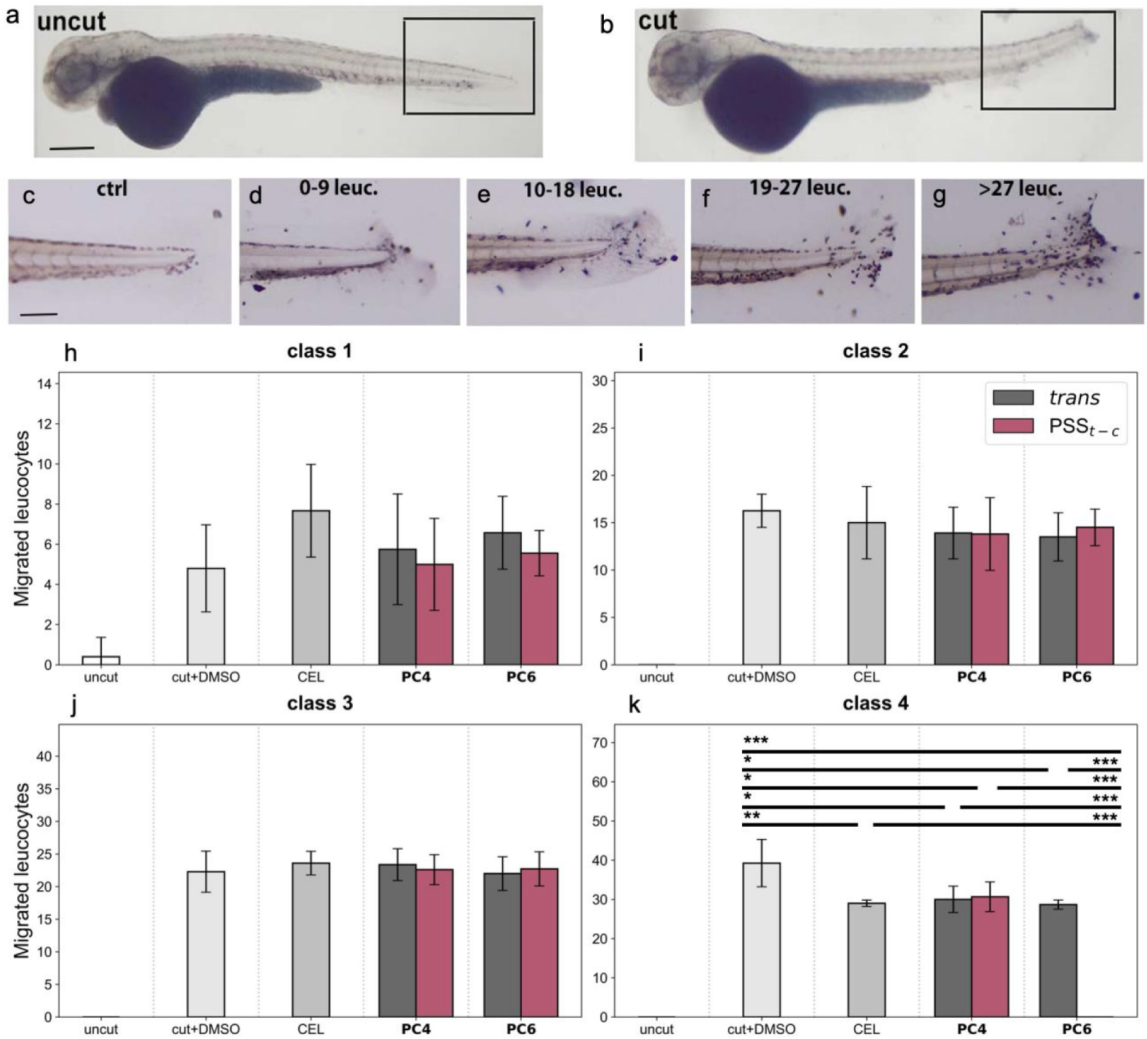
(a–b) Representative images of (a) an uncut zebrafish larva and (b) a zebrafish larva after tail wounding. (c–g) Region of interest of the tail of (c) an uncut zebrafish larva and (d-g) zebrafish larvae after tail wounding, belonging to (d) class 1, (e) class 2, (f) class 3, and (g) class 4. (h–k) Number of leukocytes migrated to the wound site at 6 hpa, after treatment with 50 μM of CEL or **PC4**-**PC6**, in their *trans* configuration or *cis*-enriched PSS*_t‑c_* mixtures after irradiation at 365 nm. Data are shown as mean ± SD of three independent experiments and are depicted separately for (h) class 1, (i) class 2, (j) class 3, and (k) class 4 larvae. Missing bars—i.e., uncut in graphs i–k, **PC6** PSS*_t‑c_* in graph k—indicate the absence of larvae belonging to the corresponding class. Statistical significance was assessed by applying the ordinary one-way ANOVA with Tukey post hoc correction (*p < 0.05, **p < 0.01, ***p < 0.001).

Since photocoxibs had never been tested in zebrafish before, we first evaluated different concentrations of the compounds by assessing their effects on leukocyte migration to the wounded area. We selected a dose range from 10 μM to 100 μM, based on previously published studies on CEL administration in zebrafish larvae.^48–50^ As shown in Figure S15, both CEL and PCs exhibited a dose-dependent anti-inflammatory effect, with a reduction in the number of larvae classified as class 3 and 4 and an increase in those classified as class 1 and 2; i.e., each compound induced a dose-dependent decrease in leukocyte recruitment to the wound site. However, differences in compound efficacy were observed, with the *cis*-enriched PSS_*t‑c*_ mixture of **PC6** showing the strongest effect at 100 μM. To allow comparison among the different compounds, we selected the intermediate concentration of 50 μM for subsequent experiments.

In larvae classified as classes 1, 2, and 3, which exhibited a mild inflammatory response, treatment with CEL or photocoxibs did not cause significant changes in the number of leukocytes migrating to the wound area (Figure 7h–j). In contrast, in class 4 larvae, which displayed a strong inflammatory response, all compounds significantly reduced leukocyte accumulation at the wound site compared with control larvae (Figure 7k). Notably, the *cis*-enriched PSS_*t‑c*_ mixture of **PC6** exerted the most potent anti-inflammatory effect, completely abolishing leukocyte migration to the wound site, reaching levels comparable to uncut, noninflamed larvae. These findings indicate that both CEL and the photocoxibs possess *in vivo* anti-inflammatory activity, with **PC6** being the most effective compound after *trans*→*cis* photoisomerization. Therefore, as also demonstrated *in vitro*, **PC6** behaves as a light-modulated NSAID *in vivo*, and it displays higher efficacy under the highest inflammation conditions, where it is most needed.

## Conclusions

In this work, we pioneered the development of photocoxibs, which are the first photoswitchable COX-2 inhibitors capable of producing light-controlled anti-inflammatory responses. By introducing azoaromatic photoisomerizable units into the structure of CEL, we designed and computationally evaluated a library of light-controlled derivatives. For several of these compounds, our simulations predicted optimal photopharmacologial behavior, with enhanced affinity for the catalytic site of COX-2 in their photoinduced *cis* state compared to the initial, dark-adapted *trans* isomer. Three of these photocoxib candidates (**PC4**-**PC6**) were successfully synthesized and photochemically characterized, exhibiting efficient, reversible *trans*-*cis* photoisomerization upon irradiation with UV and visible light. Enzymatic assays in solution and *in vitro* experiments using murine macrophage cell cultures confirmed that **PC4**-**PC6** retained the key pharmacological features of CEL: negligible inhibition of constitutive COX-1 and robust, concentration-dependent effects on induced COX-2 activity. Importantly, their COX-2 inhibitory responses were found to be light-dependent, with **PC6** showing up to 9- and 5-fold potency enhancements upon *trans*→*cis* photoisomerization in enzymatic and macrophage studies, respectively. *In vivo* testing in a zebrafish model of acute inflammation further demonstrated the desired light-modulated NSAID behavior, as **PC6** significantly reduced leukocyte recruitment around the wound site when administered in its photoinduced *cis* form. Overall, these results highlight the potential of photopharmacology to aid in developing next-generation anti-inflammatory treatments designed to minimize the adverse side effects of current NSAIDs.

The eventual therapeutic application of photoswitchable coxib analogs would, however, require further optimization, where two key challenges should be addressed. First, their action spectra must be red-shifted toward the near-infrared-I window (650 nm–900 nm) to enable higher penetration depth in tissues with lower phototoxicity. To this end, the photoswitching units of **PC4**, **PC5,** and **PC6** could be replaced with other azo derivatives exhibiting lower photoexcitation energies (e.g., *ortho*-substituted azobenzenes, push–pull azobenzenes, azonium ions, or BF_2_-coordinated azo groups).^51–53^ Second, a larger increase in inhibitory potency after *trans*→*cis* photoisomerization would be necessary to produce high therapeutic efficacy. Therefore, iterative molecular design modifications of the structure of coxib azalogs must be performed, which we are currently exploring with the aid of machine learning-based computational methods.

## Supplementary Material



## Data Availability

The data set underlying this work is available free of charge at https://dataverse.csuc.cat/dataverse/with a persistent DOI: 10.34810/data3156. The data set includes computational data (molecular docking, molecular dynamics simulations, MM-PBSA binding energies, and TD-DFT calculations), synthetic and spectroscopic characterization data (NMR, IR, and HRMS), photochemical characterization data, and biochemical, cell biology, and *in vivo* results for photocoxibs **PC1**−**PC6**.

## Notes

Computational and experimental procedures and additional characterization data for photocoxibs (PDF)
