# Supplementary material for: Photoswitchable COX-2-Selective Inhibitors as Light-Regulated Anti-Inflammatory Agents
Source: J Am Chem Soc. 2026 Apr 28;148(18):19226–37. doi: 10.1021/jacs.6c03529 (PMC13209505; doi:10.1021/jacs.6c03529)
Supplement: Supplementary file 1 [file ja6c03529_si_001.pdf]

Supporting Information for

Photoswitchable COX-2-Selective Inhibitors as Light-Regulated Anti-Inflammatory Agents

Amanda Morales,[1,‡] Alejandro Cruz,[2,‡] Álex Pérez-Sánchez,[1] Danilo D'Avino,[3] Gaia Galassi,[4] Erica Ginevra Milano,[8] Irene Bernareggi,[8] Carla Arenós-Bach,[1] Giovanni Grazioso,[8] Ramon Alibés,[1] Jordi Hernando,[1] Pau Gorostiza,[5,6,7] Antonietta Rossi,[3,*] Anna Pistocchi,[4,*] Carlo Matera,[8,*] Félix Busqué,[1,*] Àngels González-Lafont[1,*] and José M. Lluch[1]

‡ A.M. and A.C. contributed equally.
* Corresponding authors: anna.pistocchi@unimi.it, antrossi@unina.it, carlo.matera@unimi.it, felix.busque@uab.cat, angels.gonzalez@uab.cat

[1]Departament de Química, Universitat Autònoma de Barcelona, 08193 Bellaterra, Barcelona, Spain
[2]Departament d'Enginyeria Química (EQ), ETSEIB, Universitat Politècnica de Catalunya - BarcelonaTech (UPC), Campus Sud, Edif. PG, Av. Diagonal, 647, 08028 Barcelona, Spain
[3]Department of Pharmacy, School of Medicine and Surgery, University of Naples Federico II, 80131 Naples, Italy
[4]Department of Medical Biotechnology and Translational Medicine, University of Milan, L.I.T.A., Via Fratelli Cervi 93, Segrate, 20054 Milano, Italy
[5]Catalan Institution for Research and Advanced Studies (ICREA), 08010 Barcelona, Spain
[6]Institute for Bioengineering of Catalonia (IBEC), The Barcelona Institute for Science and Technology, 08028 Barcelona, Spain
[7]Biomedical Research Networking Center in Bioengineering, Biomaterials, and Nanomedicine (CIBER-BBN), 28029 Madrid, Spain
[8]Department of Pharmaceutical Sciences, University of Milan, 20133 Milan, Italy

CONTENT

1. Materials and methods S2

2. Computational design of photoswitchable celecoxib analogues S9

3. Synthesis of photoswitchable celecoxib analogs S16

4. Photochemical characterization of PC4, PC5 and PC6 S23

5. *In vivo* evaluation of anti-inflammatory activity on leukocyte migration S31

6. Spectra of selected molecules S32

7. References S59

1. Materials and methods

1.1. Computational methods

1.1.1. Computational Protein Setup. The molecular structure of the human cyclooxygenase-2 (hCOX-2) was obtained from the crystallographic structure extracted from the Protein Data Bank (PDB) with code 5F1A.[1] After cleaning the system of any crystallography-related compounds, we built an hCOX-2 system. The protonation states were predicted using the H++software[2-5] at a pH of 7.00 for titratable residues. This structure was combined with celecoxib via molecular docking and MD simulations to produce our working structure: a coxib-adapted hCOX-2 structure.

1.1.2. Molecular Docking Studies. The GOLD software[6] was used to perform docking calculations for each system. From the coxib-adapted hCOX-2 structure we calculated 100 binding poses in a 26 Å sphere centered at Arg513, a residue that sets the dimensions of the side pocket of hCOX-2. The ChemScore function was employed to estimate the binding score of the different possible candidates for our study. The docking poses were generated with the protein held fixed, but with maximum ligand's flexibility.

1.1.3. Molecular Dynamics Simulations. The simulations were performed under the recommended AMBER[7-9] protocol for this type of system. The force field parameters for the heme group were previously obtained by Cebrián-Prats et al. All ligands in this work have been parametrized with the antechamber and parmchk2 modules of AmberTools.[6-8] The Merz-Kollman RESP procedure was employed to predict the atomic charges of the molecules using the B3LYP/6-31G(d) level of theory in Gaussian16 software.[10] For the parameters of the azo group, we adopted those reported by Duchstein et al.[11] To combine the different molecular structures (hCOX-2 and photoswitchable candidates) and create the topology and coordinates files, tLeap was used. Next, the system was solvated with a TIP3P pre-equilibrated orthorhombic water box and neutralized with chlorine and sodium ions. The resulting systems contain approximately 100,000 atoms. We performed the simulations with the AMBER20[6-8] GPU-accelerated of pmemd adapted for CUDA. The MD protocol was split into five phases: minimization, NPT equilibration, NVT equilibration and NVT production. A total of 22,000 minimization steps were performed to remove bad contacts and relax the system under the steepest-descent algorithm. During the first 6,000 steps, solvent and ions were relaxed while harmonic restraints were applied to all enzyme and ligand atoms with a force constant value of 5.0 kcal/mol·Å^2. During the second 6,000 steps, the restrictions were deprecated to backbone and heavy atoms. The last 10,000 steps of minimization were performed without any restraint. Next, a 200 ps heating stage, with restraints on backbone and heavy atoms using a 5.0 kcal/mol·Å^2 force constant, was performed to adapt the temperature from 0 K to 300 K by Langevin dynamics. In a 1-ns NPT phase that follows the heating step and maintains the same constraints, the temperature and pressure were fixed to 300 K and 1 bar, respectively. The

system volume was adjusted to reach a density value of 1 g/cm^3 with the Barendsen barostat. Finally, before starting production, a 10-ns equilibration stage was performed under constant temperature and constant volume (NVT ensemble). No harmonic restraints were applied at this phase. With a 2-fs time step, 200 ns of NVT production for each system were run to study the system's behavior under stable conditions.

1.1.4. Binding Energy Calculations via MM-PBSA. The Gibbs binding energies for each ligand were estimated using Molecular Mechanics Poisson-Boltzmann Surface Area (MM-PBSA) implemented as a module in AmberTools[6] software:

$$\Delta G_{binding} = G_{EL} - (G_E + G_L) \tag{1}$$

where EL, E and L refer to the enzyme-ligand complex, enzyme and ligand, respectively. Each of these terms includes the internal energy and the entropy in gas phase, and the solvation Gibbs energy.

$$G_i = G_{gas,i} + G_{sol,i} = \langle E_{MM,i} \rangle - TS_i + G_{sol,i} \tag{2}$$

In the present work we used the single-trajectory approach where only the canonical ensemble of the complex was actually simulated, while the ensembles of the enzyme and the ligand were created by removing the appropriate atoms in each case. This way, the bonded terms of E_{MM} are identical in the bound and the unbound molecular systems and they cancel exactly in equation 1. This approach ignores any structural reorganization of the ligand and the enzyme upon ligand binding. Many snapshots were chosen over the MD simulation of the complex, and all solvent molecules were removed from each one, because the implicit PBSA solvent model is already used to determine the solvation energies.

Combining equations 1 and 2, the binding free energy can be written as:

$$\Delta G_{binding} = \Delta G' - T\Delta S = \langle E_{el,EL} \rangle + \langle E_{vdw,EL} \rangle - T\Delta S \quad + \Delta G_{sol} \tag{3}$$

$\langle E_{el,EL} \rangle$ and $\langle E_{vdw,EL} \rangle$ are the ensemble-averaged enzyme–ligand interaction energies obtained from the electrostatic and van der Waals energy terms of the selected snapshots, respectively. The solvent was modelled with a continuum model using the recommended dielectric constant of $\epsilon = 1$ for non-polar molecules. The non-polar solvation terms were assumed to be linear-dependent with the solvent accessible surface area (SASA) and the polar solvation terms were obtained from solving the linearized Poisson-Boltzmann equation.

Entropy contributions were obtained from the interaction entropy method[12] using the code from A. Cruz et al.,[13] instead of using the most common approach based on normal modes analysis.

MM-PBSA binding energies were obtained for all ligands in the study using the last 50 ns of each MD trajectory.

1.1.5. TD-DFT Calculations. All quantum calculations were done using Gausian16[9] software. We performed geometry optimization and vibrational frequency analysis at the M06-2X/6-31+G(d) level of theory. Solvation effects of water were included via the implicit solvent model SMD. Next, we calculated two vertical transitions using the time-dependent density functional theory (TD-DFT) with the same functional and basis set. The first calculation was performed with non-equilibrium solvation effects followed by a second with equilibrium solvation.

1.2. Materials and methods for synthetic procedures

Commercially available reagents were used as received and following the corresponding instructions of the suppliers. Solid reagents were purged with argon atmosphere. All solvents were degasified with argon gas. The reactions were monitored by analytical thin-layer chromatography (TLC) using silica gel 60 F254 pre-coated aluminium plates (0.25 mm thickness) or by nuclear magnetic resonance spectra (NMR). Flash column chromatography was performed using silica gel (230-400 mesh). TLC spots were detected under UV light and/or by charring with a $KMnO_4$/KOH aqueous solution or Vanillin solution. ^{1}H NMR spectra were performed on Bruker Avance NEO 300 MHz, Bruker Avance NEO 400 MHz, and Bruker Avance NEO 500 Hz. ^{19}F NMR spectra were recorded at Bruker Avance NEO 300 MHz and were referenced to the residual proton signals of $CDCl_3$, 7.26 ppm and acetone-d_6 2.05 ppm. $^{13}C\{^{1}H\}$ NMR were recorded at Bruker Avance NEO 300 MHz (75 MHz) and Bruker Avance NEO 500 Hz (125 MHz) and were referenced to the residual ^{13}C signal of $CDCl_3$, 77.16 ppm and acetone-d_6 29.9 ppm. When required, structural assignments were made with additional information from HSQCed, HMBC, COSY, and NOESY. HRMS were recorded with a mass spectrometer with ESI. Melting points were determined on a LLG-Melting point meter MPM-H2 visual apparatus and are uncorrected. Infrared spectra were recorded on a spectrophotometer equipped with a Golden Gate Single Refraction Diamond ATR (Attenuated Total Reflectance) accessory. High-resolution mass spectra were recorded using electrospray ionization (ESI).

1.3. Materials and methods for photochemical characterization

The optical properties of **PC4**, **PC5** and **PC6** were investigated by UV-vis absorption spectroscopy using an Agilent HP 8453 spectrophotometer. Samples were measured in Hellma Analytics quartz high precision cells with a path length of 10 mm at ambient temperature. Because of the limited solubility of these compounds in aqueous media, UV-vis absorption measurements were conducted in DMSO:H_2O mixtures: 75:25 (**PC4**), 50:50 (**PC5**) and 40:60 (**PC6**) DMSO:H_2O. Their *trans-cis* and *cis-trans* photoisomerization was promoted using different types of irradiation sources: a Hg lamp (λ_{exc} = 365 nm), the third harmonic of a Brilliant ns laser (Quantel, λ_{exc} = 355 nm), light-emitting diodes (LED) with emission maxima at λ_{em} = 365 or 405

nm, and cw diode lasers with λ_{exc} = 405 or 445 nm. ^{1}H NMR measurements were performed to determine the composition of the photostationary states for *trans*-*cis* ($PSS_{t\text{-}c}$) and *cis*-*trans* ($PSS_{c\text{-}t}$) photoisomerization in methanol-*d*$_4$. From this data, the UV-vis absorption spectra of these PSS states in DMSO:H_2O mixtures, and the UV-vis absorption spectra of *trans*-**PC4**, *trans*-**PC5** and *trans*-**PC6** in DMSO:H_2O mixtures, we could estimate: (a) the UV-vis absorption spectra of *cis*-**PC4**, *cis*-**PC5** and *cis*-**PC6** in aqueous media; and (b) the composition of $PSS_{t\text{-}c}$ and $PSS_{c\text{-}t}$ in DMSO:H_2O mixtures. Photoisomerization quantum yields were determined using a previously reported method[14] and 1,2-bis(2-methyl-5-phenyl-3-thienyl)perfluorocyclopentene in hexane as a reference ($\Phi_{ring\ closing}$ = 0.59, $\Phi_{ring\ opening}$ = 0.013[15]).

1.4. Materials and methods for enzyme inhibition assays

The effect of the photocoxibs on the two cyclooxygenase isoenzymes was assessed using COX-1 Inhibitor Screening Kit (Fluorometric) (Abcam, product no. ab204698) and COX-2 Inhibitor Screening Kit (Fluorometric) (Abcam, product no. ab283401), respectively. These assays are based on the variation in the fluorescence intensity of prostaglandin G_2 (PGG_2), the intermediate product generated by COX, in the presence of COX inhibitors. Both kits share the following components: COX Assay Buffer, COX Probe (in DMSO), COX Cofactor (in DMSO), arachidonic acid (AA), and NaOH. The COX-1 kit contains ovine COX-1 enzyme and the reference inhibitor SC-560 (in DMSO), whereas the COX-2 kit contains recombinant human COX-2 and its selective inhibitor celecoxib (in DMSO). The test compounds were dissolved in DMSO at 10× concentration and diluted ten-fold in the assay wells upon addition of the remaining reagents. Reagents and compounds supplied by the kits were prepared as per manufacturer's instructions, and experiments were carried out following the provided protocols.

1.4.1. Experimental conditions. *Blank*: same volume of DMSO used for inhibitors, no enzyme, no inhibitor, all other components added as specified; *enzyme control*: same volume of DMSO used for inhibitors, enzyme present, no inhibitor; *inhibitor control* (SC-560 for COX-1 and celecoxib for COX-2): 10 µL of the reference inhibitor solution supplied with the kit (in DMSO); ***PC4, PC5, PC6***: 10 µL of test compounds (in DMSO; final concentration 45 µM). All wells contained the remaining assay components according to the manufacturer's protocol. Because the test compounds are photoswitchable, each experiment was performed under two independent conditions: dark and UV-light (365 nm). In dark conditions, assays were performed at room temperature without irradiation. In the UV-light condition, after dispensing the test compound solution into the wells, the plate was irradiated for 3 min with a UV lamp (λ = 365 nm) to induce photoisomerization from the *trans* to the *cis* form until the photostationary state $PSS_{t\text{-}c}$ was obtained. Irradiation settings were selected according to the photochemical characterization of the compounds. The Reaction Mix and AA were then added, and fluorescence was monitored

kinetically for 10 min at room temperature (~25 °C) using a BioTek Synergy H1 Multimode Reader (Ex/Em = 535/587 nm). For each condition, two technical replicates were acquired.

1.4.2. Data processing. Matched blank wells (dark and 365 nm) were included in every plate, and blank fluorescence values were subtracted from raw traces for each time point. For every condition, replicate fluorescence traces were averaged point-by-point to produce a mean activity curve, and replicate variability at each time point was expressed as standard deviation (SD). The final relative fluorescent unit (RFU) value at the last recorded time point was used as an endpoint measure of COX activity for calculation of inhibitor efficacy. The percentage inhibition relative to the no-inhibitor control was calculated according to:

$$\% \text{ inhibition} = (1 - \text{RFU\_inhibitor} / \text{RFU\_no-inhibitor}) \times 100 \quad (4)$$

calculated separately for dark and 365 nm conditions. The uncertainty associated with each inhibition value was estimated by propagation of the SD of the final RFU of the inhibitor and the no-inhibitor control. For an inhibitor with endpoint fluorescence x (SD = σ_x) and corresponding control y (SD = σ_y), the propagated error on the inhibition percentage was obtained through the following expression:

$$\sigma\_\text{inhibition} = \sqrt{[\,(100/y \cdot \sigma\_x)^2 + (100 \cdot x/y^2 \cdot \sigma\_y)^2\,]} \quad (5)$$

The resulting percentage inhibition values and propagated errors were plotted as side-by-side bars to allow direct comparison of the dark (trans) and 365 nm ($PSS_{t\text{-}c}$) states for each inhibitor in both COX-1 and COX-2 assays.

1.5. Materials and methods for J774 macrophage cell culture

Irradiation settings were selected according to the photochemical characterisation of the compounds. In particular, *trans*-**PC4-PC6** were dissolved in DMSO (10 mM) and then diluted with Dulbecco's modified Eagles medium without phenol red (DMEM). In the dark condition, these solutions were incubated without irradiation. In the UV-light condition, the solutions were irradiated for 5 min with a UV lamp (λ =365 nm) to induce photoisomerization from the *trans* to the *cis* form until $PSS_{t\text{-}c}$ was obtained before incubation into the wells.

Cell respiration, an indicator of cell viability, was assessed by the mitochondrial-dependent reduction of MTT (Sigma Aldrich, Milan, Italy) to formazan. Cells were plated at a seeding density of 1.0×10^5 in 96 multiwell. After stimulation with *trans*-**PC4-PC6** (dark) and their $PSS_{t\text{-}c}$ mixtures (UV-light) (0.1, 1, 10 and 50 µM) for 24 h, cells were incubated in 96-well plates with MTT (0.2 mg/mL), for 1 h. Culture medium was removed by aspiration, and the cells were lysed in DMSO (0.1 mL). The extent of reduction of MTT to formazan within cells was quantified by the measurement of OD_{550}.[16]

To evaluate the *in vitro* capacity of tested compounds to inhibit the production of prostaglandin E_2 (PGE_2) by COX-1 and COX-2, the murine monocyte/macrophage J774 cell line was used (ATTC TIB 67; American Type Culture Collection). The cell line was grown in adhesion in DMEM without phenol red supplemented with L-glutamine (2 mM, Aurogene Rome, Italy), Hepes (25 mM, Aurogene Rome, Italy), penicillin (100 U/mL, Aurogene Rome, Italy), streptomycin (100 µg/mL, Aurogene Rome, Italy), fetal bovine serum (FBS, 10%, Aurogene Rome, Italy) and sodium pyruvate (1.2%, Aurogene Rome, Italy) (DMEM completed). The cells were plated at a density of 1×10^6 cells in 75 cm^2 culture flasks and maintained at 37°C under 5% CO_2 in a humidified incubator until 90% confluence. The culture medium was changed every 2 days. Before a confluent monolayer appeared, sub-culturing cell process was carried out. To evaluate the effects of *trans*-**PC4**-**PC6** (dark) and their $PSS_{t\text{-}c}$ mixtures (UV-light) on COX-1, cells were pretreated with the tested compounds (10 µM) or indomethacin (Indo, 10 µM) for 15 min and further incubated for 30 min with AA (15 µM). To evaluate the effects on COX-2 cells were treated with photocoxibs (0.1, 1 and 10 µM) or CEL (0.01 µM) and stimulated for 24 h with LPS from *Escherichia coli* (10 µg mL^{-1}), which induces COX-2 expression.[17] At the end of the incubation, the supernatants were collected for the measurement of PGE_2 levels with commercially available ELISA kits according to the manufacturer's instructions (Cayman Chemical, Vinci-Biochem, Vinci, Italy). Triplicate wells were used for the various conditions of the treatment in the cell culture assay throughout the experiments. Results are expressed as the mean ± e.s. of PGE_2 (pg mL^{-1}) or as the mean of three experiments of the % inhibition of PGE_2 production by test compounds with respect to control samples (Table 1). Data fit was obtained using the sigmoidal dose-response equation (variable slope) (GraphPad software). The IC_{50} values were calculated by the GraphPad Instat program (GraphPad software). The results were analyzed by one-way ANOVA followed by Bonferroni post hoc tests. Post hoc tests were performed only if achieved the $p < 0.05$ level of significance. A p-value less than 0.05 was considered significant. All graphs were generated using GraphPad Prism (version 9).

1.6. Materials and methods for in vivo studies with zebrafish larvae

1.6.1. Zebrafish husbandry. Zebrafish (Danio rerio) were maintained at the University of Milan, Via Fratelli Cervi 93 – 20054 Segrate, Milan, Italy (Aut. Prot. n. 295/2012-A – December 20, 2012). Zebrafish were maintained according to international (EU Directive 2010/63/EU) and national guidelines (Italian decree No 26 of the 4th of March 2014). Embryos were collected by natural spawning, staged according to Kimmel et al,.[18] and raised at 28°C in E3 medium fish water (Instant Ocean, 0,1% Methylene Blue) in Petri dishes, according to established techniques. After 24 h post fertilization (hpf), 0.003% 1-phenyl-2-thiourea (PTU, Sigma-Aldrich, Saint Louis, Missouri, USA) was added to fish water to prevent pigmentation. Embryos were washed,

dechorionated, and anaesthetized with 0.016% tricaine (ethyl 3-aminobenzoate methanesulfonate salt; Sigma-Aldrich) before tailfin amputation.

1.6.2. Tailfin amputation. To induce an acute inflammatory response, a portion of the embryo's tailfin was transected using a scalpel blade (5 mm depth), by slicing immediately posterior to the circulatory loop (avoiding damage to the circulatory loop).[19,20]

1.6.3. Pharmacological treatments. Celecoxib, **PC4**, and **PC6** were dissolved in DMSO to achieve a concentration of 10 mM. Treatments were performed in 24-well plates, with a maximum of 15 larvae/well, following tailfin amputation. All compounds were administered at the final doses of 10 µM, 50 µM, and 100 µM in 1 mL of E3 with PTU 1X. Control larvae were treated with the corresponding volume of DMSO. In the dark condition, *trans*-**PC4** and *trans*-**PC6** were administered without irradiation. In the UV-light condition, these compounds were illuminated with an UV lamp at a wavelength of 365 nm, to induce their conversion to the cis configuration until $PSS_{t\text{-}c}$ mixtures were obtained. Pharmacological treatments were performed for 6-h post amputation (hpa).

1.6.4. Leucocytes migration assay through Sudan black staining. At 6 hpa, embryos were fixed overnight in 4% paraformaldehyde (Sigma-Aldrich) PBS at 4°C, rinsed in PBS, then incubated in Sudan Black (Sigma-Aldrich, Saint-Quentin Fallavier, France) for 1 h, washed in 70% ethanol in water, then rehydrated to PBS + 0.1% Tween 20 (PBT). Single slice images of stained embryos were acquired using a microscope equipped with a digital camera with LAS Leica imaging software (Leica, Wetzlar, Germany). Leucocytes migrated to the wound site were counted manually.

1.6.5. Statistical analysis. Results are presented as mean ± SD. The statistical significance of the data was determined by one-way ANOVA with Tukey post hoc correction using GraphPad Prism (GraphPad Software, San Diego, California USA), with p 0.05 (*), p 0.01 (**), and p 0.001(***) being considered statistically significant values.

2. Computational design of photoswitchable celecoxib analogues

2.1. Binding energy predictions

Table S1. Thermodynamic components of the binding energy for compounds (*cis*-PC/*trans*-PC) obtained from MM/PBSA analysis, showing the total Gibbs binding energy ($\Delta G_{binding}$) as the sum of ΔG’, which includes electrostatic, van der Waals, and solvation energies, and the entropic term (-TΔS). Negative binding energetic values are in green while positive values are in red. Near-zero values are coloured orange. All values are in kcal mol^{-1}.

Photoswitchable coxibs	ΔG’	-TΔS	$\Delta G_{binding}$
cis-**PC1**	-8.1	10.4	**2.3**
trans-**PC1**	-10.0	8.2	**-1.8**
cis-**PC2**	-13.7	8.4	**-5.3**
trans-**PC2**	-9.5	8.7	**-0.7**
cis-**PC3**	-1.4	6.4¡	**5.1**
trans-**PC3**	-9.9	7.9¡	**-2.0**
cis-**PC4**	-11.6	7.8	**-3.8**
trans-**PC4**	-7.7	7.1	**-0.6**
cis-**PC5**	-11.1	9.7	**-1.3**
trans-**PC5**	-10.2	9.8	**-0.4**
cis-**PC6**	-13.6	10.2	**-3.4**
trans-**PC6**	-11.0	10.7	**-0.3**
cis-**PC7**	-6.7	11.6	**4.9**
trans-**PC7**	-11.1	9.7	**-1.4**

2.2. Molecular dynamics simulations: final structures

2.2.1. PC1. The binding mode of *cis*-**PC1** presents the sulfonamide group in the side pocket and the trifluoromethyl group together with the pyrazole ring close to the entrance of the cavity (Figure S1a). The difference with celecoxib is that the tolyl ring is above the region between Tyr385 and Ser530. The *trans*-**PC1** binding mode is more similar to that of CEL because the tolyl ring is also in the main pocket although somewhat deeper (Figure S1b).

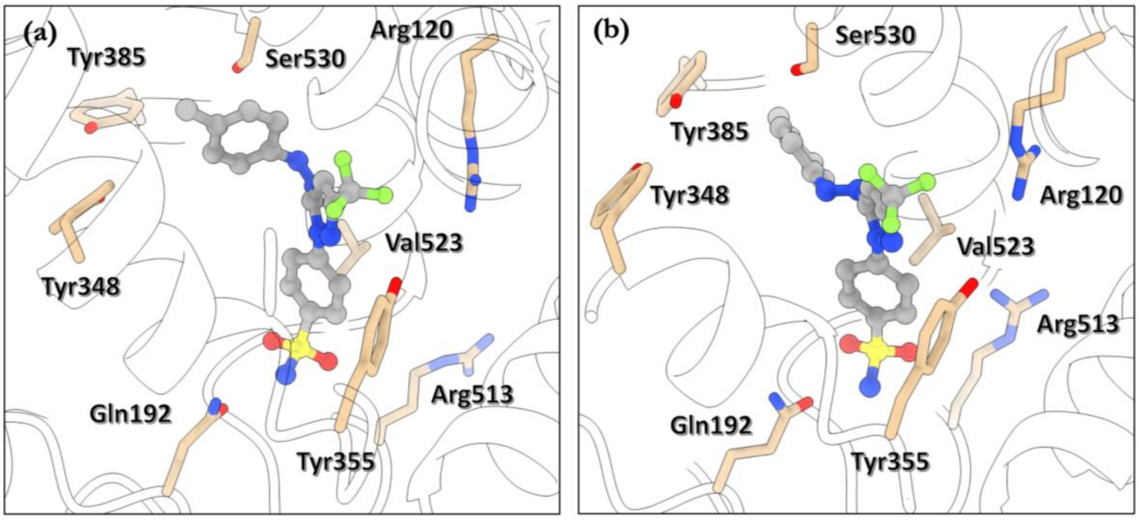

Figure S1. 3D representation of (a) *cis*-**PC1** and (b) *trans*-**PC1** (in grey) bound in the active site of hCOX-2. Each complex structure was derived from the last snapshot of its MD simulation. Key residues within the binding pocket of hCOX-2 are shown in tan, while *cis*-**PC1** and *trans*-**PC1** appear in grey. Nitrogen atoms are depicted in blue, oxygen atoms in red, fluorine atoms in green, and the sulfur atom in yellow. Hydrogen atoms are omitted for clarity.

2.2.2. PC2. The *cis*-**PC2** isomer notably resembles the celecoxib structure and its binding mode into the hCOX-2 cavity, although the pyrazole ring is bound to the region of the catalytic residues instead of pointing to the main pocket (see Figure 2b in the main text). The energetic difference with respect to CEL mainly comes from the enthalpic contribution because the trifluoromethyl substituent attached to the pyrazole ring does not interact as strongly with Arg120 as CEL. In contrast, the *trans*-**PC2** isomer, despite being bound to hCOX-2, presents a binding mode that is not very stable (Figure S2).

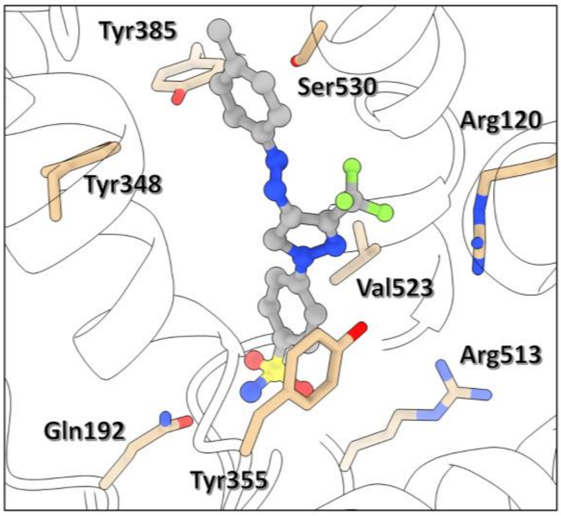

Figure S2. 3D representation of *trans*-**PC2** (in grey) bound in the active site of hCOX-2. This complex structure was derived from the last snapshot of its MD simulation. Key residues within the binding pocket of hCOX-2 are shown in tan, while *trans*-**PC2** appears in grey. Nitrogen atoms are depicted in blue, oxygen atoms in red, fluorine atoms in green, and the sulfur atom in yellow. Hydrogen atoms are omitted for clarity.

2.2.3. PC3 and PC4. The *cis*-**PC3** and *cis*-**PC4** isomers also resemble the celecoxib structure and its binding mode into the hCOX-2 cavity (Figures S3a and S4a). However, as in the *cis*-**PC2** binding mode, the pyrazole ring of *cis*-**PC3** and *cis*-**PC4** is bound to the region of the catalytic residues instead of pointing to the entrance of the main pocket like celecoxib. On the other hand, the strong interaction between the sulfonamide group and the side pocket is maintained similarly to most coxibs. The absence of the trifluoromethyl group in the central pyrazole ring modifies the chemical environment of this part, resulting in enthalpic destabilization of *cis*-**PC3** complex with respect to *cis*-**PC2**, and in a positive Gibbs binding energy. The *trans*-**PC3** isomer forms a more stable complex within the hCOX-2 cavity (Figure S3b) than the *cis* isomer. In contrast, *cis*-**PC4** complex is destabilized by only 1.5 kcal mol^{-1} with respect to *cis*-**PC2** and is more stable than the *trans*-**PC4** isomer complex (Figure S4b).

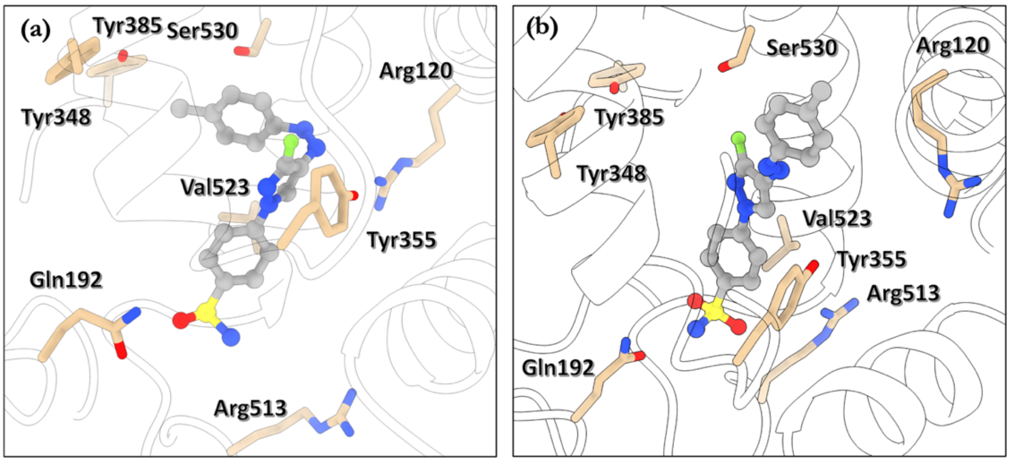

Figure S3. 3D representation of (a) *cis*-**PC3** and (b) *trans*-**PC3** (in grey) bound in the active site of hCOX-2. Each complex structure was derived from the last snapshot of its MD simulation. Key residues within the binding pocket of hCOX-2 are shown in tan, while *cis*-**PC3** and *trans*-**PC3** appear in grey. Nitrogen atoms are depicted in blue, oxygen atoms in red, fluorine atoms in green, and the sulfur atom in yellow. Hydrogen atoms are omitted for clarity.

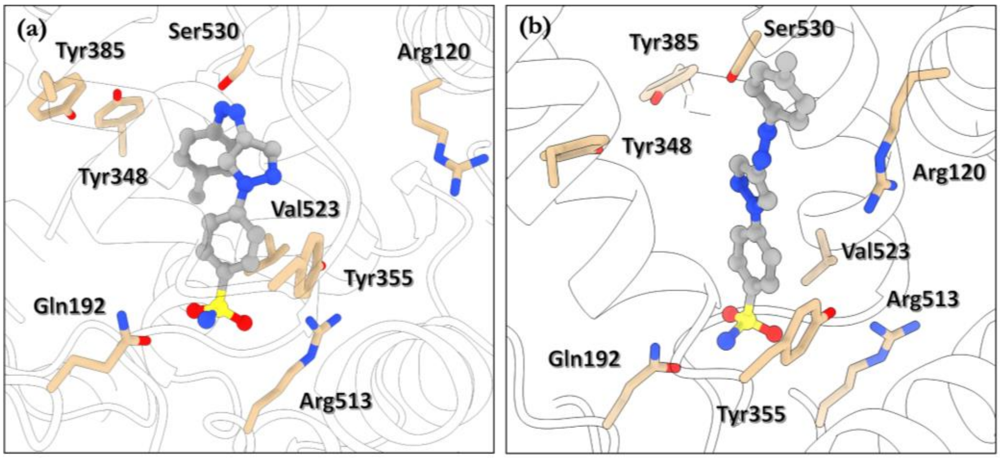

Figure S4. 3D representation of (a) *cis*-**PC4** and (b) *trans*-**PC4** (in grey) bound in the active site of hCOX-2. Each complex structure was derived from the last snapshot of its MD simulation. Key residues within the binding pocket of hCOX-2 are shown in tan, while *cis*-**PC4** and *trans*-

PC4 appear in grey. Nitrogen atoms are depicted in blue, oxygen atoms in red, fluorine atoms in green, and the sulfur atom in yellow. Hydrogen atoms are omitted for clarity.

2.2.4. PC5. The strong interaction between the *cis*-**PC5** sulfonamide group and the side pocket of hCOX-2, as well as the location of the trifluoromethyl group at the top region of the main pocket in the cavity, is maintained compared to celecoxib (Figure S5a). However, the increased length (with an ethyl terminal substituent) and the disposition of the azo group in the main pocket introduce energy and entropy penalties, reducing the overall binding affinity of this candidate compared with *cis*-**PC2** and *cis*-**PC4**. *trans*-**PC5** binds in the cavity (Figure S5b), but with two main differences: the inversion of the trifluoromethyl group and the location of the terminal phenyl ring at the top of the main pocket.

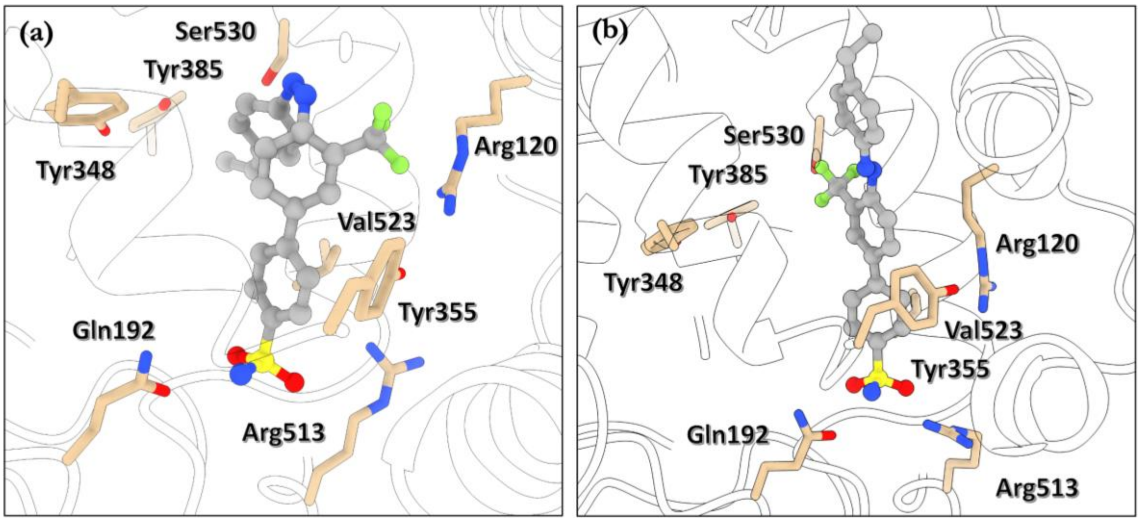

Figure S5. 3D representation of (a) *cis*-**PC5** and (b) *trans*-**PC5** (in grey) bound in the active site of hCOX-2. Each complex structure was derived from the last snapshot of its MD simulation. Key residues within the binding pocket of hCOX-2 are shown in tan, while *cis*-**PC5** and *trans*-**PC5** appear in grey. Nitrogen atoms are depicted in blue, oxygen atoms in red, fluorine atoms in green, and the sulfur atom in yellow. Hydrogen atoms are omitted for clarity.

2.2.5. PC6. The main difference between *cis*-**PC5** and *cis*-**PC6** lies in the terminal substituent on the aromatic ring, where **PC6** features a methoxy group. Figure S6a shows how the location of the terminal aromatic ring and the trifluoromethyl group varied upon complexation with hCOX-2. These two parts of the molecule exchanged positions compared with the binding mode of *cis*-**PC5**. This new situation makes the candidate binding mode less celecoxib-like despite its favourable binding energy. The *trans*-**PC6** orientation inside the catalytic cavity resembles that of *trans*-**PC2** and *trans*-**PC4,** the three isomers with binding energies around zero kcal mol^{-1} (Figure S6b).

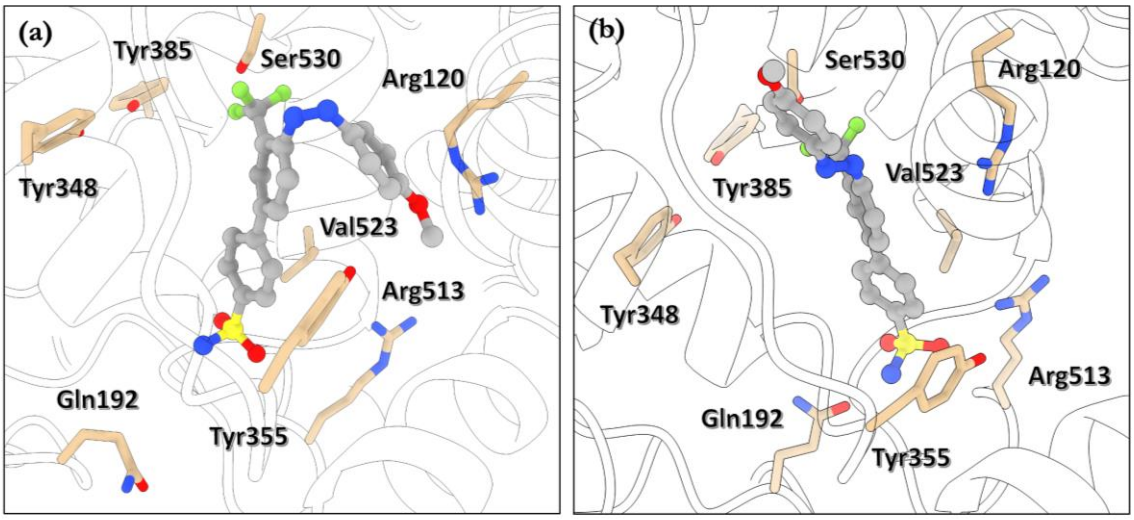

Figure S6. 3D representation of (a) *cis*-**PC6** and (b) *trans*-**PC6** (in grey) bound in the active site of hCOX-2. Each complex structure was derived from the last snapshot of its MD simulation. Key residues within the binding pocket of hCOX-2 are shown in tan, while *cis*-**PC6** and *trans*-**PC6** appear in grey. Nitrogen atoms are depicted in blue, oxygen atoms in red, fluorine atoms in green, and the sulfur atom in yellow. Hydrogen atoms are omitted for clarity.

2.2.6. PC7. *cis*-**PC7** shows a binding mode more similar to *cis*-**PC5** (Figure S7a), but its binding Gibbs energy increases because the trifluoromethyl group is not so close to Arg120. In the same way, the binding mode of *trans*-**PC7** is more similar to *trans*-**PC5** (Figure S7b).

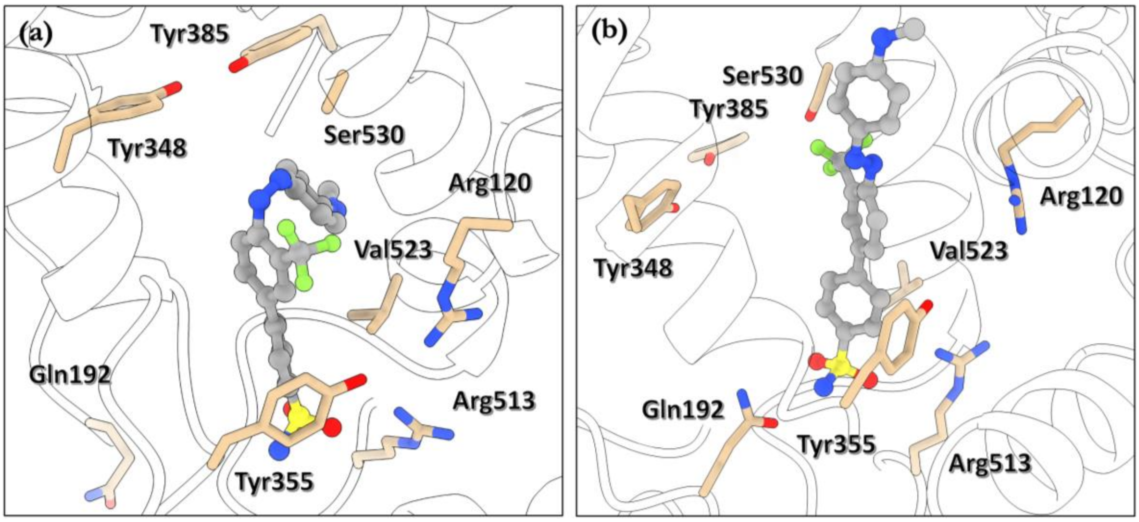

Figure S7. 3D representation of (a) *cis*-**PC7** and (b) *trans*-**PC7** (in grey) bound in the active site of hCOX-2. Each complex structure was derived from the last snapshot of its MD simulation. Key residues within the binding pocket of hCOX-2 are shown in tan, while *cis*-**PC7** and *trans*-**PC7** appear in grey. Nitrogen atoms are depicted in blue, oxygen atoms in red, fluorine atoms in green, and the sulfur atom in yellow. Hydrogen atoms are omitted for clarity.

2.3. Analysis of distances and RMSDs for MD simulations of the best candidates

(a)

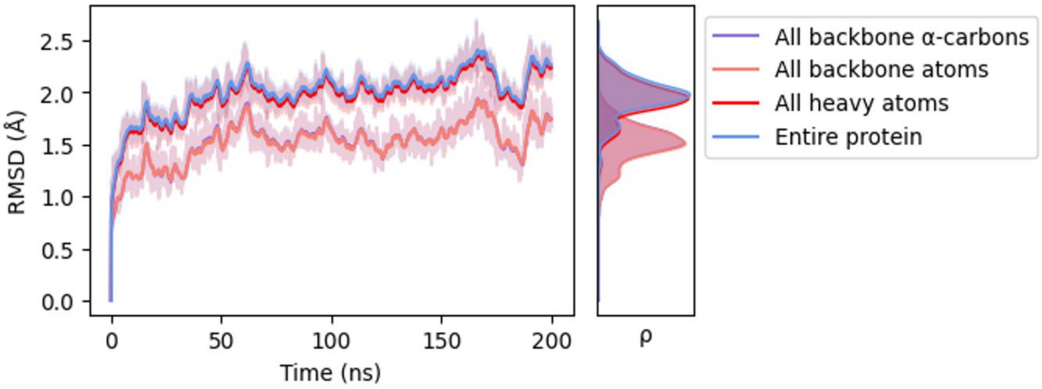

(b)

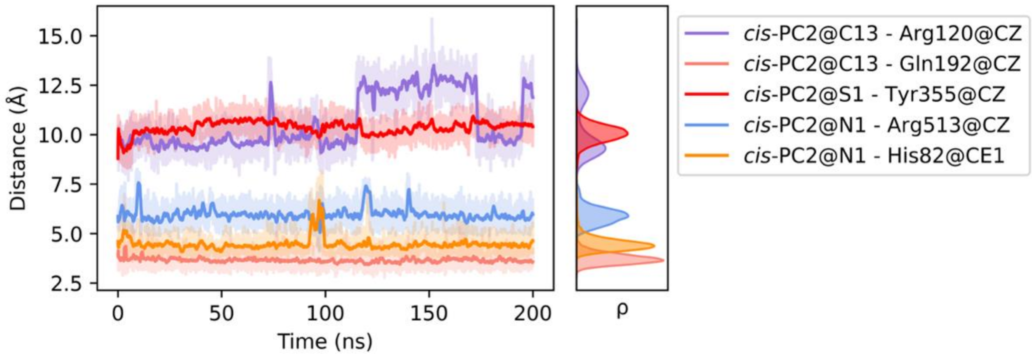

(c)

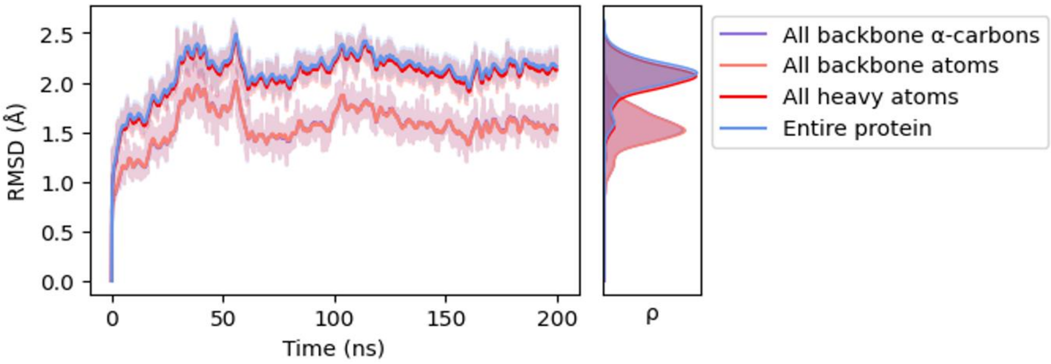

(d)

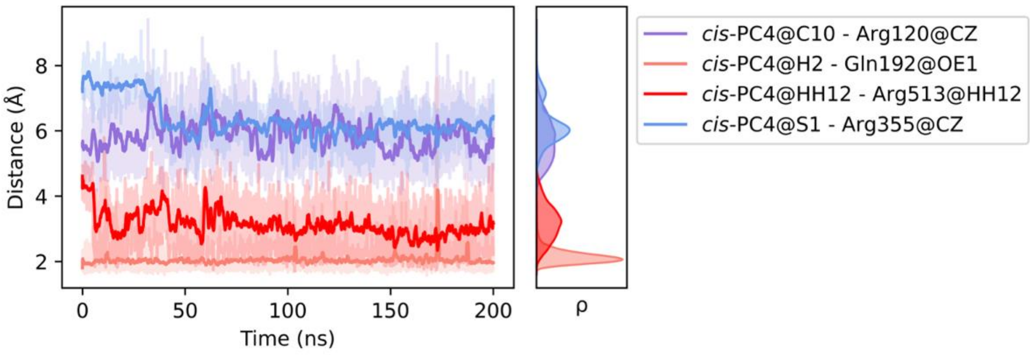

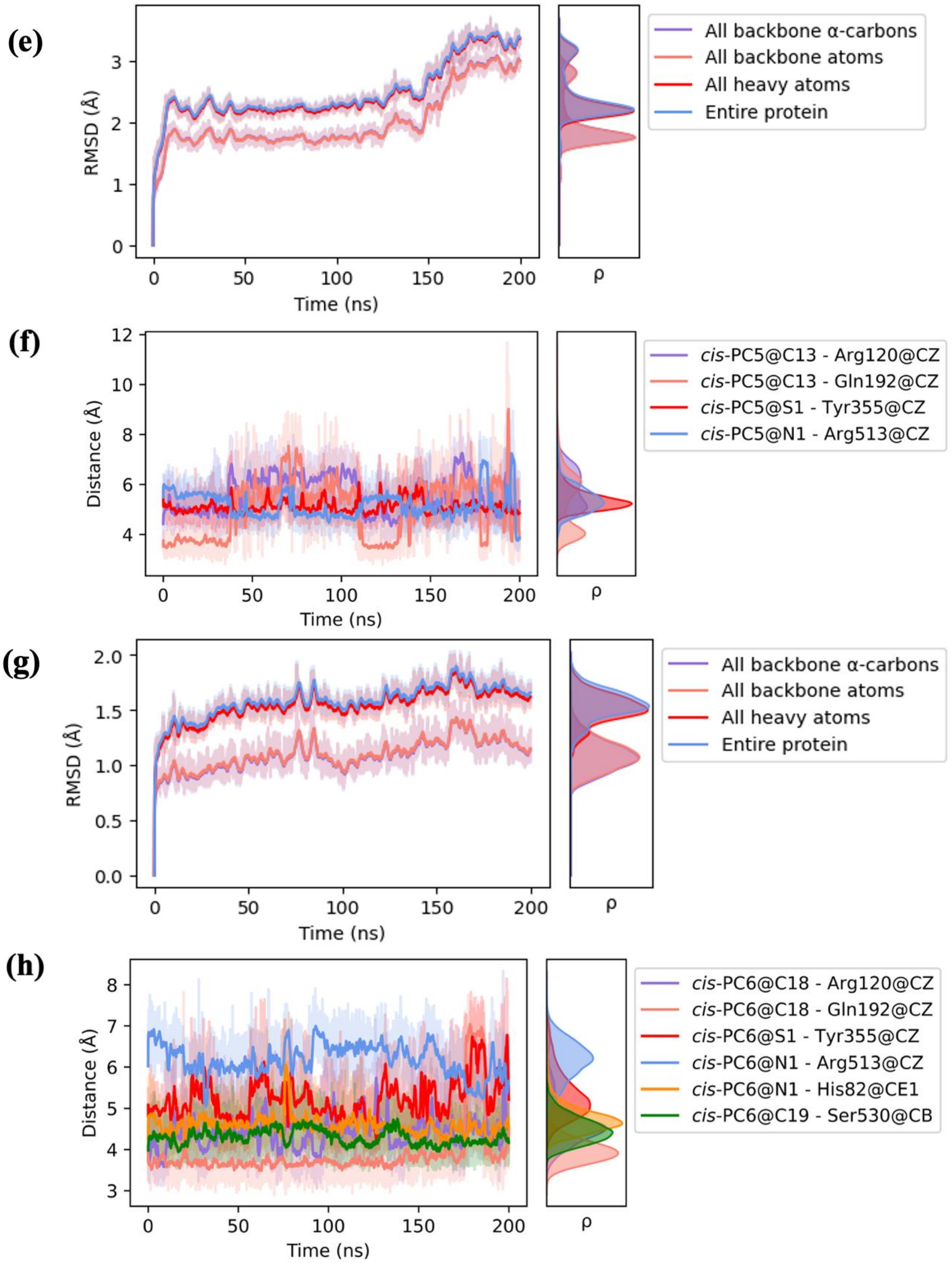

Figure S8. Time evolution of RMSD values for the azoderivatives/hCOX-2 complexes and specific residue-ligand distances over 200 ns MD simulations. Panels (a), (c), (e) and (g) show RMSD values for α-carbons, backbone atoms, heavy atoms, and the entire protein, respectively. Panels (b), (d), (f) and (h) display distance values for key residues in the binding pocket including Arg120, Gln192, Tyr355, Arg513, Ser530, and His82 with the four candidates. Panels (a, b) refer to *cis*-**PC2**, (c, d) to *cis*-**PC4**, (e, f) to *cis*-**PC5**, and (g, h) to *cis*-**PC6**. Solid lines represent mean values with shaded regions indicating real values. Density plots on the right show the distribution of values for each metric.

3. Synthesis of photoswitchable celecoxib analogs

3.1. Synthesis of PC2

*4-nitro-3-(trifluoromethyl)-1H-pyrazole, **10a***[21]

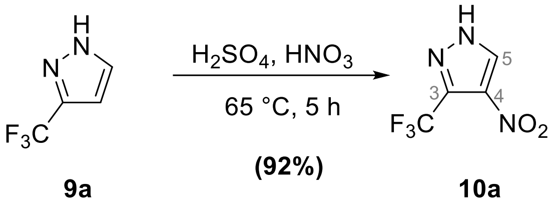

Compound **9a** (116 mg, 0.85 mmol) was added portionwise to 98% H_2SO_4 (0.5 mL) at 25 °C. After that, 60% HNO_3 (81 µL) was added dropwise at 25 °C, and the solution was heated at 65 °C for 5 h. Afterwards, the reaction mixture was cooled to -5 °C using an ice-acetone bath and neutralized with a 2M NaOH solution (9.40 mL). The product was extracted with EtOAc (3 x 20 mL). The combined organic extracts were dried over anhydrous Na_2SO_4, filtered, and concentrated under vacuum, affording a white solid identify as product **10a** (141 mg, 0.78 mmol, 92% yield). ^{1}H NMR (300 MHz, $CDCl_3$) δ 11.1 (br s, 1H, -NH), 8.45 (s, 1H, H-5).

*3-(trifluoromethyl)-1H-pyrazol-4-amine, **1a***[22]

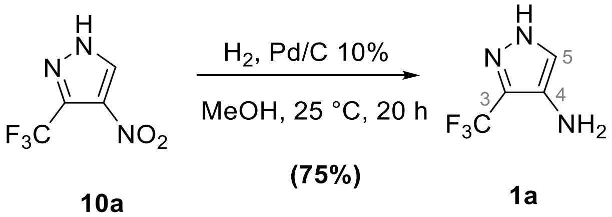

To a solution of compound **10a** (526 mg, 2.90 mmol) in MeOH (25 mL), 10% palladium on charcoal (74.6 mg) was added. The reaction mixture was stirred at 25 °C for 24 h under a hydrogen atmosphere. The reaction mixture was then filtered through a Celite plug under argon, and the pad was washed with EtOAc (100 mL) previously degassed with argon. The combined filtrate was concentrated under vacuum to furnish a colorless liquid identified as compound **1a** (329 mg, 2.17 mmol, 75% yield), which should be stored under an inert atmosphere due to its rapid oxidation in air. ^{1}H NMR (300 MHz, $CDCl_3$) δ 11.96 (br s, 1H, -NH), 7.21 (s, 1H, H-5), 3.28 (br s, 2H, -NH_2).

*4-(4-nitro-3-(trifluoromethyl)-pyrazol-1-yl)benzenesulfonamide, **11***

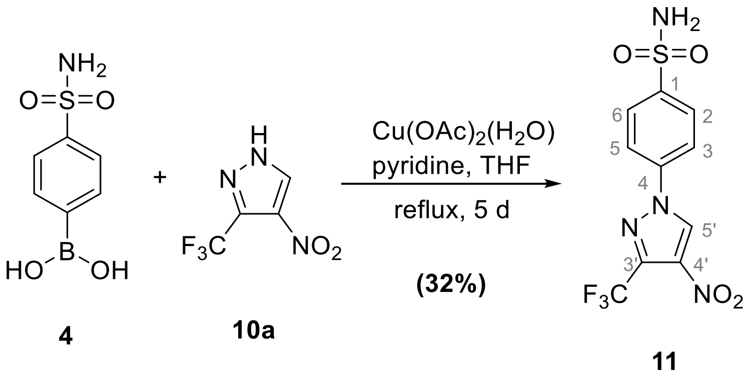

To a stirred solution of compound **10a** (186 mg, 1.03 mmol) in THF (12 mL), copper (II) acetate hydrate (15 mg, 7% mol), pyridine (149 µL, 1.85 mmol) and 4-sulfamoylbenzeneboronic acid, **4** (706 mg, 3.51 mmol, 4.4 equiv.) were added. The reaction was heated under reflux in open air for 5 days, and its progress was monitored by NMR analysis. After this period, the reaction mixture was filtered, and the resulting solution was basified with a saturated aqueous solution of K_2CO_3 (20 mL). The product was extracted with EtOAc (3 x 25 mL). The combined organic layers were dried over anhydrous Na_2SO_4, filtered and concentrated under vacuum. The resulting crude was purified by recrystallization from hot HPLC-grade DCM to furnished product **13** (121 mg, 0.33 mmol, 32% yield) as a white solid. Mp:108-110 °C (from DCM); ^{1}H NMR (500 MHz, acetone-d_6) δ 9.66 (s, 1H, H-5'), 8.23 (d, $J_{3/5,2/6}$= 8.5 Hz, 2H, H-3, H-5), 8.14 (d, $J_{2/6,3/5}$= 8.5 Hz, 2H, H-2, H-6), 6.82 (br s, 2H, -SO_2NH_2); ^{13}C{^{1}H} NMR (125 MHz, acetone-d_6) δ 145.6 (C-4), 141.3 (C-1), 137.6 (q, $J_{3',F}$= 39.6 Hz, C-3'), 135.1 (C-4'), 132.6 (C-5'), 128.8 (C-3, C-5), 121.5 (C-2, C-6), 120.5 (q, $J_{CF3,F}$= 269.6 Hz, -CF_3); IR (ATR, cm^{-1}) 3550, 3358, 3268, 2924, 2854, 1595, 1558, 1496. HSQCed and HMBC experiments were recorded.

3.2. Synthesis of PC4

*4-nitro-1H-pyrazole, **13***[23]

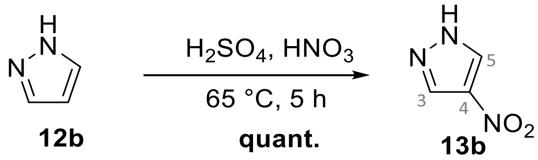

Pyrazole, **12b** (505 mg, 7.42 mmol), was added portionwise to 98% H_2SO_4 (4.0 mL) at 25 °C. Then, 60% HNO_3 (0.7 mL) was added dropwise at 25 °C. The reaction was heated to 65 °C for 5 h. Afterwards, the reaction mixture was cooled to -5 °C using an ice-acetone bath and neutralized with a 2M NaOH (75.6 mL) solution. The product was extracted with EtOAc (3 x 100 mL). The combined organic extracts were dried over anhydrous Na_2SO_4, filtered, and concentrated under vacuum affording a white solid identify as product **13b** (839 mg, 7.42 mmol, 100% yield). ^{1}H NMR (300 MHz, $CDCl_3$) δ 10.4 (br s, 1H, -NH), 8.27 (s, 2H, H-3, H-5).

*Synthesis of 4-amino-1H-pyrazol, **1b***[23,24]

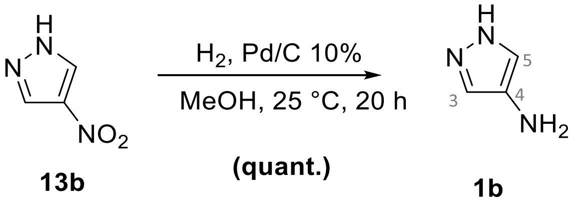

To a solution of compound **13b** (612 mg, 5.27 mmol) in MeOH (47 mL), 10% palladium on charcoal (143 mg) was added. The reaction mixture was stirred at 25 °C for 24 h under a hydrogen atmosphere. The reaction mixture was filtered through a Celite plug, which was washed several times with MeOH (150 mL). The obtained solution was concentrated under vacuum to furnish compound **1b** (450 mg, 5.27 mmol, 100% yield) as a solid. The sample was stored under an argon atmosphere to prevent oxidation. ^{1}H NMR (300 MHz, $CDCl_3$) δ 7.23 (s, 2H, H-3, H-5), 2.95 (br s, 2H, $-NH_2$).

*(E)-4-(p-tolyldiazenyl)-1H-pyrazole, trans-**3b***

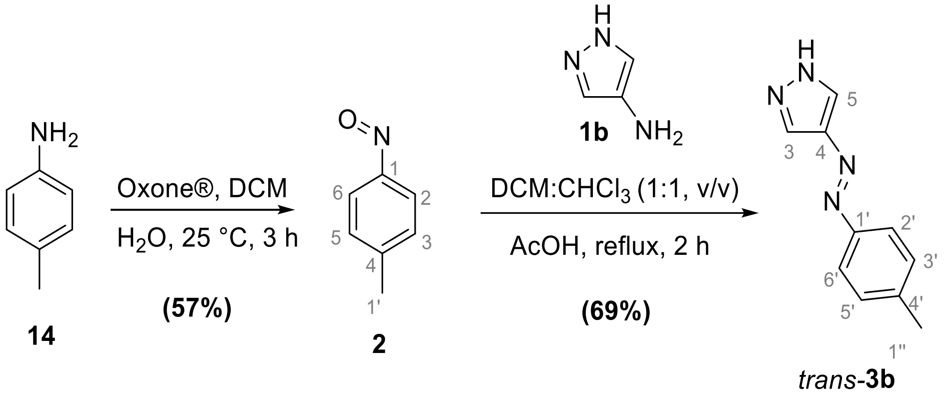

To a solution of *p*-toluidine, **14** (899 mg, 8.39 mmol) in DCM (17 mL), a solution of Oxone® (5.20 g, 16.9 mmol) in H_2O (16 mL) was added under an argon atmosphere. The reaction mixture was stirred at 25 °C for 3 h, with its conversion monitored by NMR analysis. Once the reaction was finished, the DCM layer was separated, and the aqueous layer was washed with DCM (3 x 25 mL). The combined organic layers were then washed with 1M HCl (50 mL), a saturated solution of $NaHCO_3$ (50 mL) and brine (50 mL). The organic layer was dried over anhydrous Na_2SO_4, filtered and concentrated under vacuum. The resulting green liquid was identified as a (1:3) mixture of amine **14** and nitroso **2** (987 mg, by NMR analysis).[25] **2**: ^{1}H NMR (400 MHz, $CDCl_3$) δ 7.79 (d, $J_{2/6,3/5}$= 7.7 Hz, 2H, H-2, H-6), 7.38 (d, $J_{3/5,2/6}$= 7.8 Hz, 2H, H-3, H-5), 2.43 (s, 3H, H-1').

To a stirred solution of compound **1b** (449 mg, 5.40 mmol) in 1:1 (v/v) mixture of DCM and $CHCl_3$ (24 mL), the above (1:3) mixture of compounds **14** and **2** (987 mg) in AcOH (2 mL) was

added under an argon atmosphere. The reaction mixture was stirred under reflux for 2 h, and its progress was monitored by TLC (hexane/EtOAc, 1:1). After completion, the solvent was removed under reduced pressure. The residue was purified by flash column chromatography (hexane/EtOAc, 1:1) to provide *trans*-**3b** (608 mg, 3.27 mmol, 61%) as a bright yellow solid. Mp : 211-214 °C (from a mixture of hexane and EtOAc); ^{1}H NMR (300 MHz, $CDCl_3$) δ 10.3 (br s, 1H, -NH), 8.16 (s, 2H, H-3, H-5), 7.73 (d, $J_{2'/6', 3'/5'}$= 8.3 Hz, 2H, H-2', H-6'), 7.29 (d, $J_{2'/6', 3'/5'}$= 8.4 Hz, 2H, H-3', H-5'), 2.42 (s, 3H, H-1"); ^{13}C{^{1}H} NMR (125 MHz, $CDCl_3$) δ 151.1 (C-4'), 141.1 (C-1', C-4), 129.9 (C-3', C-5', C-5, C-3), 122.42 (C-2', C-6'), 21.6 (C-1"); IR (ATR, cm^{-1}) 3147, 3086, 3021, 2955, 2912, 2853, 1499. HRMS (Q-TOF) calcd. for $[C_{10}H_{10}N_4+H]^+$: 187.0978; found: 187.0981. HMBC experiment was recorded.

*(E)-4-(4-(p-tolyldiazenyl)-1H-pyrazol-1-yl)benzenesulfonamide, trans-**PC4***

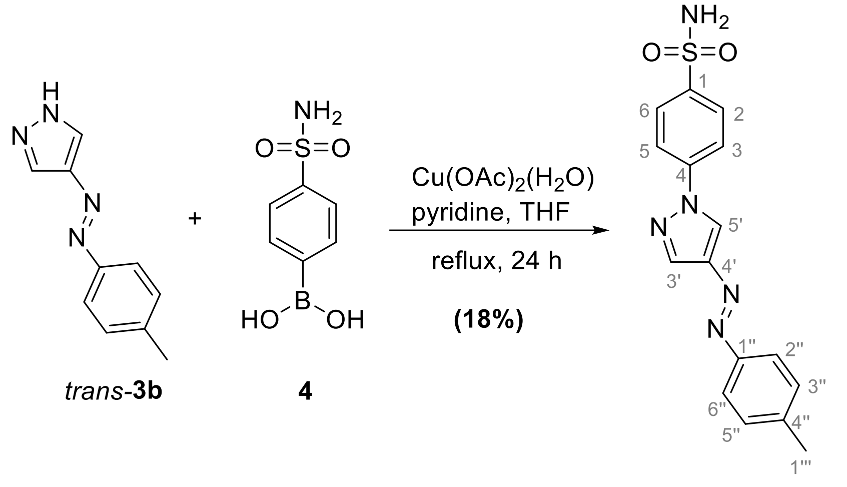

To a stirred solution of compound *trans*-**3** (214 mg, 1.15 mmol) in THF (19 mL), copper (II) acetate hydrate (110 mg, 0.55 mmol), pyridine (339 µL, 4.20 mmol) and boronic acid **4** (318 mg, 1.58 mmol) were added. The reaction mixture was heated under reflux for 24 h, and its progress was monitored by NMR analysis. The solvent was evaporated under reduced pressure, and the resulting residue was redissolved in EtOAc (20 mL). The organic layer was washed with an aqueous solution of Na_2S 0.20 M (10 mL), producing CuS as a black solid which was separated by filtration. The organic layer was dried over anhydrous Na_2SO_4 and concentrated under vacuum. The residue was purified by recrystallization from hot HPLC-grade DCM to give a bright yellow powder identified as *trans*-**PC4** (68.7 mg, 0.20 mmol, 18% yield). Mp: 261-264 °C (from DCM); ^{1}H NMR (300 MHz, acetone-d_6) δ 9.14 (s, 1H, H-5'), 8.25 (s, 1H, H-3'), 8.18 (d, $J_{3/5,2/6}$= 6.6 Hz, 2H, H-3, H-5) 8.08 (d, $J_{2/6,3/5}$= 6.3 Hz, 2H, H-2, H-6), 7.76 (d, $J_{2''/6'',3''/5''}$= 6.6 Hz, 2H, H-2", H-6"), 7.39 (d, $J_{3''/5'',2''/6''}$= 6.9 Hz, 2H, H-3", H-5"), 6.70 (br s, 2H, $-SO_2NH_2$), 2.42 (s, 3H, H-1"'); ^{13}C{^{1}H} NMR (125 MHz, acetone-d_6) δ 151.9 (C-4"), 144.3 (C-4'), 143.4 (C-4), 142.9 (C-1),

142.3 (C-1''), 135.0 (C-3'), 130.7 (C-3'', C-5''), 128.7 (C-2, C-6), 126.3 (C-5'), 123.2 (C-2'', C-6''), 119.8 (C-3, C-5), 21.4 (C-1'''); IR (ATR, cm^{-1}): 3399, 3268, 3111, 2550, 2411, 1594, 1545, 1503. HRMS (Q-TOF) calcd. for $[C_{16}H_{15}N_5O_2S+H]^+$: 342.1019; found: 342.1022. HSQCed, HMBC and NOESY experiments were recorded.

3.3 Synthesis of PC5

*4'-amino-3'-(trifluoromethyl)-[1,1'-biphenyl]-4-sulfonamide, **6***

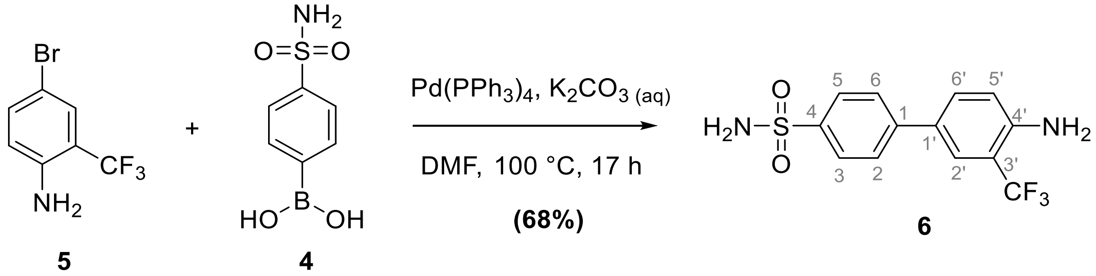

To a stirred solution of amine **5** (812 mg, 3.38 mmol) in DMF (6 mL), a solution of boronic acid **4** (782 mg, 3.90 mmol), tetrakis(triphenylphosphine)palladium(0) (217 mg) and an aqueous solution of 2M K_2CO_3 (5.6 mL) were added under an argon atmosphere. The reaction was stirred and heated to 100 °C for 17 h. After cooling to 25 °C, the mixture was poured into a half-saturated aqueous solution of $NaHCO_3$ (12 mL). The product was extracted with EtOAc (3 x 20 mL). The whole combined organic layers were washed with brine (5 x 12 mL), dried over with anhydrous Na_2SO_4, filtered and concentrated under vacuum. The residue was purified by flash column chromatography (hexane/EtOAc, 1:1) to provide the desired product **6** (728 mg, 2.30 mmol, 68% yield) as a white solid. Mp: 240-241 °C (from a mixture of hexane and EtOAc); ^{1}H NMR (300 MHz, acetone-d_6) δ 7.93 (dt, $J_{2/6,3/5}$ = 2.6 Hz, $J_{2/6,5/3}$ = 8.7 Hz, 2H, H-2, H-6), 7.80 (dt, $J_{3/5,2/6}$ = 2.0 Hz, $J_{5/3,6/2}$ = 8.7 Hz, 2H, H-3, H-5), 7.76 (d, $J_{2',6'}$ = 2.2 Hz, 1H, H-2'), 7.72 (dd, $J_{6',2'}$ = 2.2 Hz, $J_{6',5'}$ = 7.7 Hz, 1H, H-6'), 7.06 (d, $J_{5',6'}$ = 8.5 Hz, 1H, H-5'), 6.57 (br s, 2H, $-SO_2NH_2$), 5.44 (br s, 2H, $-NH_2$); ^{13}C{^{1}H} NMR (75 MHz, acetone-d_6) δ 147.2 (q, $J_{4',F}$ = 1.9 Hz, C-4'), 144.2 (C-4), 143.1 (C-1), 132.6 (q, $J_{6',F}$ = 1.2 Hz, C-6'), 128.0 (C-1'), 127.6 (C-2, C-6), 127.0 (C-3, C-5), 126.2 (q, $J_{7',F}$ = 271.3 Hz, $-CF_3$), 125.6 (q, $J_{2',F}$ = 5.2 Hz, C-2'), 118.6 (C-5'), 113.2 (q, $J_{3',F}$ = 30.3 Hz, C-3'); IR (ATR, cm^{-1}) 3486, 3397, 3256, 2923, 1646, 1579. HRMS (Q-TOF) calcd. for $[C_{13}H_{11}F_3N_2O_2S+H]^+$: 317.0566; found: 317.0570. HSQCed and HMBC experiments were recorded.

*(E)-4'-((4-ethylphenyl)diazenyl)-3'-(trifluoromethyl)-[1,1'-biphenyl]-4-sulfonami-de, trans-**PC5***

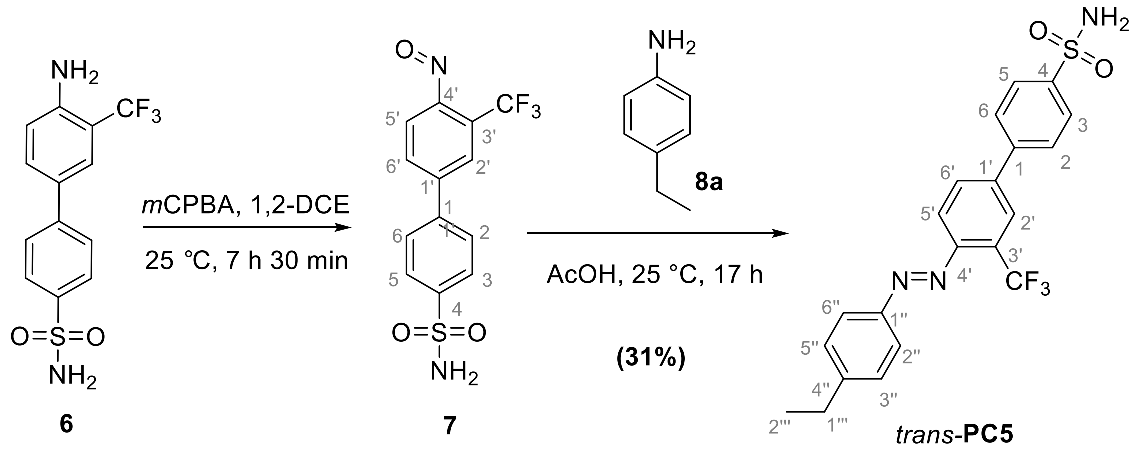

To a solution of amine **6** (66 mg, 0,209 mmol) in 1,2-DCE (8 mL), a solution of *m*CPBA <77% (120 mg, 0.698 mmol) in DMF (1.6 mL) was added under an argon atmosphere. The reaction mixture was stirred at 25 °C for 7 h and 30 min, and its progress was monitored by NMR analysis. After this period, DCM (3 mL) was added to dilute the reaction mixture. The mixture was then washed with a saturated aqueous solution of $Na_2S_2O_3$ (10 mL) and a saturated aqueous solution of $NaHCO_3$ (10 mL). The resulting organic layer was dried over anhydrous Na_2SO_4, filtered and concentrated under vacuum. The resulting green liquid was identified as a (3:7) mixture of amine **6** and nitroso **7** (68.2 mg, by NMR analysis), which was used in the following step without further purification. **7**: ^{1}H NMR (300 MHz, acetone-*d*$_6$): δ 8.52 (s, 1H, H-2'), 8.15 (d, $J_{6',5'}$ = 8.3Hz, 1H, H-6'), 8.06 (s, 4H, H-2, H-3, H-5, H-6), 6.78 (br s, 2H, -SO_2NH_2), 6.48 (d, $J_{5',6'}$ = 8.4 Hz, 1H, H-5').

To a solution mixture of compounds **6** and **7** (68.2 mg) in AcOH (8 mL), a solution of amine **8a** (27.6 mg, 0.228 mmol) in AcOH (16.5 mL) was added under an argon atmosphere. The reaction was stirred overnight preserved from light. Then, toluene (6 mL) was added, and the resulting mixture was evaporated under pressure. The residue was purified by flash column chromatography (hexane/EtOAc, 3:2) to afford the desired product *trans*-**PC5** (28.3 mg, 0.0653 mmol, 31% yield) as an orange solid. Mp: 209-212 °C (from a mixture of hexane and EtOAc); ^{1}H NMR (300 MHz, acetone-*d*$_6$) δ 8.25 (d, $J_{2',6'}$ = 1.8 Hz, 1H, H-2'), 8.18 (dd, $J_{6',5'}$ = 8.5 Hz, $J_{6',2'}$ = 2.1 Hz, 1H, H-6'), 8.05 (m, 5H, H-2, H-3, H-5, H-6, H-5'), 7.94 (dt, $J_{2''/6'', 3''/5''}$ = 8.6 Hz, $J_{2'',6''}$ = 2.0 Hz, 2H, H-2'', H-6''), 7.49 (dt, $J_{3''/5'',2''/6''}$ = 8.2 Hz, $J_{3',5'}$ = 2.1 Hz, 2H, H-3'', H-5''), 6.73 (br s, 2H, -SO_2NH_2), 2.77 (q, $J_{1''',2'''}$ = 7.6 Hz, 2H, H-1'''), 1.28 (t, $J_{2''',1'''}$ = 7.6 Hz, 3H, H-2'''); ^{113}C{^{1}H} NMR (75 MHz, acetone-*d*$_6$) δ 152.0 (C-1''), 150.4 (C-4''), 149.8 (C-4'), 145.1 (C-4), 142.9 (C-1), 142.6 (C-1'), 132.8 (C-6'), 129.8 (C-3'', C-5''), 129.3 (q, $J_{2',F}$ = 30.9 Hz, C-3'), 128.6 (C-2, C-6), 127.8 (C-3, C-5), 126.2 (q, $J_{3',F}$ = 5.52 Hz, C-2'), 125.1 (q, $J_{CF3,F}$ = 273.5 Hz, -CF_3), 124.4 (C-2'', C-6''), 118.0 (C-5'), 29.4 (C-1'''), 15.8 (C-2'''); IR (ATR, cm^{-1}) 3342, 3261, 2970, 1313, 1260, 1154. HRMS (Q-TOF) calcd. for $[C_{21}H_{18}F_3N_3O_2S+H]^+$: 434.1145; found: 434.1151. COSY, HSQCed and HMBC experiments were recorded.

3.4 Synthesis of PC6

*(E)-4'-((4-methoxyphenyl)diazenyl)-3'-(trifluoromethyl)-[1,1'-biphenyl]-4-sulfonamide, trans-**PC6***

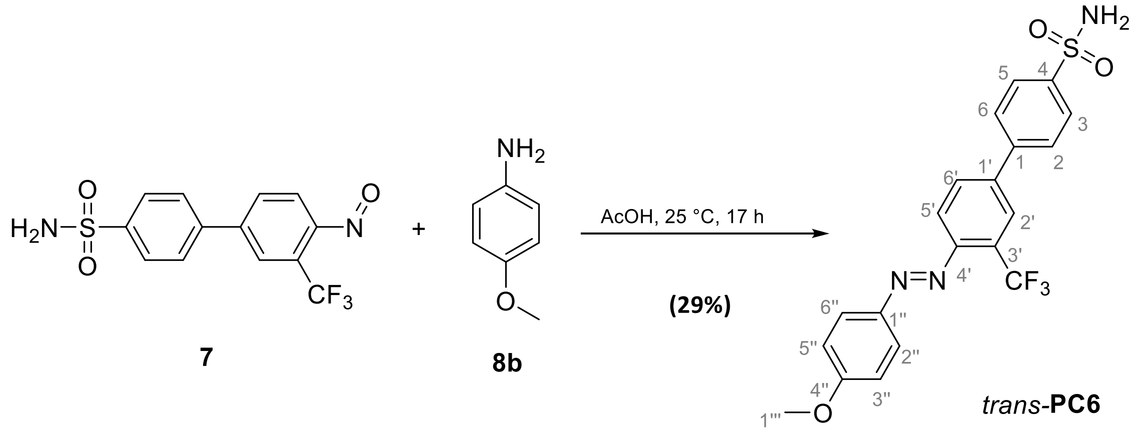

To a (3:7) mixture of compounds **6** and **7** (155 mg, as described above) in AcOH (5 mL), a solution of amine **8b** (60.7 mg, 0.493 mmol) in AcOH (16.5 mL) was added under an argon atmosphere. The reaction was stirred overnight protected from light. Then, toluene (5 mL) was added, and the solvent was evaporated under reduced pressure. The final crude was purified by flash column chromatography (hexane/EtOAc, 3:2) to provide the desired product *trans*-**PC6** (60.0 mg, 0.14 mmol, 29% yield) as an orange solid. Mp: 203-206 °C (from a mixture of hexane and EtOAc); ^{1}H NMR (300 MHz, acetone-d_6) δ 8.23 (s, 1H, H-2'), 8.16 (d, $J_{6',5'}$ = 8.4 Hz, 1H, H-6'), 8.04 (m, 7H, H-2", H-6", H-5', H-2, H-3, H-5, H-6), 7.17 (d, $J_{3''/5'',2''/6''}$ = 7.7 Hz, 2H, H-3", H-5"), 6.71 (br s, 2H, -SO_2NH_2), 3.95 (s, 3H, H-1'"); ^{13}C{^{1}H} NMR (75 MHz, acetone-d_6) δ 164.3 (C-4"), 150.0 (C-4'), 148.0 (C-1"), 145.0 (C-4), 142.9 (C-1), 142.1 (C-1'), 132.7 (C-6'), 129.1 (q, $J_{3',F}$ = 31.0 Hz, C-3'), 128.6 (C-2, C-6), 127.8 (C-3, C-5), 126.4 (C-3", C-5"), 126.1 (q, $J_{2',F}$= 5.8 Hz, C-2'), 124.9 (q, $J_{CF3,F}$ = 274.0 Hz, -CF_3),117.9 (C-5'), 115.5 (C-2", C-6"), 56.2 (C-1'"); IR (ATR, cm^{-1}) 3370, 3268, 3004, 2935, 2834, 1603, 1585, 1500. HRMS (Q-TOF) calcd. for $[C_{20}H_{16}F_3N_3O_3S+H]^+$: 436.0937; found: 436.0942. COSY, HSQCed and HMBC were recorded.

4. <u>Photochemical characterization of PC4, PC5 and PC6</u>

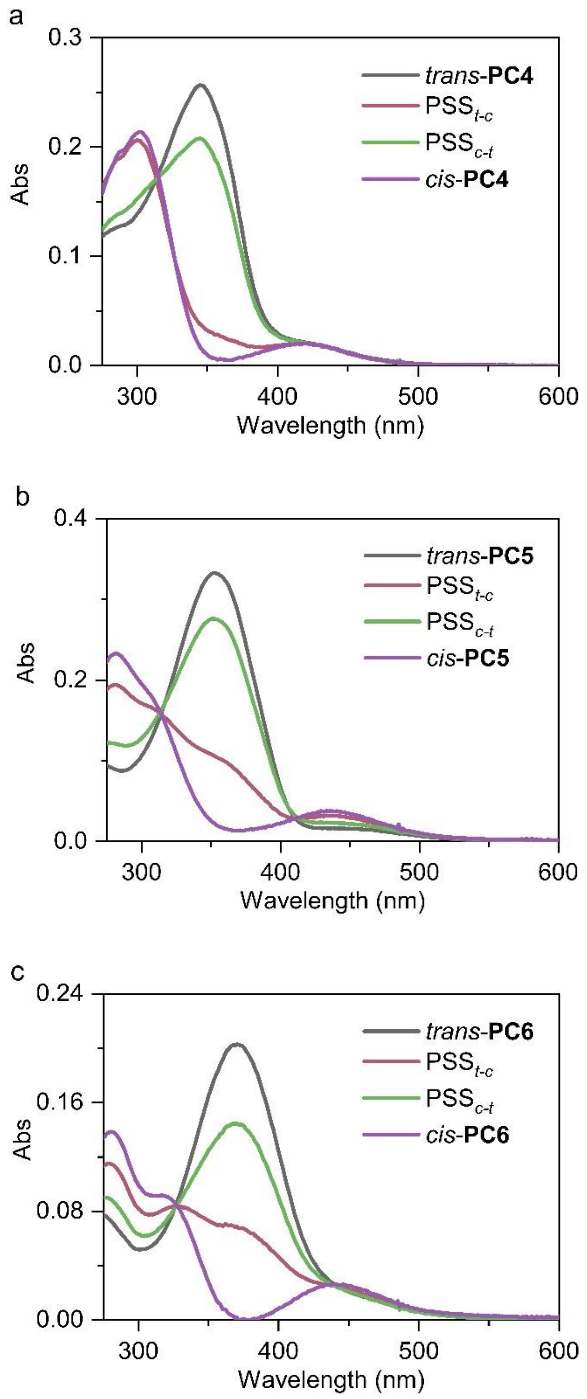

Figure S9. Absorption spectra of (a) **PC4** in a 75:25 DMSO:H_2O mixture (c = 8.9 μM), (b) **PC5** in a 50:50 DMSO:H_2O mixture (c = 14.3 μM), and (c) **PC6** in a 40:60 DMSO:H_2O mixture (c = 8.0 μM). For each compound, absorption spectra are given for the *trans* isomer, the *cis* isomer (estimated as described in section 1.3), the photostationary state mixture $PSS_{t\text{-}c}$ obtained upon irradiation at λ_{exc}= 365 nm to induce *trans*-*cis* photoisomerization, and the photostationary state mixture $PSS_{c\text{-}t}$ reached under visible light irradiation to induce *cis*-*trans* photoisomerization (λ_{exc}= 405 nm (**PC4**) or 445 nm (**PC5**, **PC6**)).

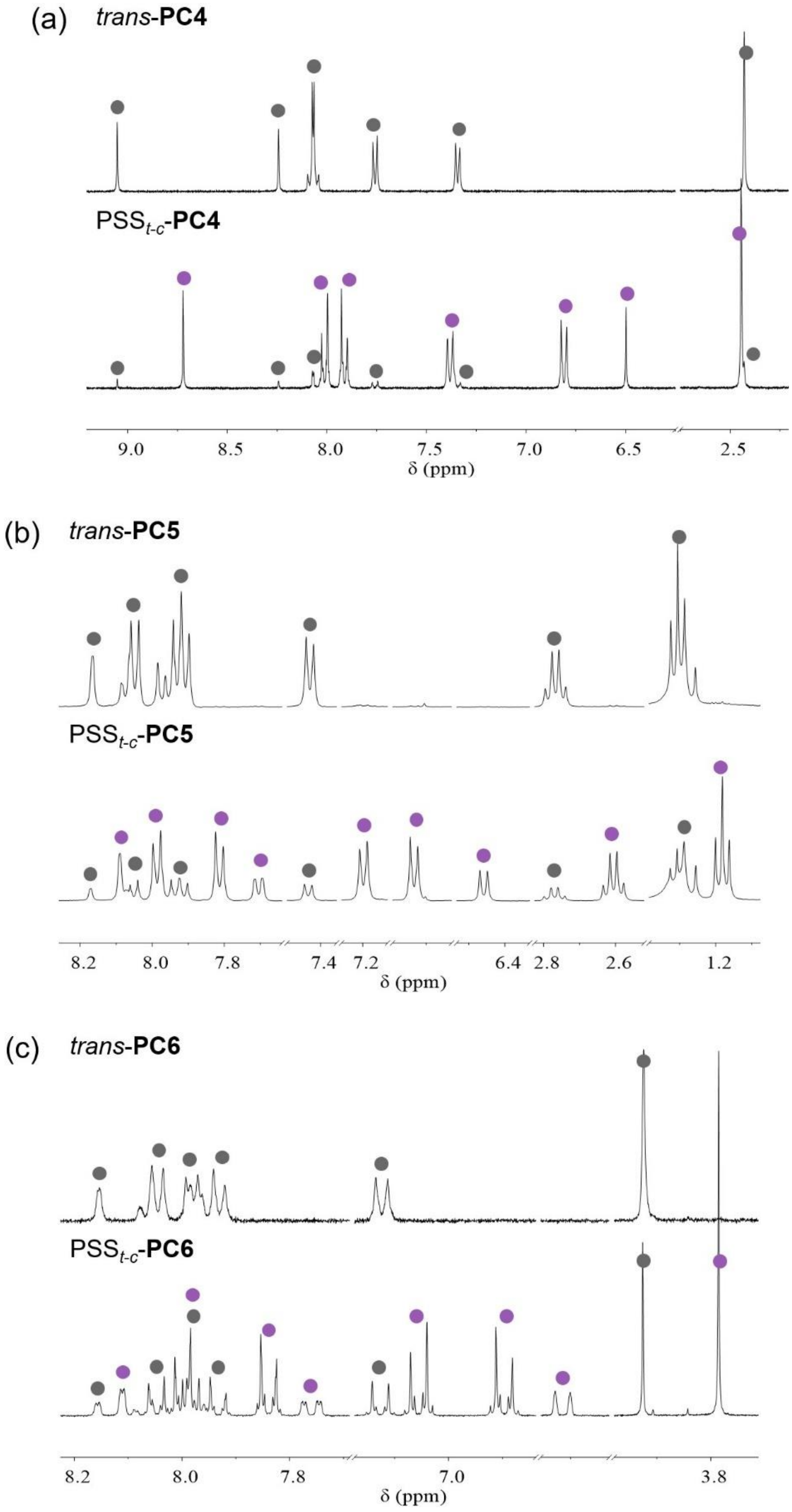

Figure S10. ^{1}H-NMR (300 MHz, methanol-*d*4) spectra for (a) **PC4**, (b) **PC5** and (c) **PC6**. For each compound, data is shown for (top) the initial *trans* isomer in the dark, and (bottom) the photostationary state mixture PSS$_{t\text{-}c}$ obtained upon irradiation at λ_{exc}= 365 nm to induce *trans*-*cis* photoisomerization. Gray and magenta circles are used to mark the NMR resonances arising from the *trans* and *cis* isomers of these compounds, respectively.

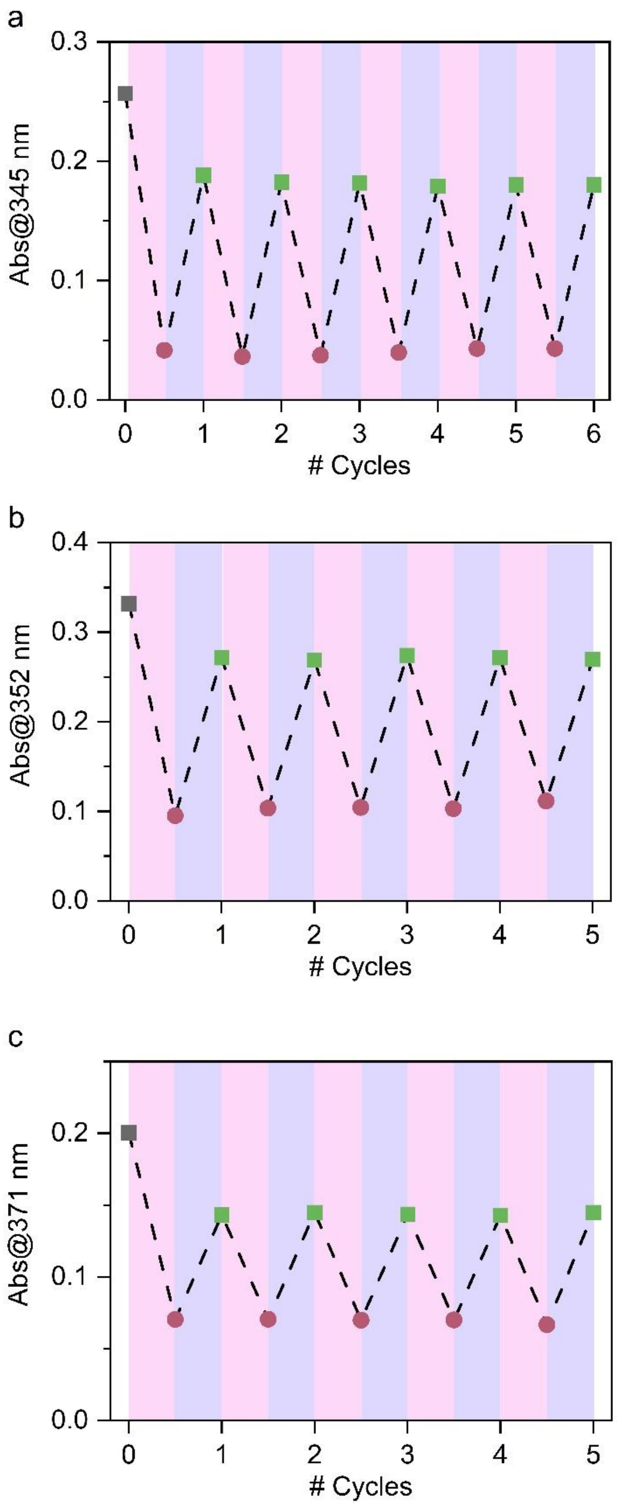

Figure S11. Variation of the absorbance of (a) *trans*-**PC4** in 75:25 DMSO:H_2O (c = 8.9 μM), (b) *trans*-**PC5** in 50:50 DMSO:H_2O (c = 14.3 μM), and (c) *trans*-**PC6** in 40:60 DMSO:H_2O (c = 8.0 μM) for five consecutive cycles of UV (λ_{exc} = 365 nm, violet) and visible light irradiation (λ_{exc} = 405 (**PC4**) or 445 nm (**PC5** and **PC6**), blue) to repetitively promote *trans-cis* and *cis-trans* photoisomerization. In each case, variation of absorbance is plotted for the spectral maximum of the π-π* absorption band of the *trans* isomer (λ_{abs} = 345, 352 and 371 nm for **PC4**, **PC5** and **PC6**).

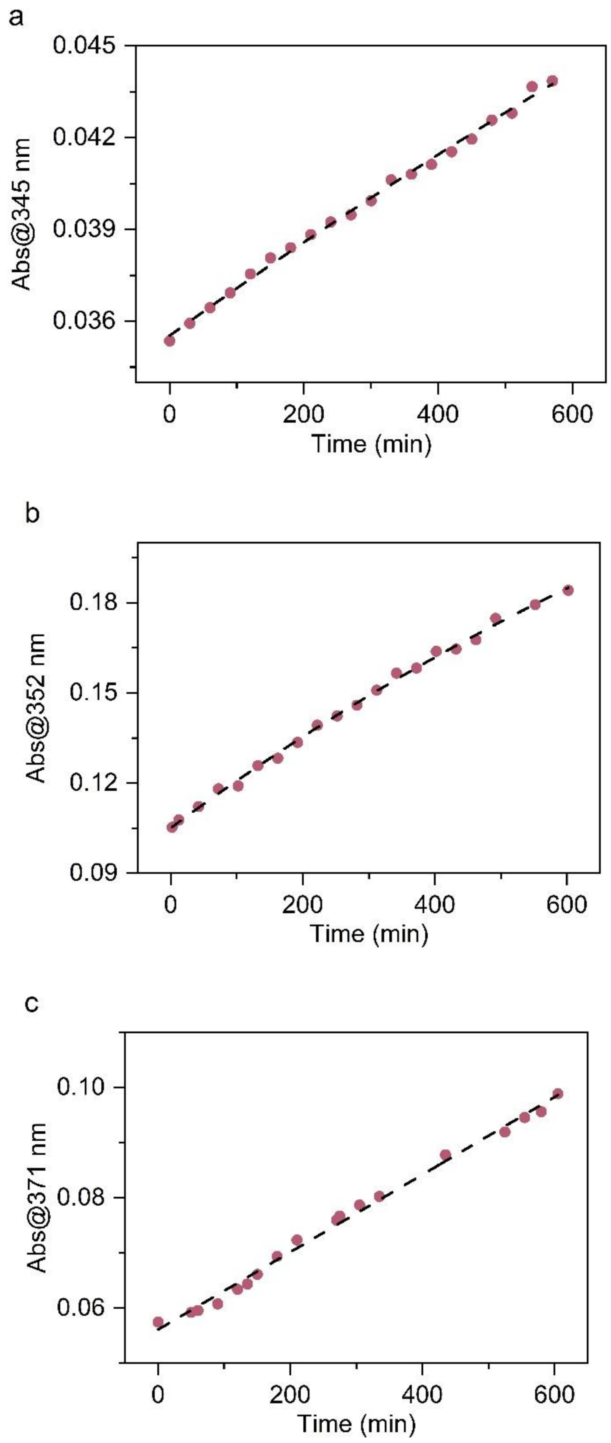

Figure S12. Variation of the absorbance in the dark at 25 °C of the $PSS_{t\text{-}c}$ mixture produced upon irradiation at λ_{exc} = 365 nm for (a) **PC4** in 75:25 DMSO:H_2O (c = 8.9 µM), (b) **PC5** in 50:50 DMSO:H_2O (c = 14.3 µM), and (c) **PC6** in in 40:60 DMSO:H_2O (c = 8.0 µM). In each case, variation of absorbance is plotted for the spectral maximum of the π-π* absorption band of the *trans* isomer (λ_{abs} = 345, 352 and 371 nm for **PC4**, **PC5** and **PC6**). At these conditions, thermal *trans-cis* back-isomerization takes place, which we fitted to a monoexponential kinetic model to obtain half-lives for the *cis* isomer of $t_{1/2}$ = 37 (**PC4**), 17 (**PC5**) and 10 h (**PC6**). Points correspond to the experimental data, while lines were obtained from monoexponencial fits.

Table S2. Photochemical properties of **PC4**, **PC5** and **PC6** in DMSO:H_2O mixtures.[a]

	$\lambda^{t}_{abs,max}$ (nm)[b]	$\lambda^{c}_{abs,max}$ (nm)[c]	$t^{c}_{1/2}$ (h)[d]	$PSS_{t\text{-}c}$ (%)[e]	$PSS_{c\text{-}t}$ (%)[f]	$\Phi_{t\text{-}c}$[g]	$\Phi_{c\text{-}t}$[h]
**PC4**	345, 409	302, 437	37	10:90	70:30	0.22 ± 0.03	0.11 ± 0.02
**PC5**	352, 427	281, 451	17	27:73	79:21	0.09 ± 0.01	0.33 ± 0.01
**PC6**	371, 446	281, 451	10	34:66	71:29	0.13 ± 0.01	0.31 ± 0.01

[a] **PC4**: 75:25 DMSO:H_2O; **PC5**: 50:50 DMSO:H_2O; **PC6**: 40:60 DMSO:H_2O. [b] Absorption maxima of the π-π* and n-π* bands of the *trans* isomer. [c] Absorption maxima of the π-π* and n-π* bands of the *cis* isomer. [d] Half-life of the *cis* isomer at room temperature. [e] *Trans*:*cis* molar ratio of the photostationary state mixture produced by promoting *trans-cis* photoisomerization at λ_{exc} = 365 nm. [f] *Trans*:*cis* molar ratio of the photostationary state mixture produced by promoting *cis-trans* photoisomerization at λ_{exc} = 405 (**PC4**) or 445 nm (**PC5** and **PC6**). [g] *Trans-cis* photoisomerization quantum yield at λ_{exc} = 365, measured using 1,2-bis(2-methyl-5-phenyl-3-thienyl)perfluorocyclopentene in hexane as a reference ($\Phi_{ring\ closing}$ = 0.59 [15]). [h] *Cis-trans* photoisomerization quantum yield at λ_{exc} = 445, measured using 1,2-bis(2-methyl-5-phenyl-3-thienyl)perfluorocyclopentene in hexane as a reference ($\Phi_{ring\ opening}$ = 0.013 [15]).

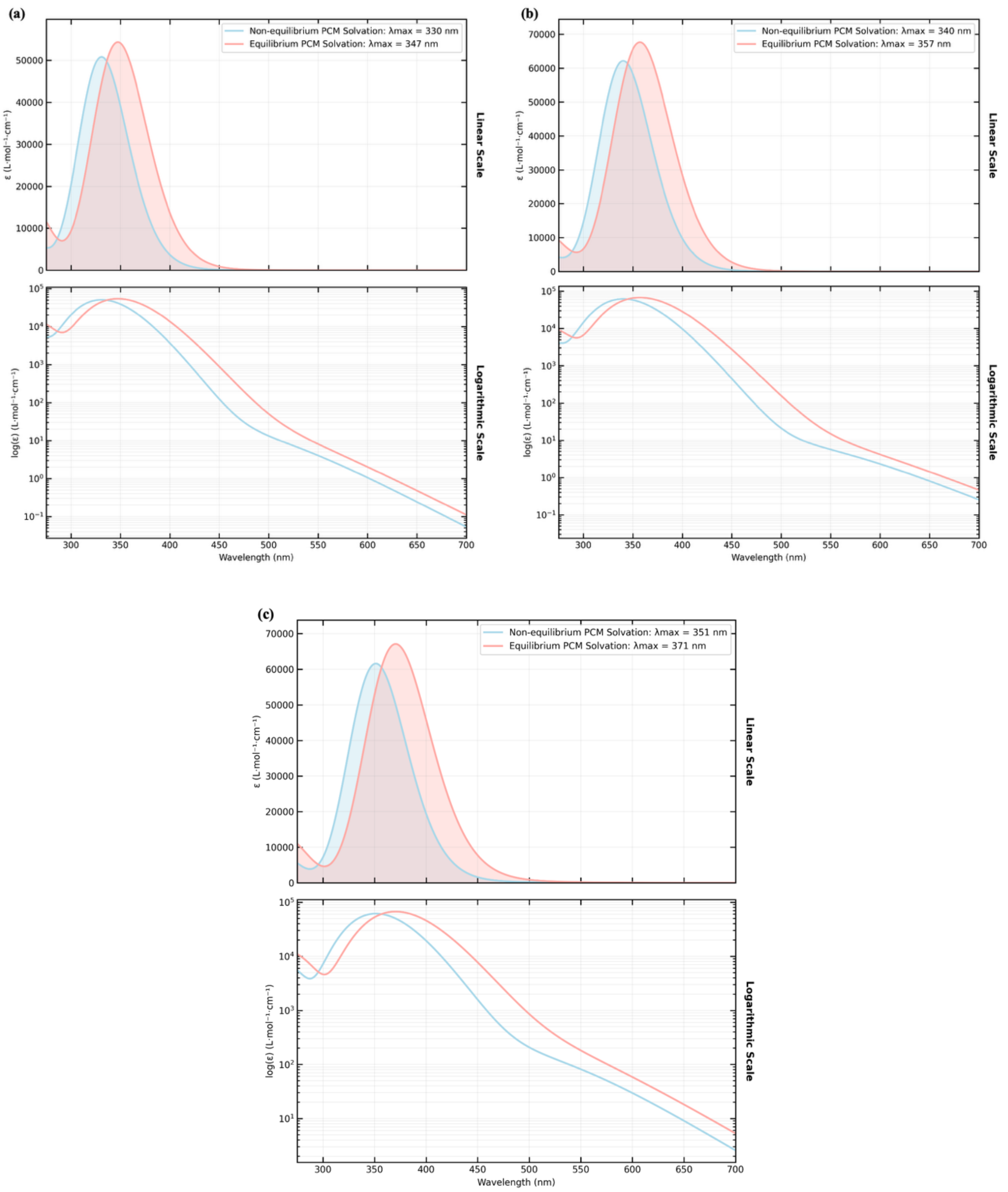

Figure S13. Absorption spectra calculations with non-equilibrium solvation (in blue) and with equilibrium solvation (in orange). Each plot shows the wavelength-dependent intensity over the 275-700 nm range. Plots (a) correspond to *trans*-**PC4**, (b) to *trans*-**PC5** and (c) to *trans*-**PC6** compounds.

Table S3. Computed optical transitions of *trans*-**PC4**, *trans*-**PC5** and *trans*-**PC6** in H_2O.[a]

		**Energy [eV]**	**Wavelength - λ [nm]**[b]	**f**[c]	**Excitation**
trans-**PC4**	S_1	2.70	459 (409)	0.001	H – 2 → L
	S_2	3.57	347 (345)	1.341	H → L
trans-**PC5**	S_1	2.50	497 (427)	0.000	H – 1 → L
	S_2	3.47	357 (352)	1.671	H → L
trans-**PC6**	S_1	2.56	485 (446)	0.006	H – 2 → L
	S_2	3.35	370 (371)	1.657	H → L

[a]Computed by TD-DFT at the M06-2X/6-31+G(d) level, using the implicit solvent model SMD to account for equilibrium solvation. Data is only given for the two lowest energy transitions, which correspond to the π-π* and n-π* absorption bands measured experimentally. [b]For comparison purposes, experimental absorption wavelengths are given in parentheses. [c]Computed oscillator strengths. [d]Only the main contribution to the excitation is shown, where H stands for HOMO and L for LUMO.

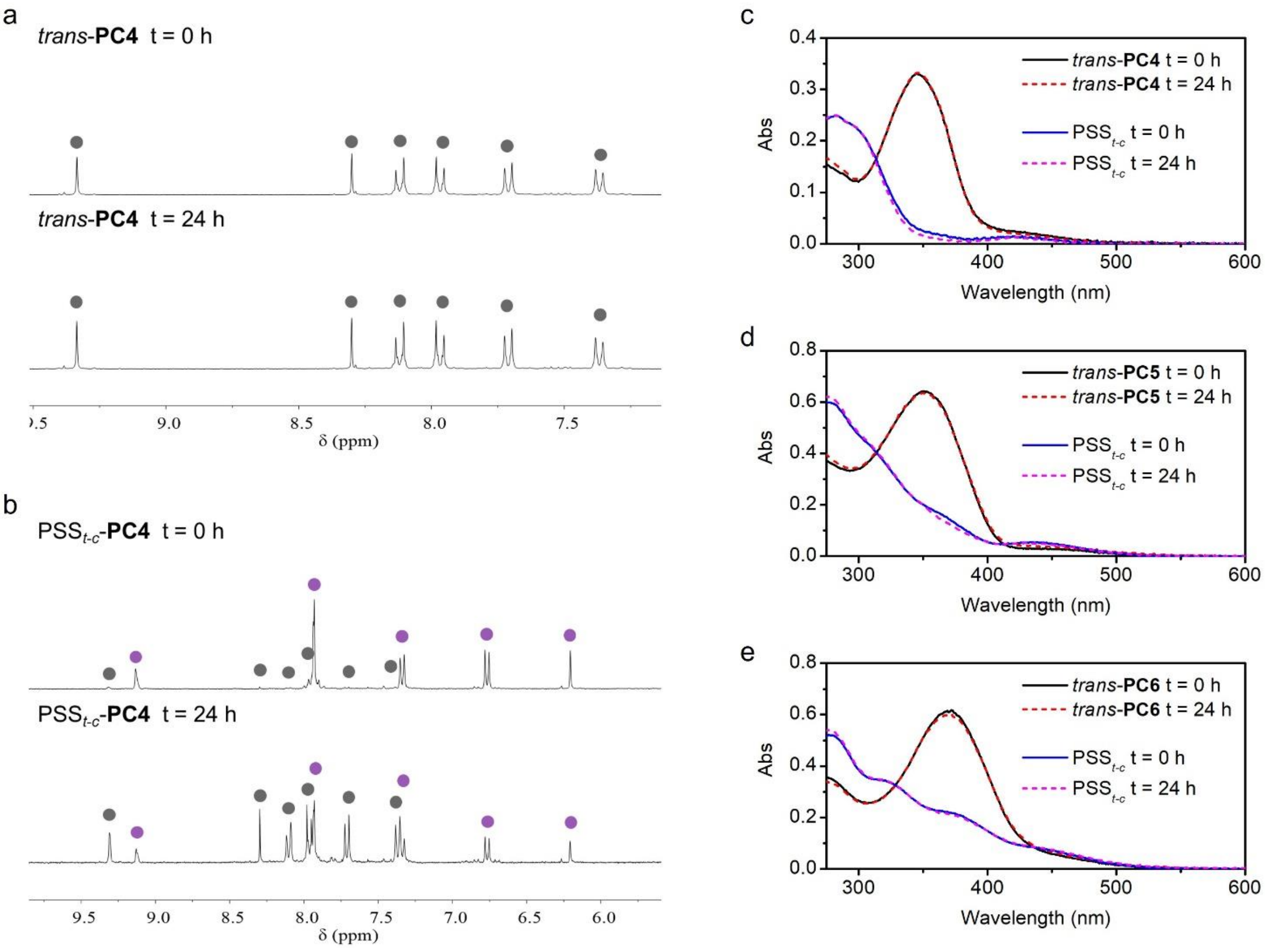

Figure S14. (a-b) ^{1}H NMR spectra (300 MHz) of (a) *trans*-**PC4** and (b) its $PSS_{t\text{-}c}$ state generated at λ_{exc} = 365 nm, which were measured before (t = 0 h) and after 24 h incubation (t = 24 h) in 90:10 DMSO-d_6:D_2O containing 10 mM glutathione (GSH) and 5 mM tris(2-carboxyethyl)phosphine (TCEP) at room temperature in the dark. Gray and magenta circles are used to mark the NMR resonances arising from the *trans* and *cis* isomers of **PC4**, respectively. The changes registered in (b) before and after incubation are due to thermal *cis*→*trans* back-photoisomerization, while the formation of new species arising from **PC4** reduction were not observed. (c-e) UV-vis absorption spectra of the *trans* isomer and the $PSS_{t\text{-}c}$ state generated at λ_{exc} = 365 nm of (b) **PC4**, (c) **PC5** and (d) **PC6**, which were measured before and after incubation in DMSO:H_2O mixtures containing 10 mM GSH and 5 mM TCEP at room temperature in the dark (**PC4**: 75:25 DMSO:H_2O; **PC5**: 50:50 DMSO:H_2O; **PC6**: 40:60 DMSO:H_2O). Because extensive thermal *cis*→*trans* back-photoisomerization takes place after 24 h, the UV-vis absorption spectra of $PSS_{t\text{-}c}$ after incubation were registered after previous irradiation at λ_{exc} = 365 nm to regenerate the photostationary state mixture.

5. ***In vivo*** **evaluation of anti-inflammatory activity on leukocyte migration**

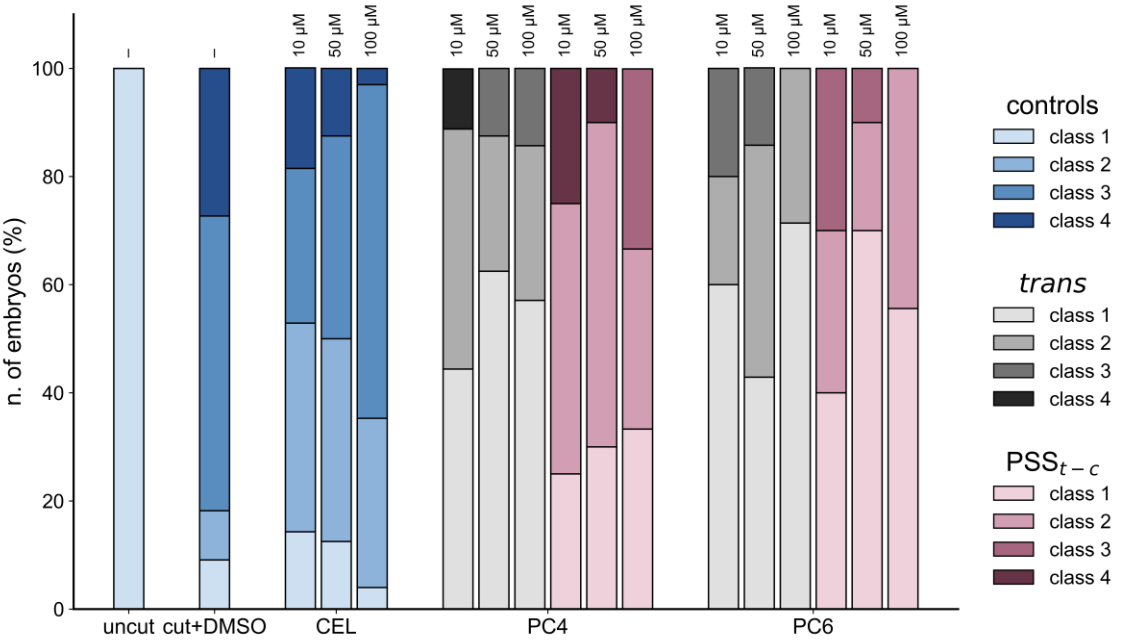

Figure S15. Number of embryos classified as class 1, 2, 3, and 4, based on the number of leukocytes migrated to the wound site at 6 hpa, after treatment with 10 µM, 50 µM, and 100 µM of CEL or **PC4-PC6**, in their *trans* or *cis* configuration.

6. Spectra of selected molecules

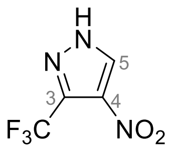

10a

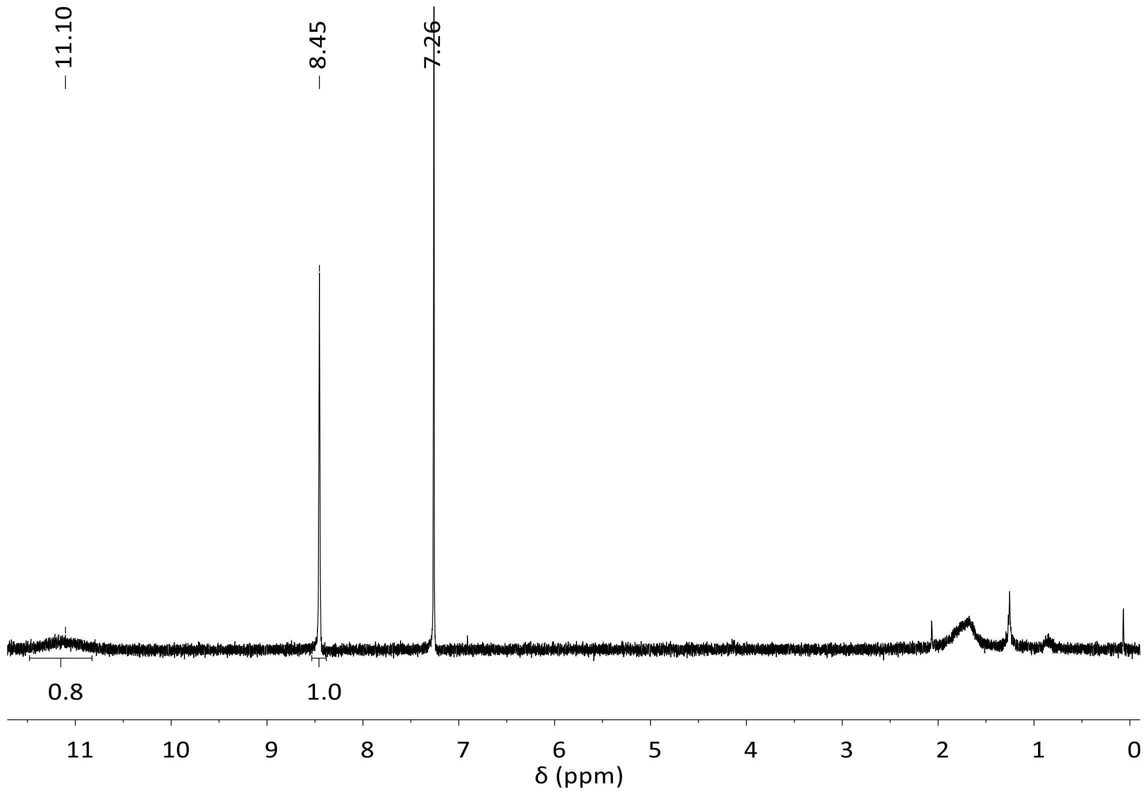

^1H NMR (300 MHz, $CDCl_3$)

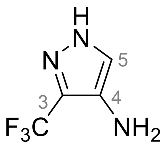

1a

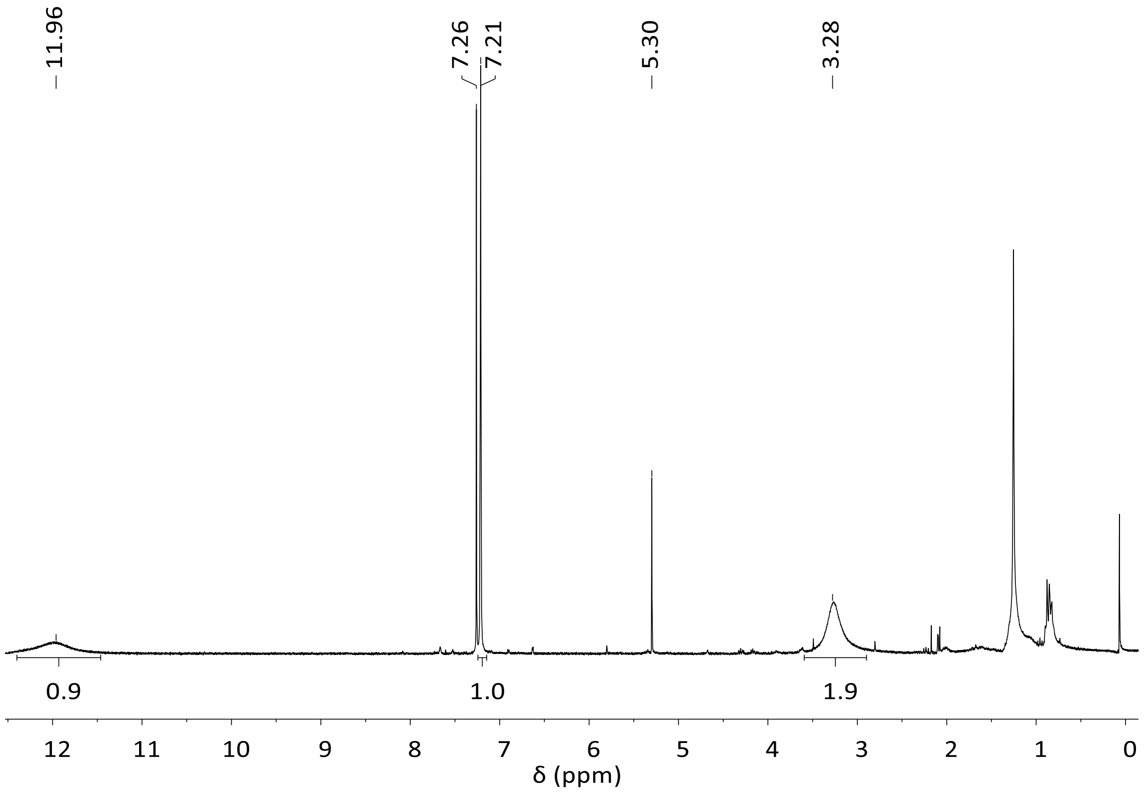

^{1}H NMR (300 MHz, $CDCl_3$)

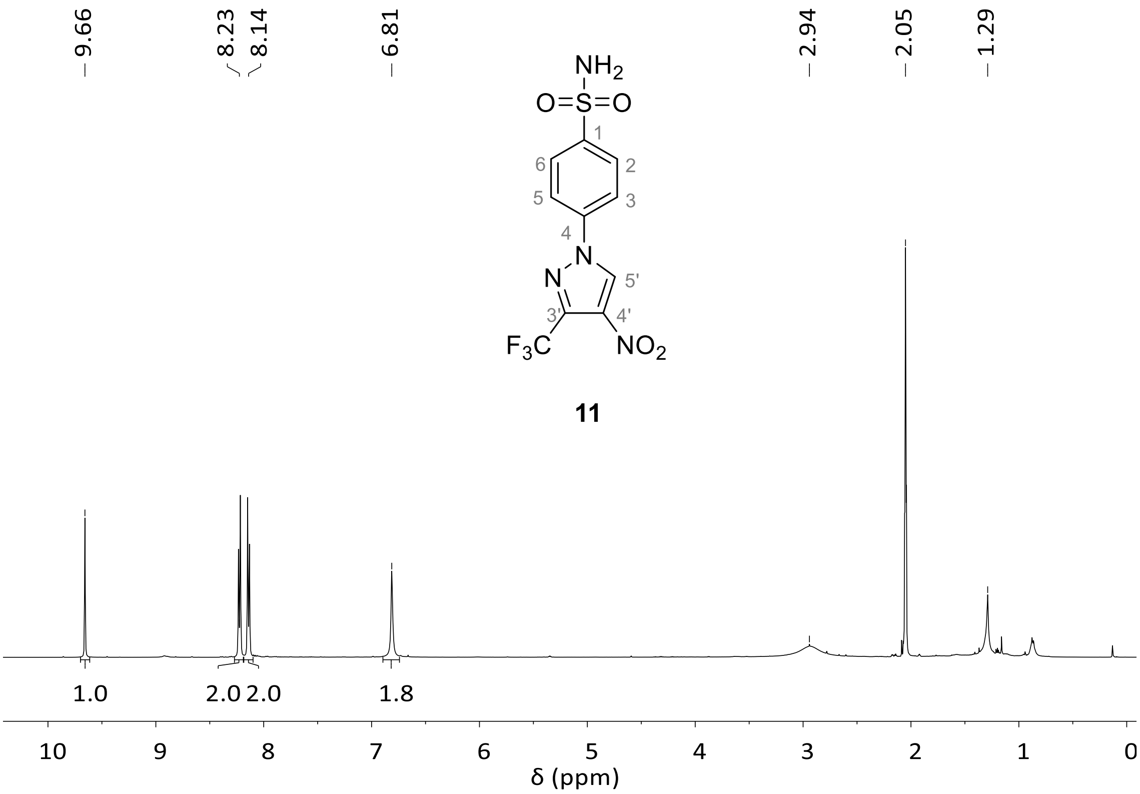

^{1}H NMR (500 MHz, acetone-d_6)

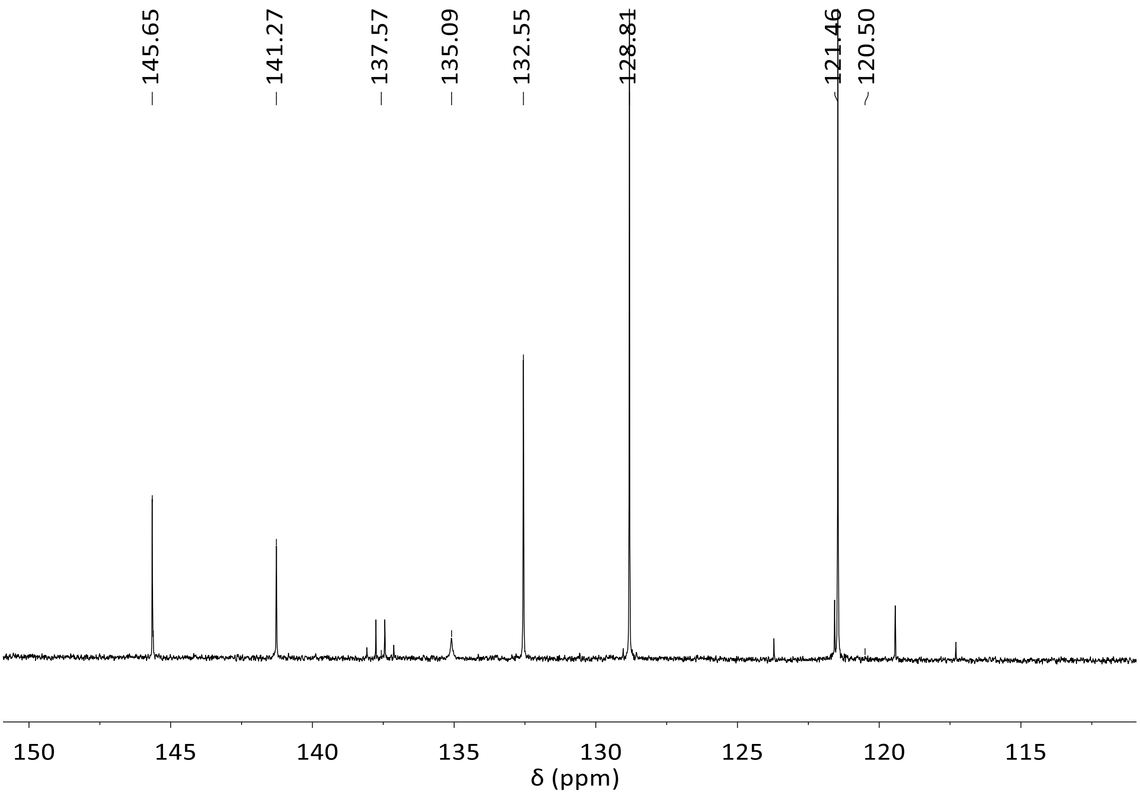

^{13}C NMR (125 MHz, acetone-d_6)

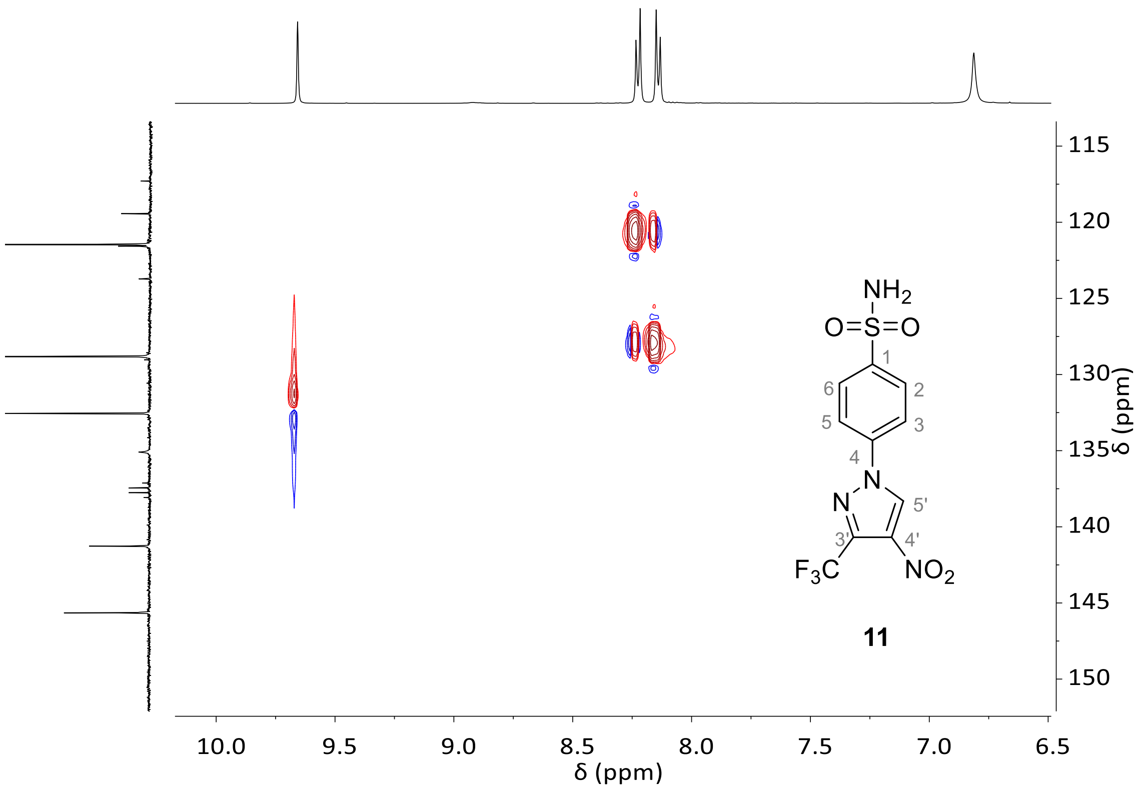

HSQC(ed) (500 MHz, 125 MHz, acetone-d_6)

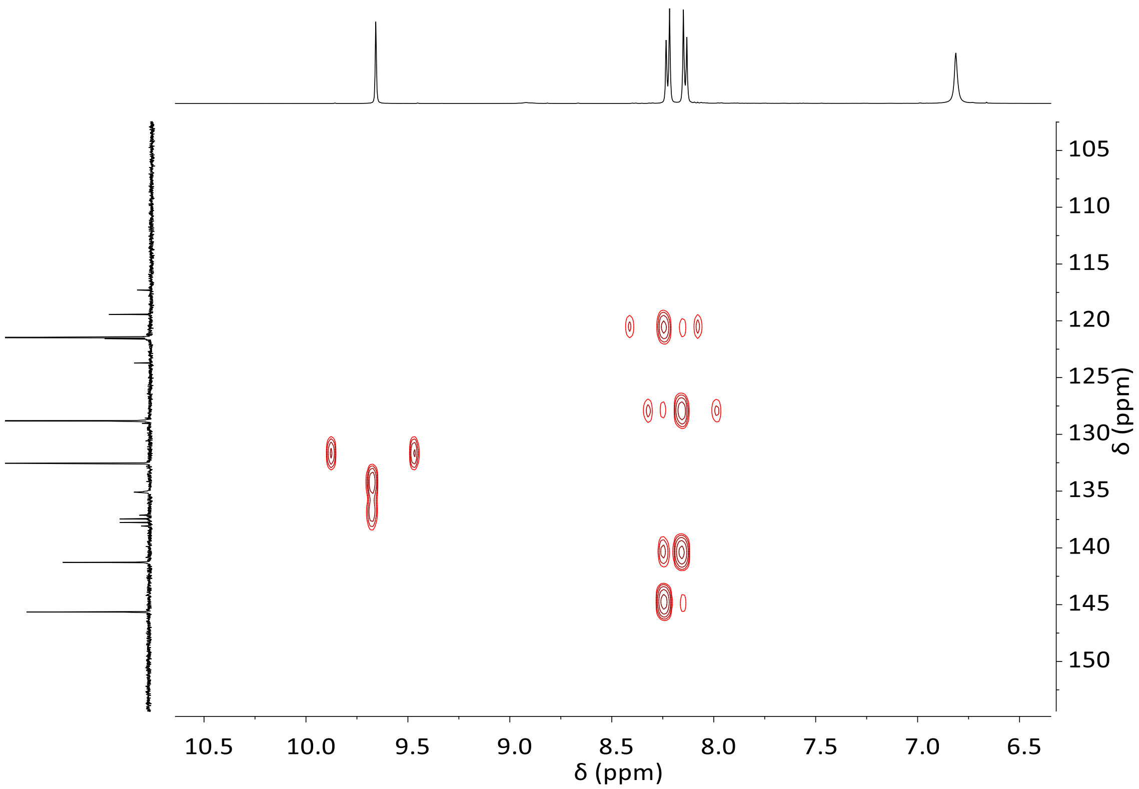

HMBC (500 MHz, 125 MHz, acetone-d_6)

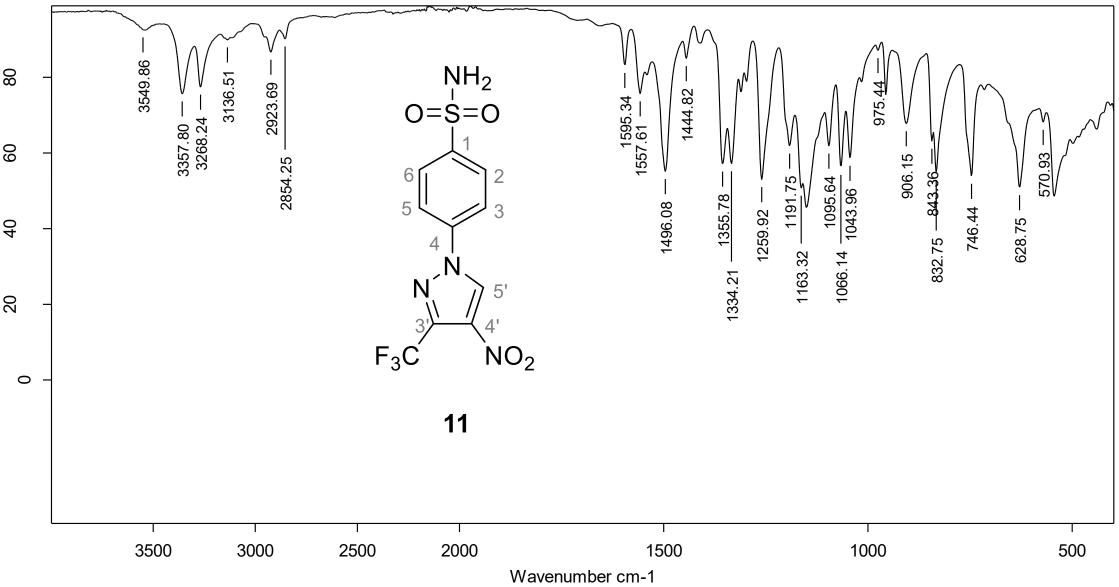
80
60
40
20
0
3549.86
3357.80
3268.24
3136.51
2923.69
2854.25
1595.34
1557.61
1496.08
1444.82
1355.78
1334.21
1259.92
1191.75
1163.32
1095.64
1066.14
1043.96
975.44
906.15
843.36
832.75
746.44
628.75
570.93
NH2
O=S=O
1
2
3
4
5
6
N
N
5'
3'
4'
F3C
NO2
11
3500
3000
2500
2000
1500
1000
500
Wavenumber cm-1

IR (ATR, cm^{-1})

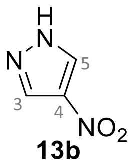

13b

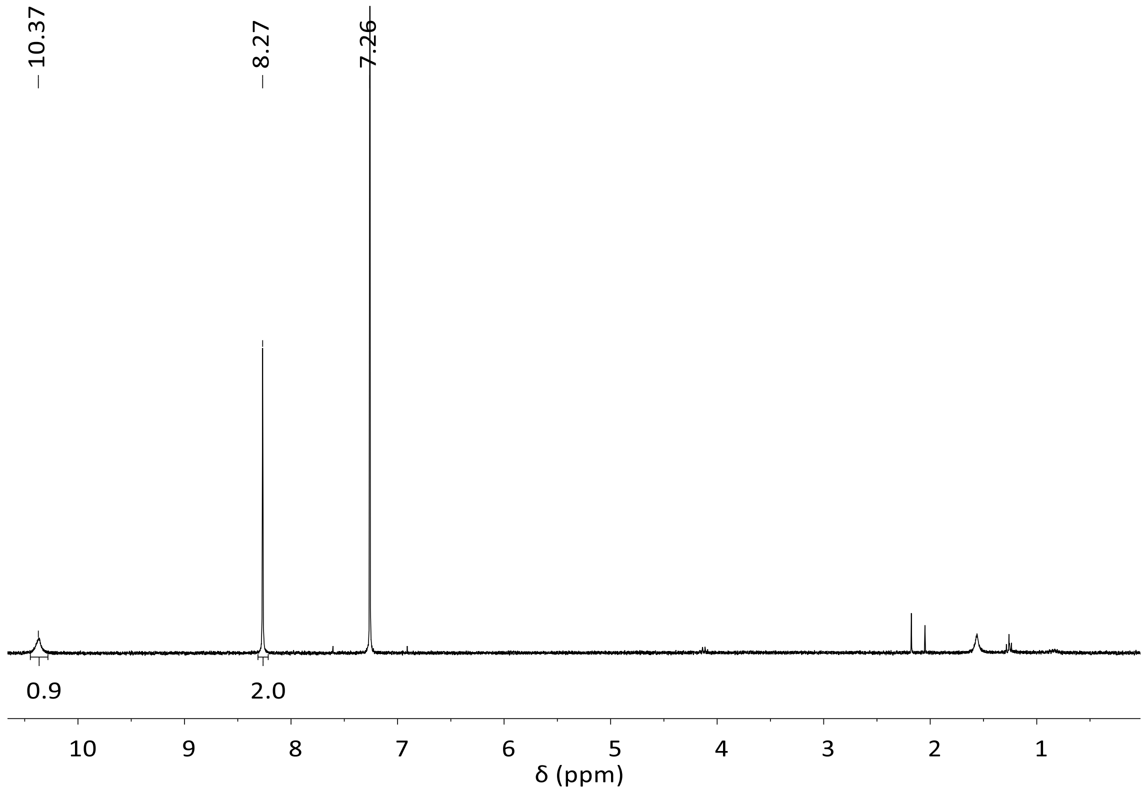

^{1}H NMR (300 MHz, $CDCl_3$)

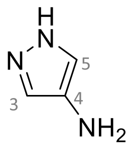

1b

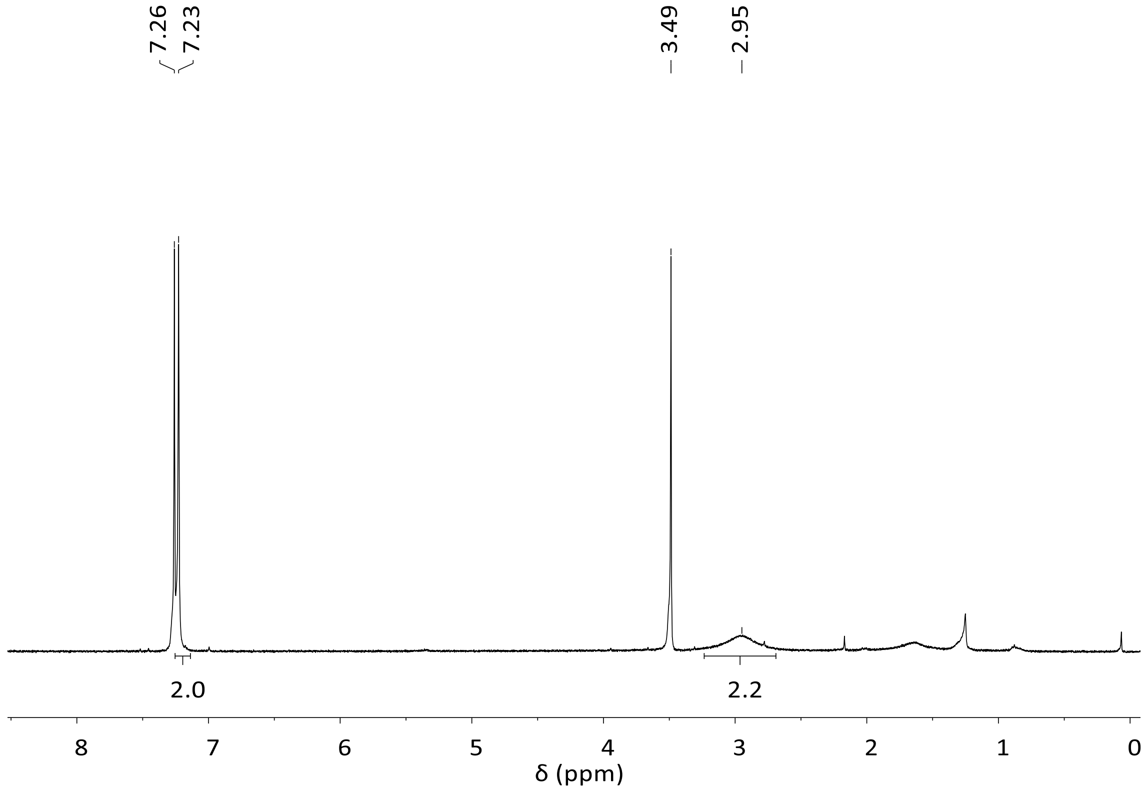

^{1}H NMR (300 MHz, $CDCl_3$)

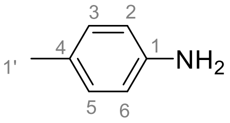

14

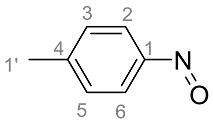

2

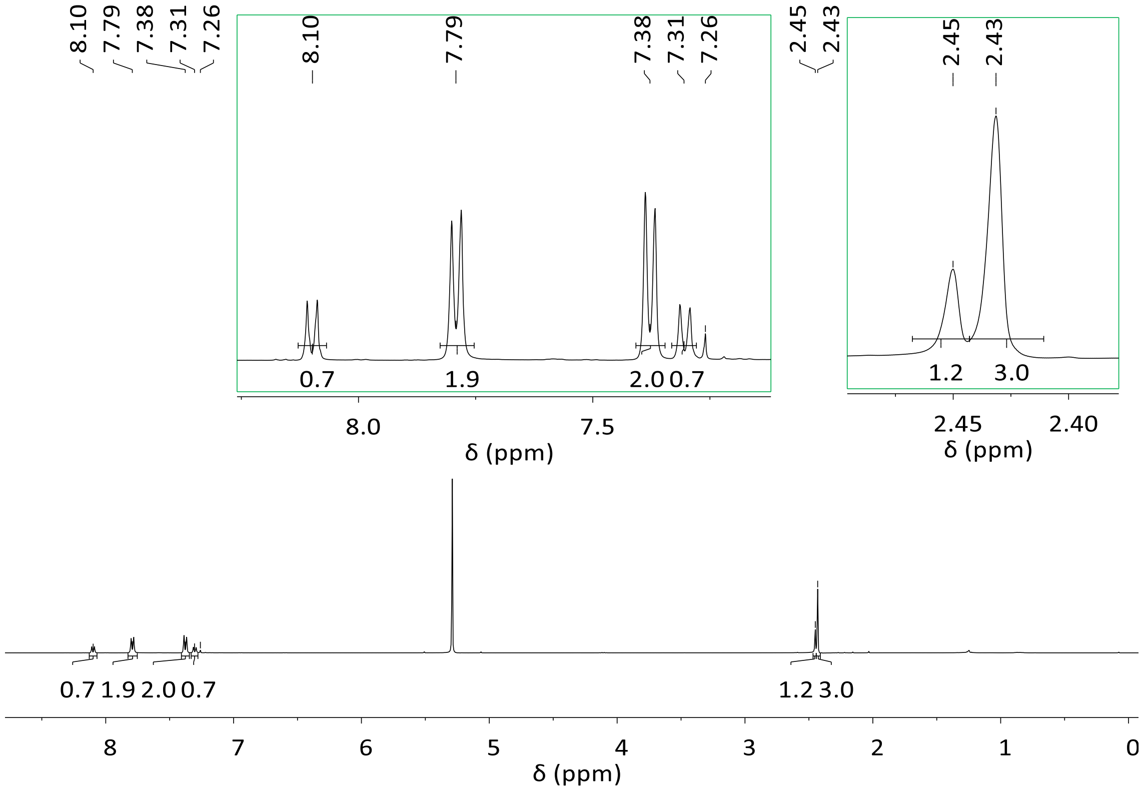

^{1}H NMR (400 MHz, $CDCl_3$)

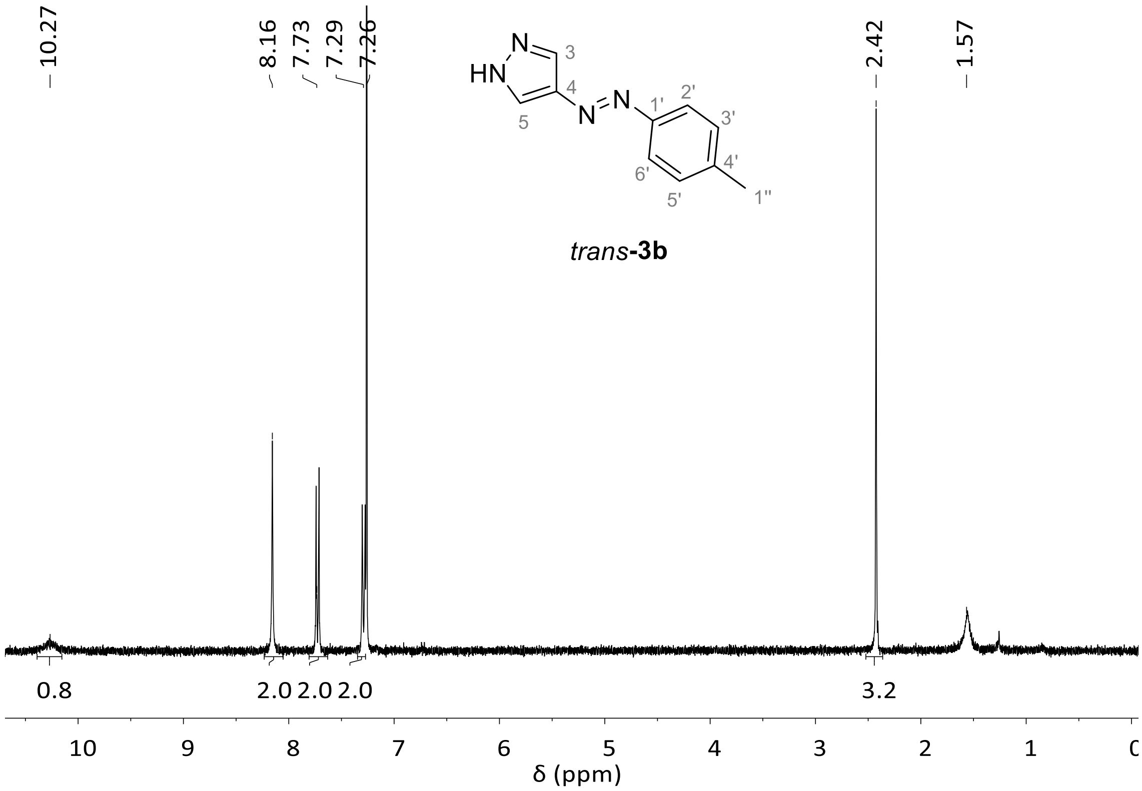

^{1}H NMR (300 MHz, $CDCl_3$)

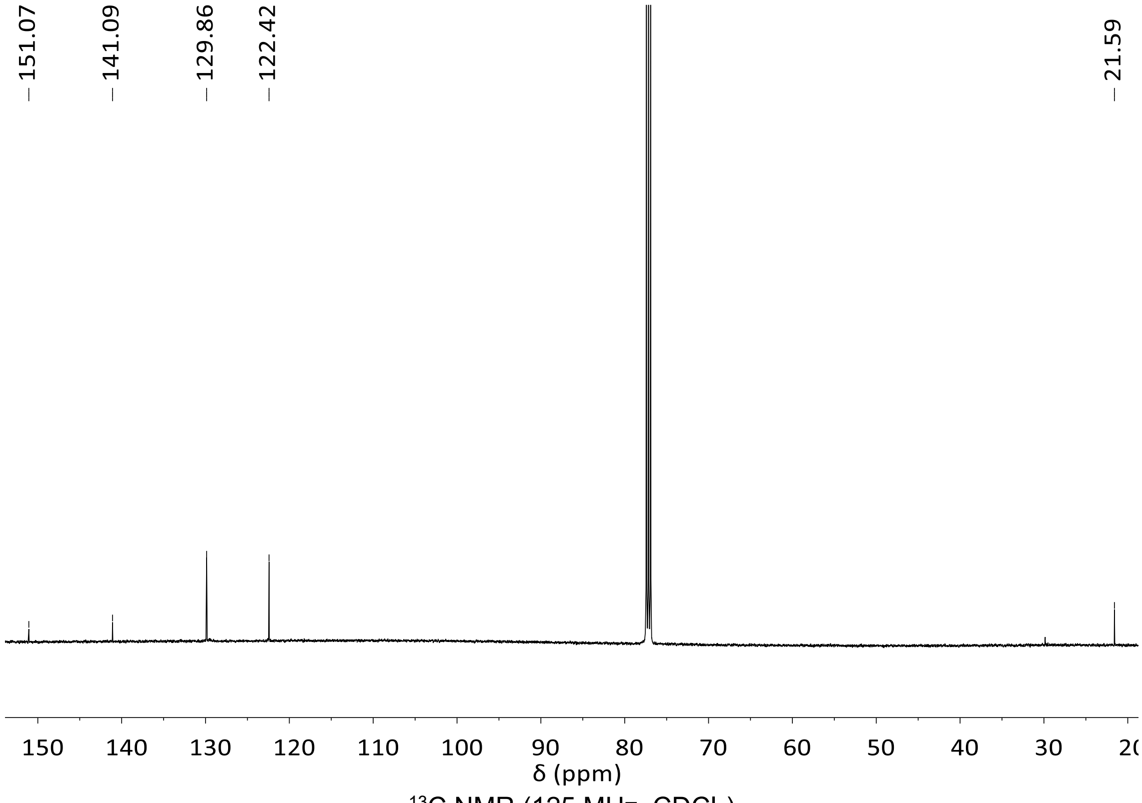

^{13}C NMR (125 MHz, $CDCl_3$)

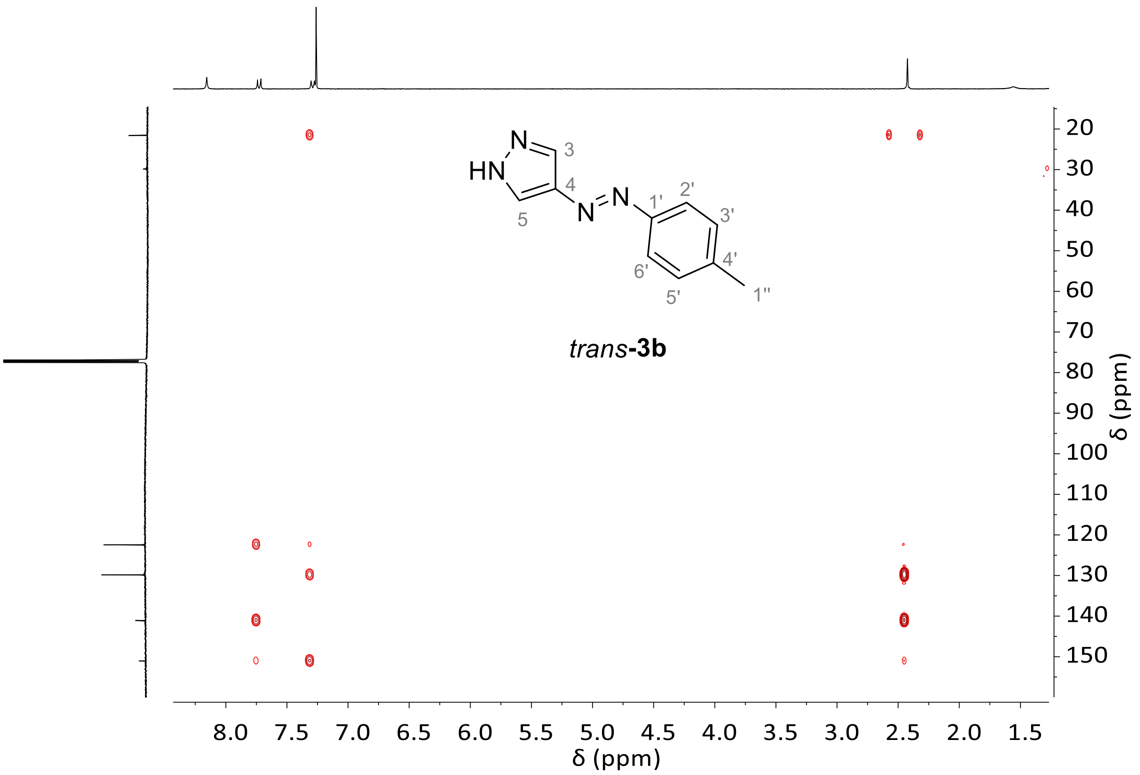

HMBC (500 MHz, 125 MHz, $CDCl_3$)

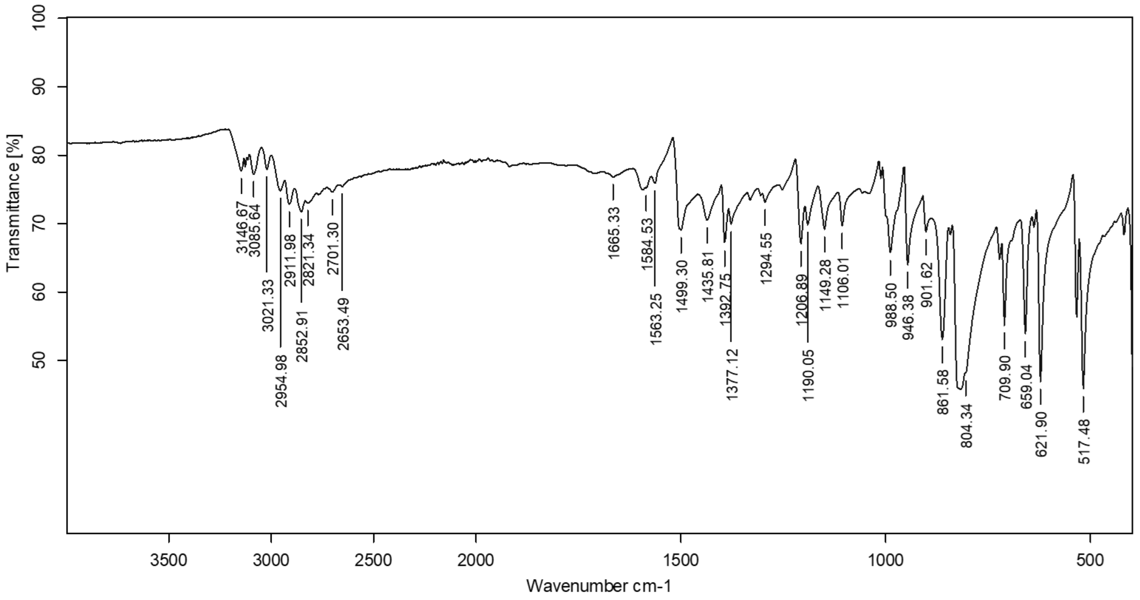

IR (ATR, cm^{-1})

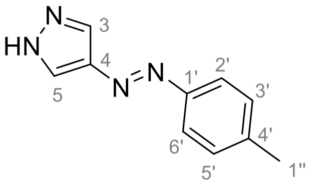

trans-**3b**

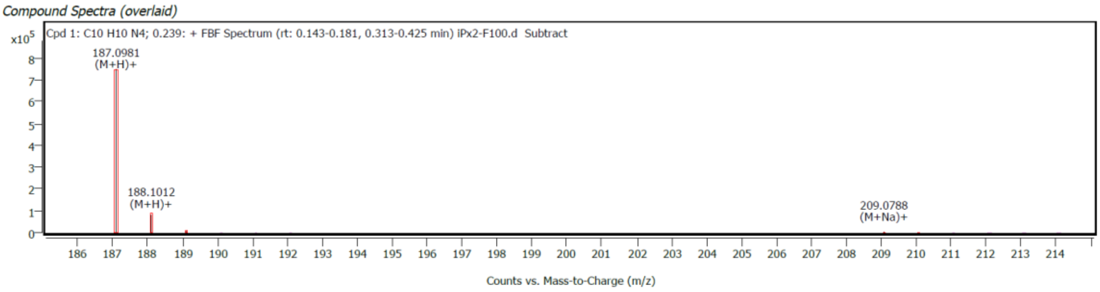

Spectrum Peaks Textbox28

m/z	m/z (Calc)	Diff (ppm)	Abund	Height %	Height % (Calc)	Z	Ion Species	Formula
187.0981	187.0978	1.55	750516	100.00	100.00	1	(M+H)+	C10H10N4
188.1012	188.1005	4.01	80459	10.72	12.40	1	(M+H)+	C10H10N4
189.1042	189.1030	6.44	4182	0.56	0.71	1	(M+H)+	C10H10N4
209.0788	209.0798	-4.55	1249	100.00	100.00	1	(M+Na)+	C10H10N4
210.0809	210.0824	-7.02	149	11.92	12.39	1	(M+Na)+	C10H10N4

HRMS (ESI(+))

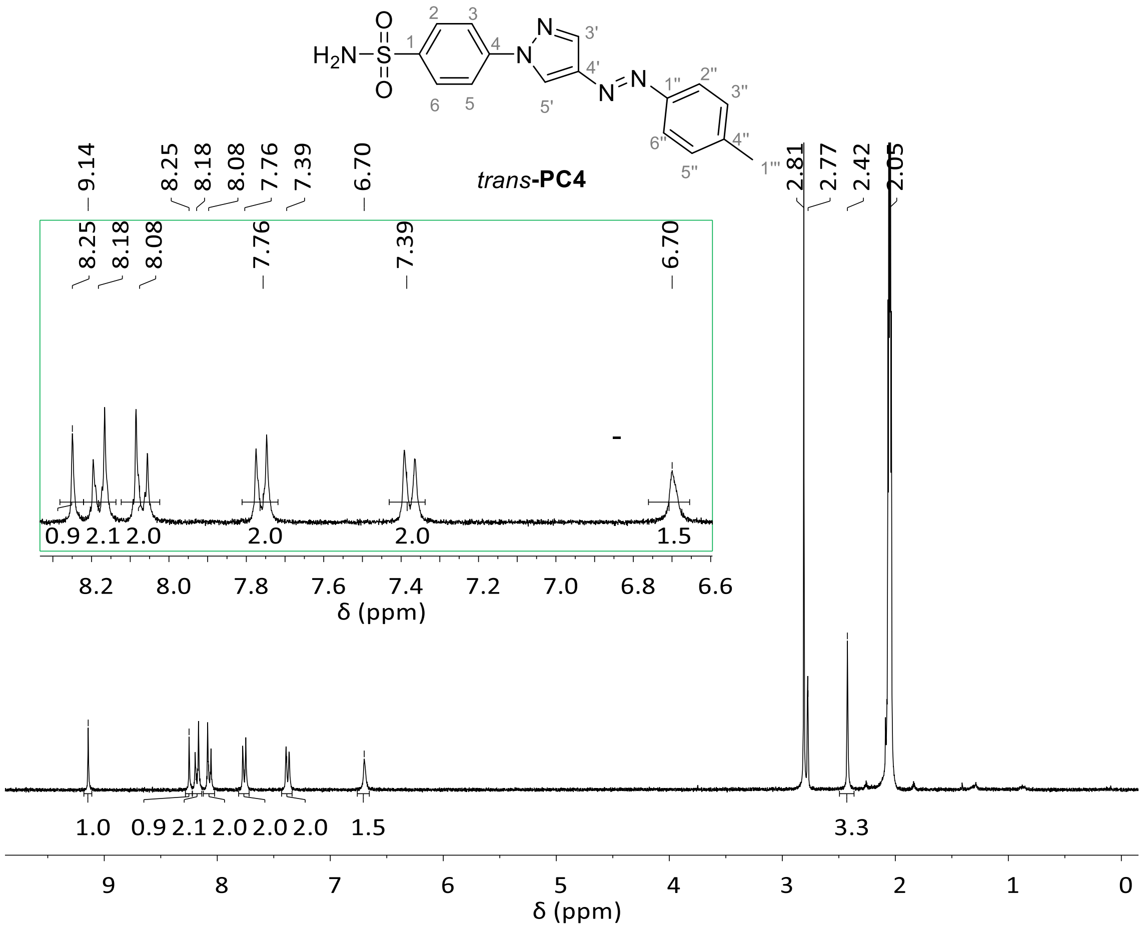

^{1}H NMR (300 MHz, acetone-d_6)

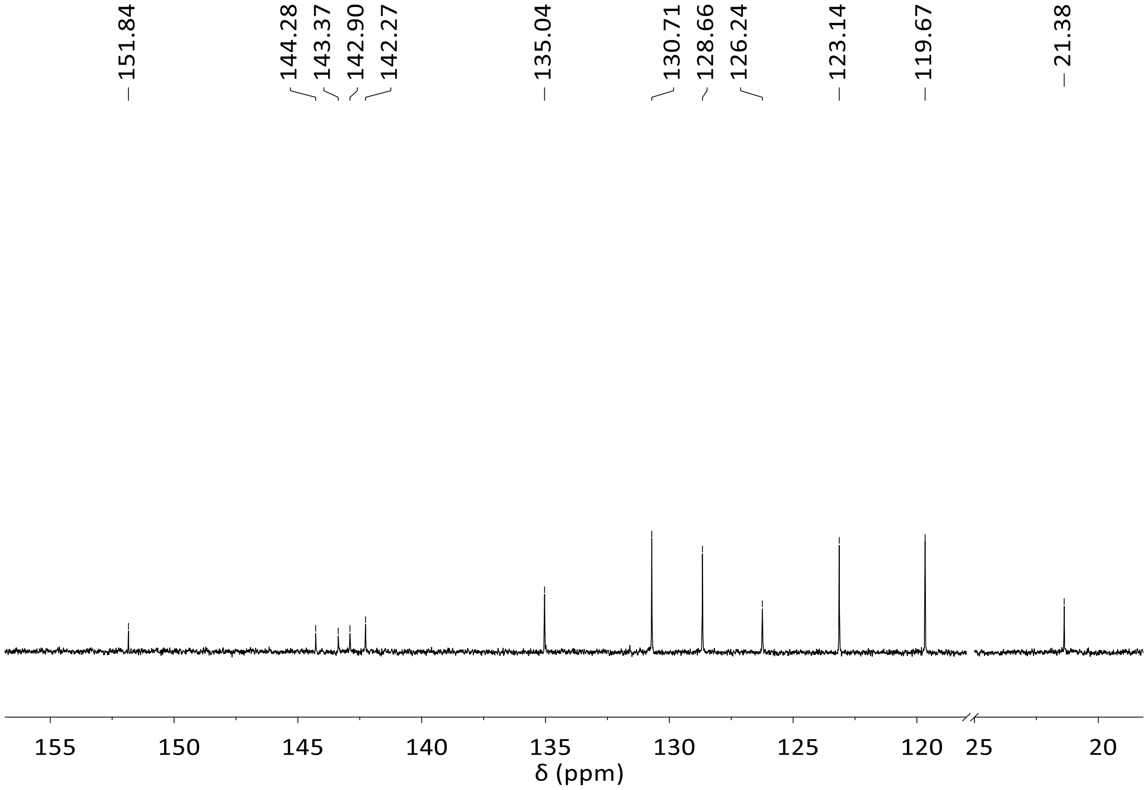

^{13}C NMR (125 MHz, acetone-d_6)

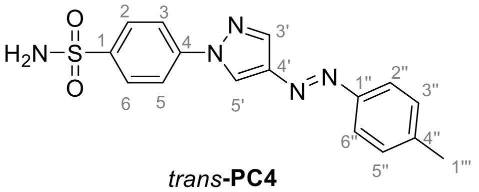

trans-**PC4**

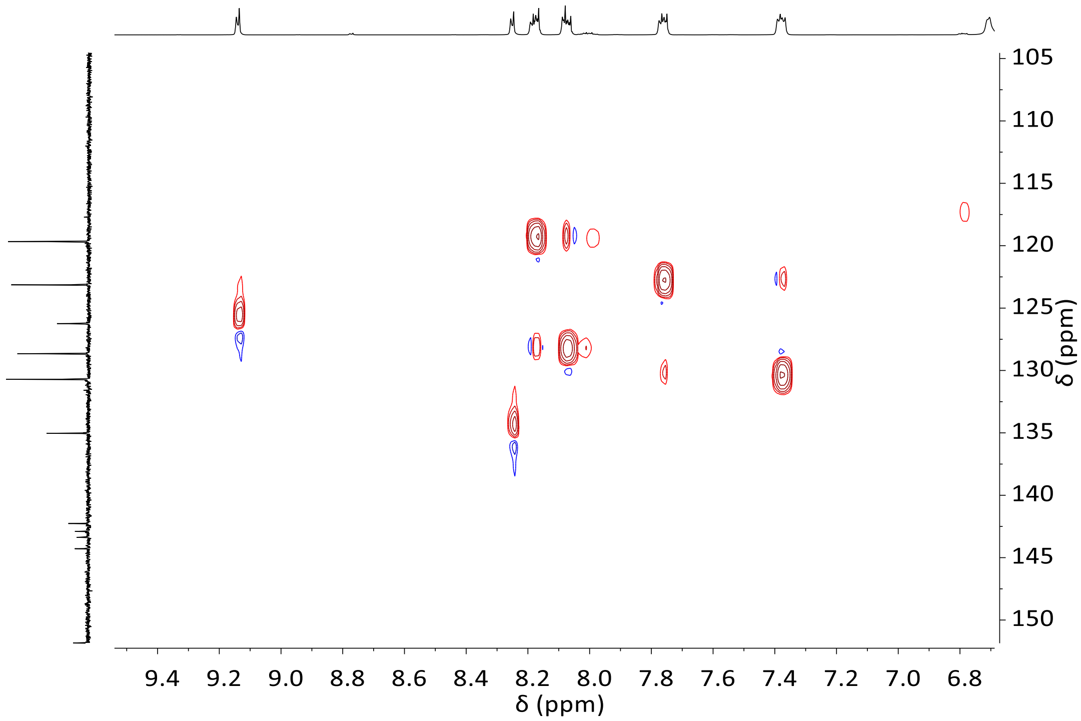

HSQC(ed) (500 MHz, 125 MHz, acetone-d_6)

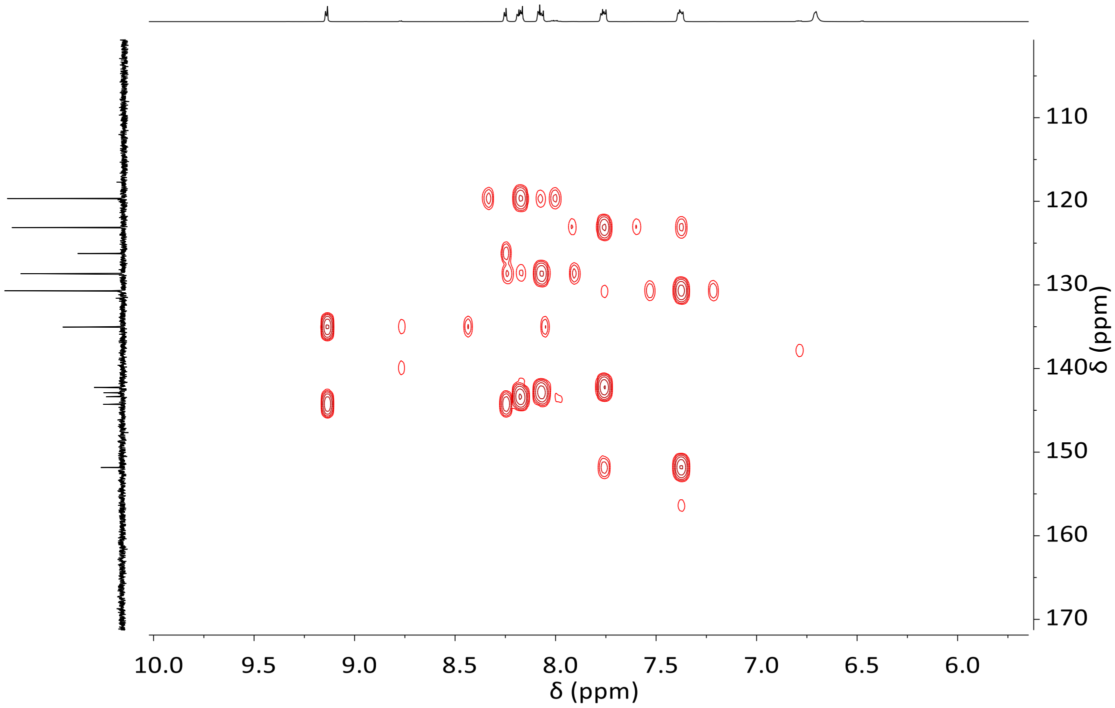

HMBC (500 MHz, 125 MHz, acetone-d_6)

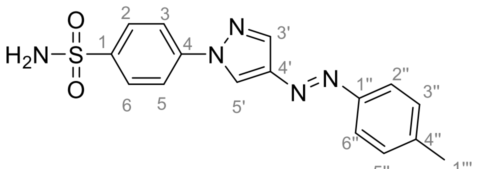

trans-**PC4**

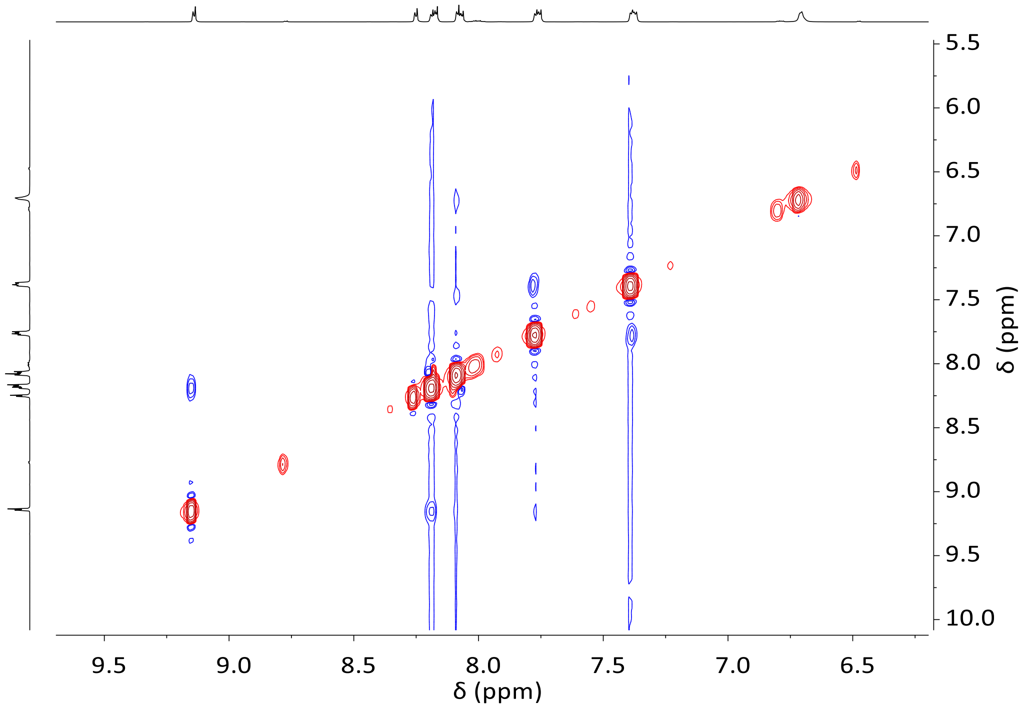

NOESY (500 MHz, acetone-d_6)

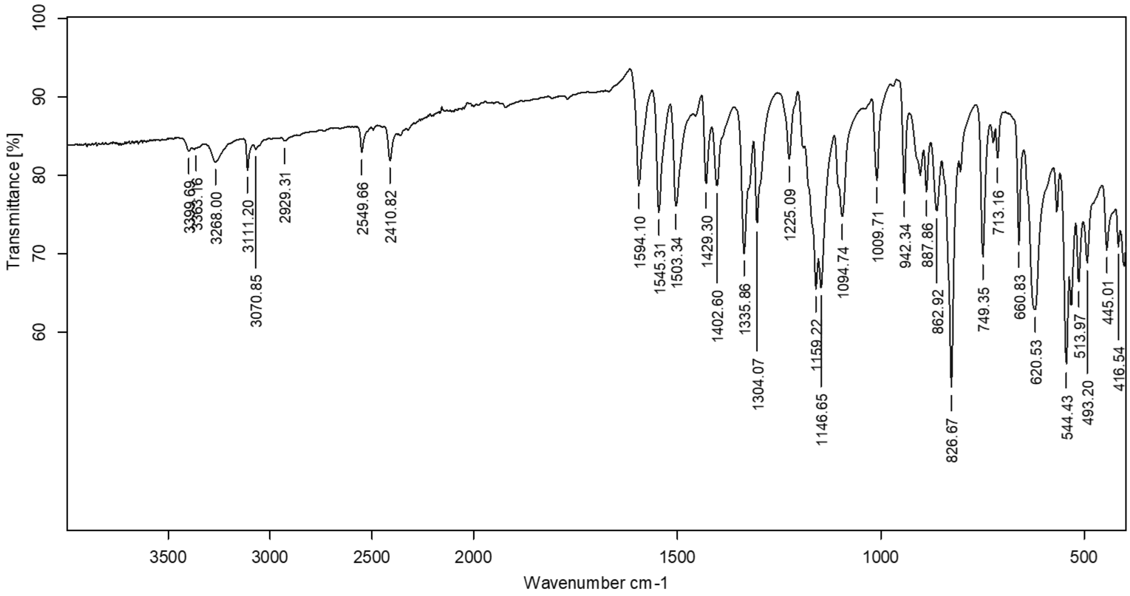

IR (ATR, cm^{-1})

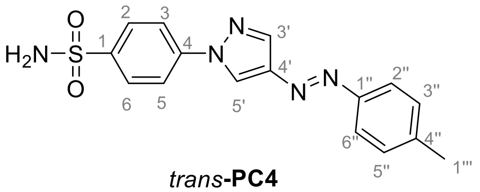

trans-**PC4**

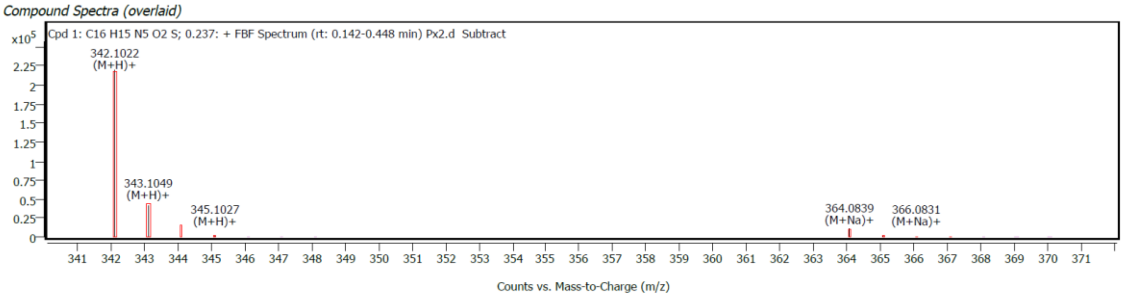

Spectrum Peaks Textbox28

m/z	m/z (Calc)	Diff (ppm)	Abund	Height %	Height % (Calc)	Z	Ion Species	Formula
342.1022	342.1019	0.72	219510	100.00	100.00	1	(M+H)+	C16H15N5O2S
343.1049	343.1046	1.07	41360	18.84	20.18	1	(M+H)+	C16H15N5O2S
344.1016	344.1009	1.88	11056	5.04	6.82	1	(M+H)+	C16H15N5O2S
345.1027	345.1022	1.61	1963	0.89	1.07	1	(M+H)+	C16H15N5O2S
364.0839	364.0839	0.11	11007	100.00	100.00	1	(M+Na)+	C16H15N5O2S
365.0875	365.0865	2.60	2174	19.75	20.17	1	(M+Na)+	C16H15N5O2S
366.0831	366.0829	0.65	614	5.57	6.82	1	(M+Na)+	C16H15N5O2S
367.0855	367.0841	3.89	149	1.35	1.07	1	(M+Na)+	C16H15N5O2S

HRMS (ESI(+))

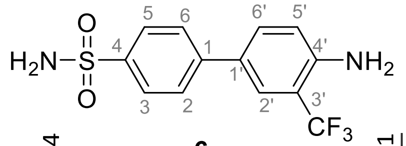

6

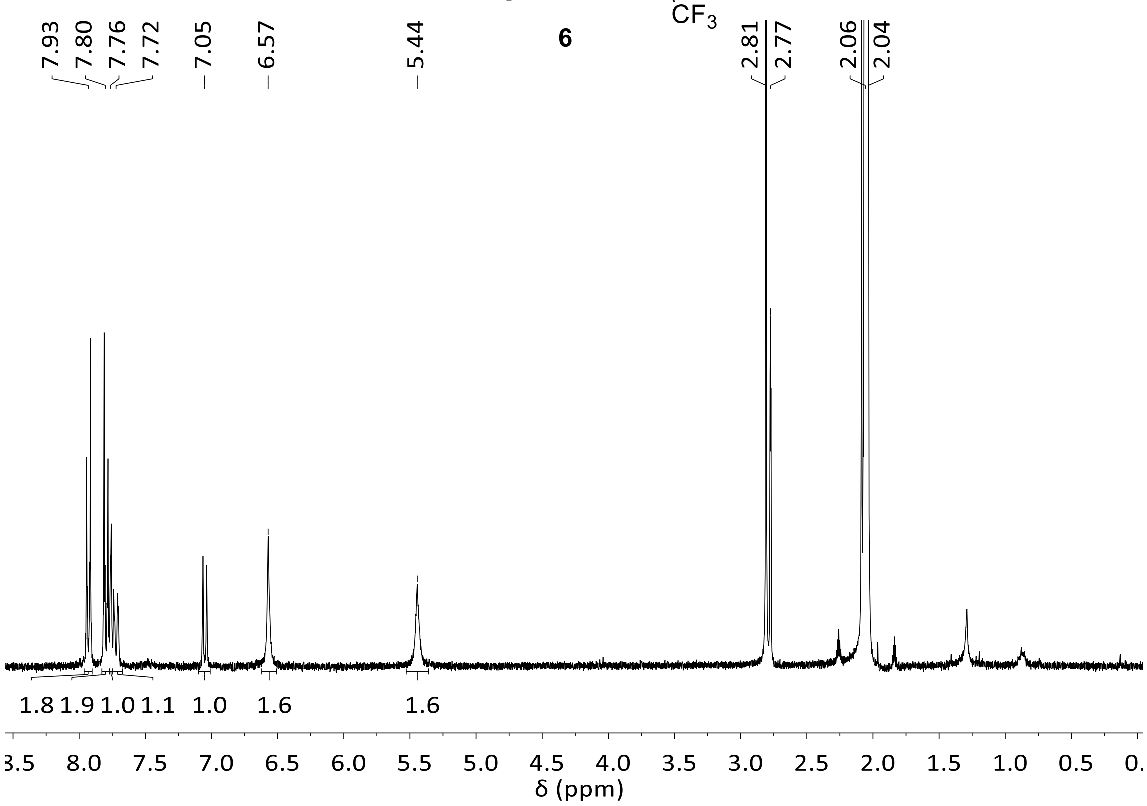

^{1}H NMR (300 MHz, acetone-d_6)

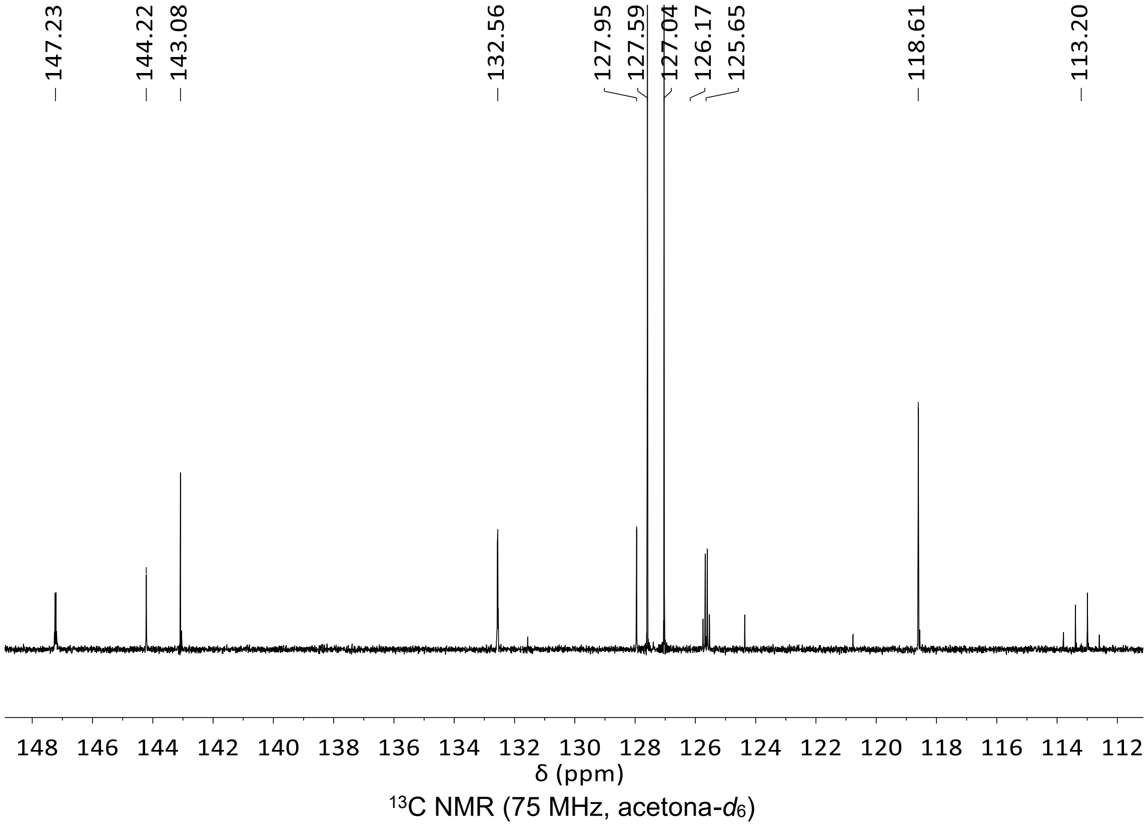

^{13}C NMR (75 MHz, acetona-d_6)

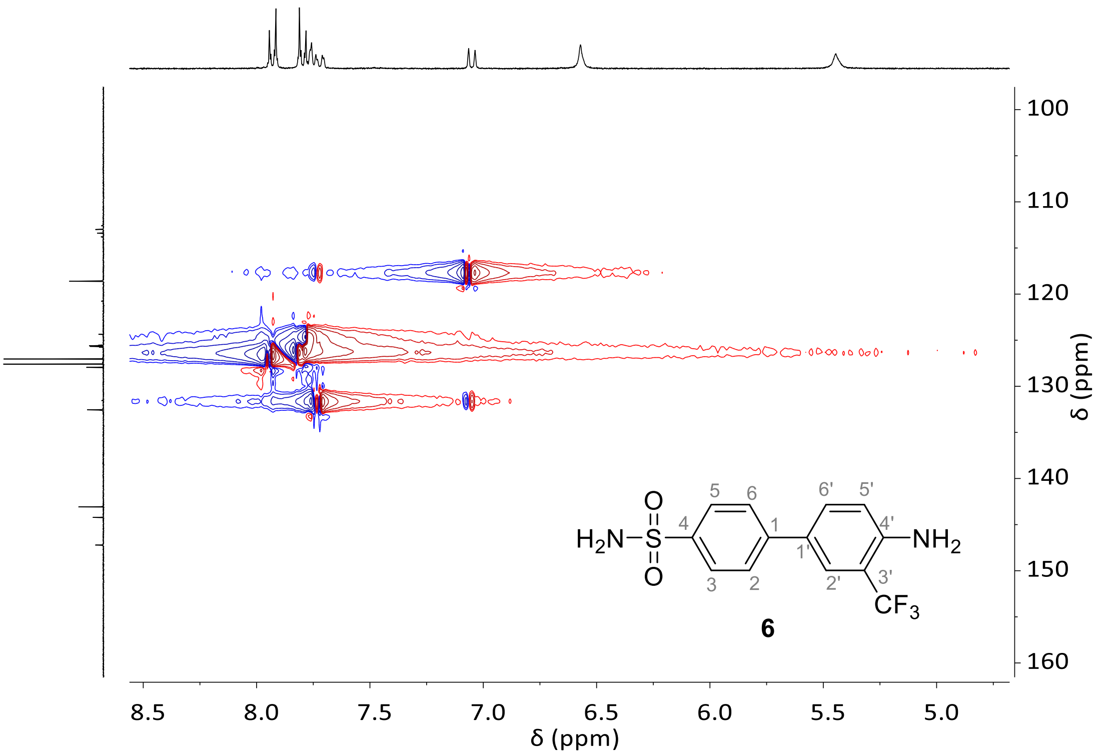

HSQC(ed) (300 MHz, 75 MHz, acetone-d_6)

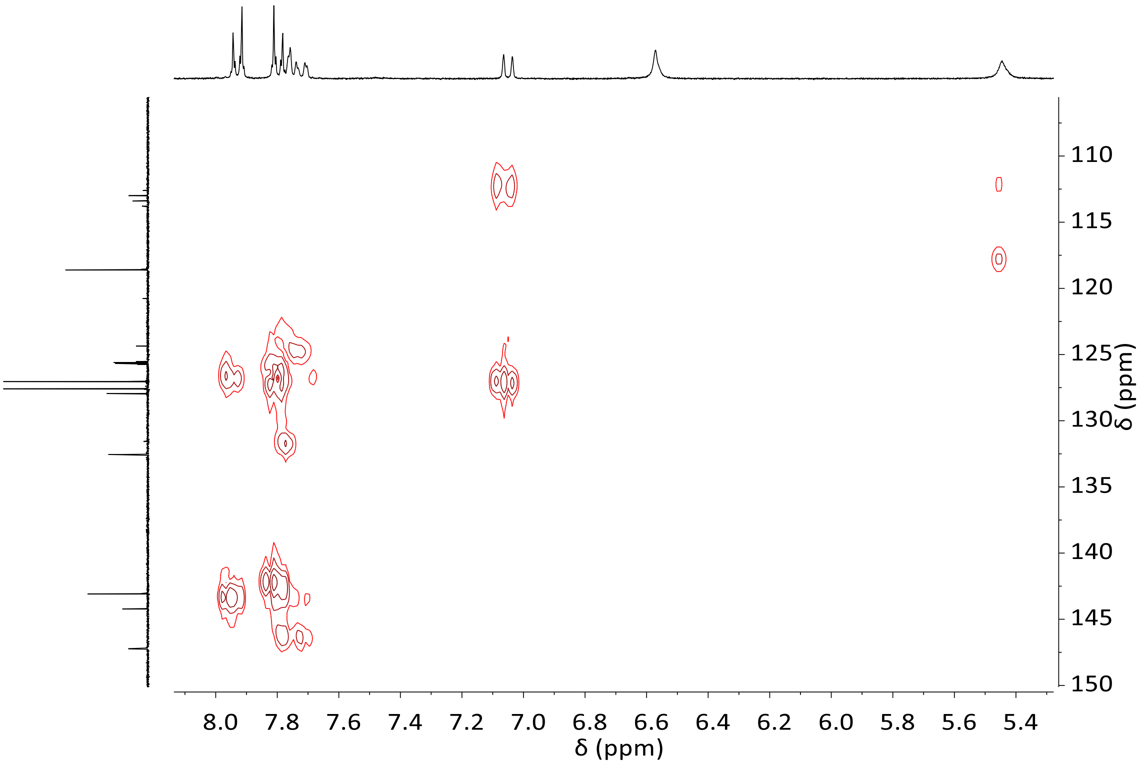

HMBC (300 MHz, 75 MHz, acetone-d_6)

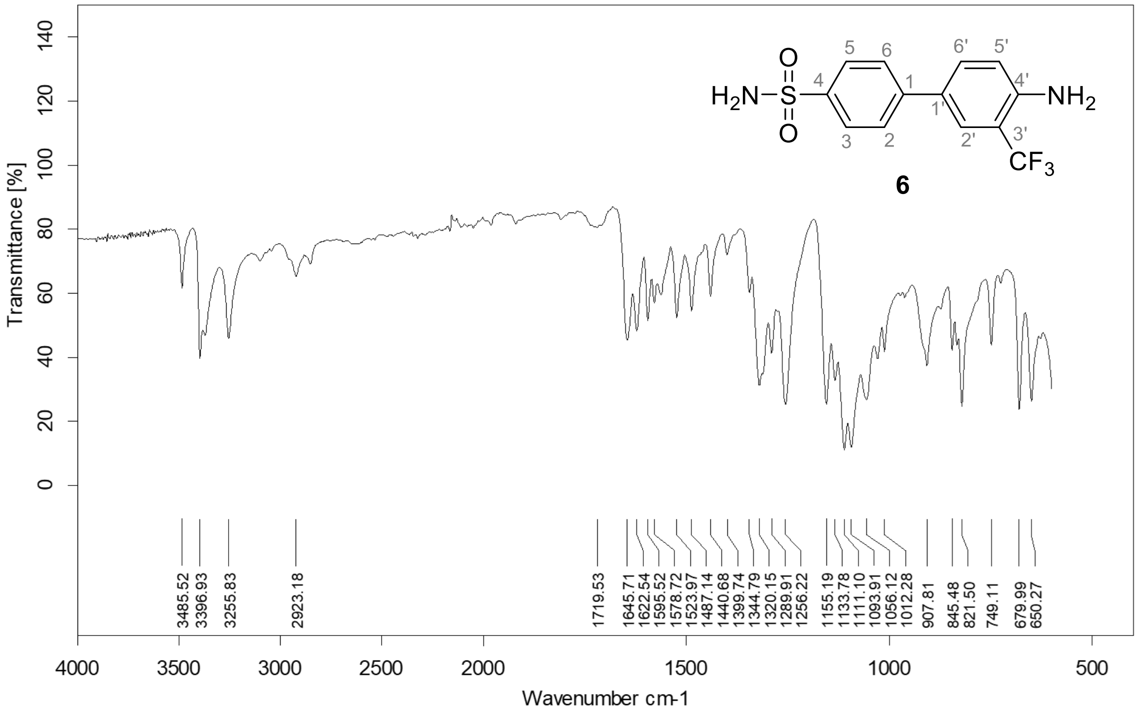

IR (ATR, cm^{-1})

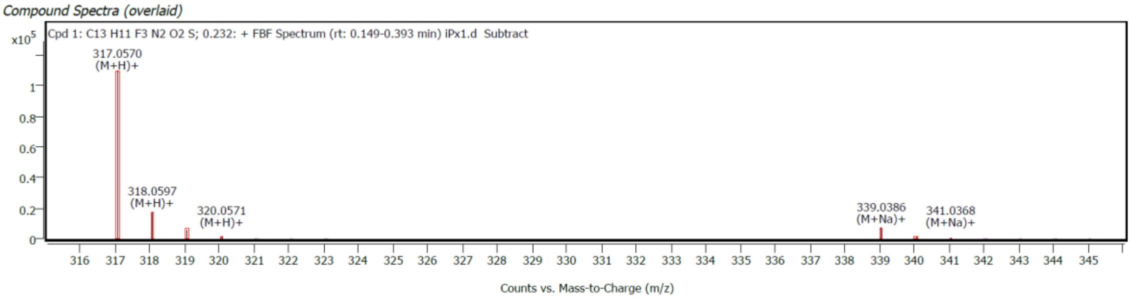

Spectrum Peaks Textbox28

m/z	m/z (Calc)	Diff (ppm)	Abund	Height %	Height % (Calc)	Z	Ion Species	Formula
317.0570	317.0566	1.25	110273	100.00	100.00	1	(M+H)+	C13H11F3N2O2S
318.0597	318.0595	0.70	17066	15.48	15.79	1	(M+H)+	C13H11F3N2O2S
319.0554	319.0549	1.72	5533	5.02	6.05	1	(M+H)+	C13H11F3N2O2S
320.0571	320.0568	0.81	841	0.76	0.79	1	(M+H)+	C13H11F3N2O2S
339.0386	339.0386	0.20	7083	100.00	100.00	1	(M+Na)+	C13H11F3N2O2S
340.0418	340.0414	0.98	1143	16.14	15.78	1	(M+Na)+	C13H11F3N2O2S
341.0368	341.0368	-0.22	418	5.90	6.05	1	(M+Na)+	C13H11F3N2O2S

HRMS (ESI(+))

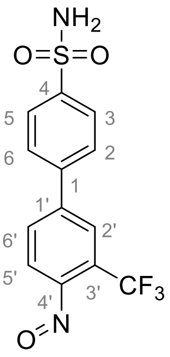

7

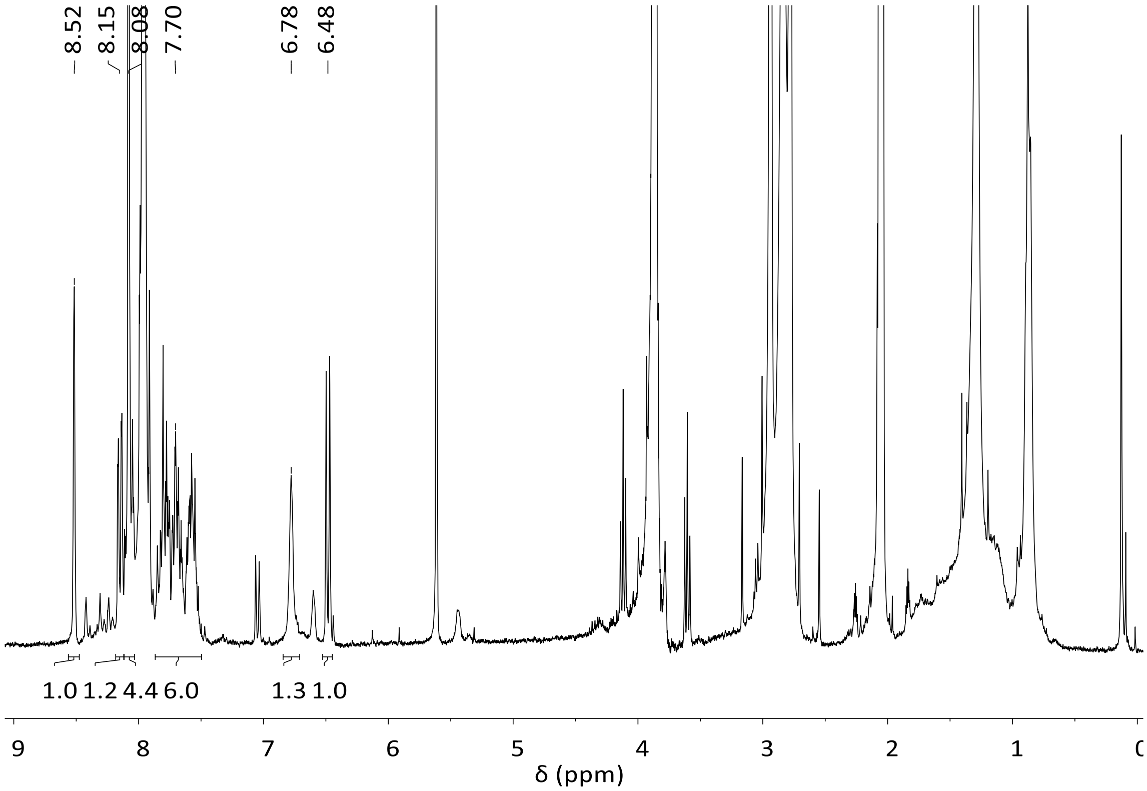

^1H NMR (300 MHz, acetone-d_6)

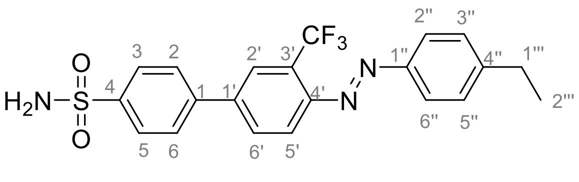

trans-**PC5**

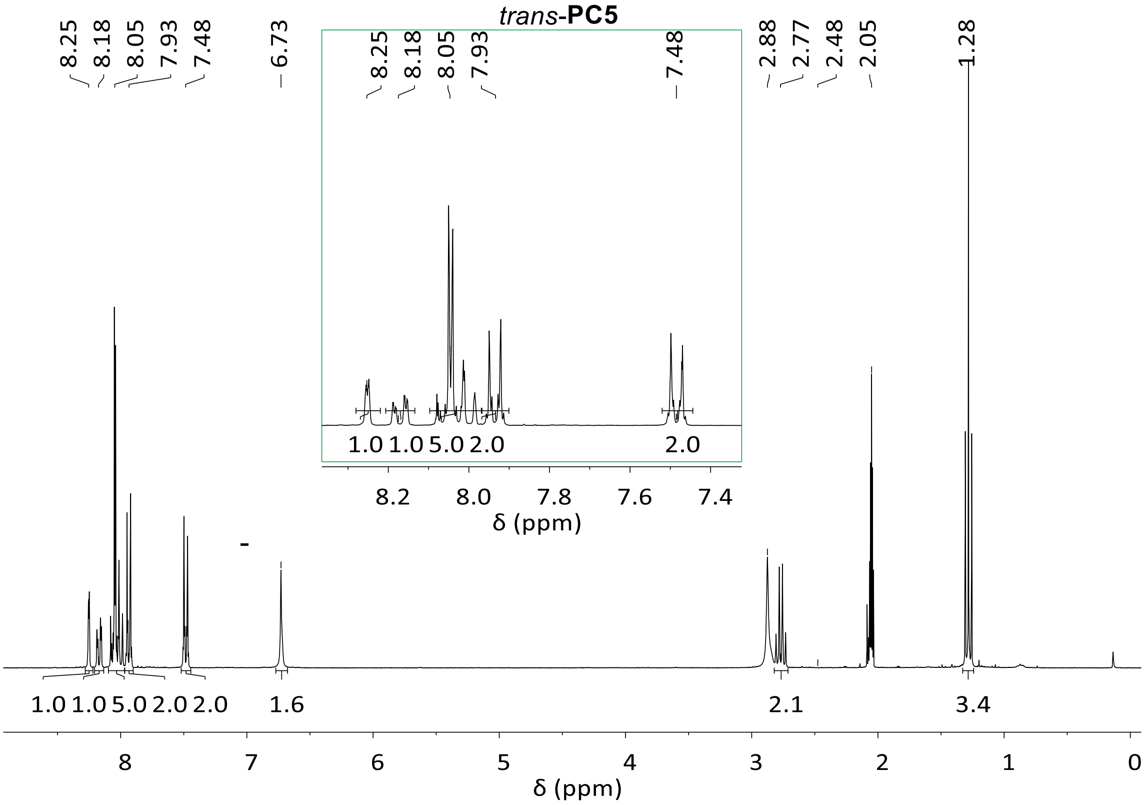

^{1}H NMR (300 MHz, acetone-d_6)

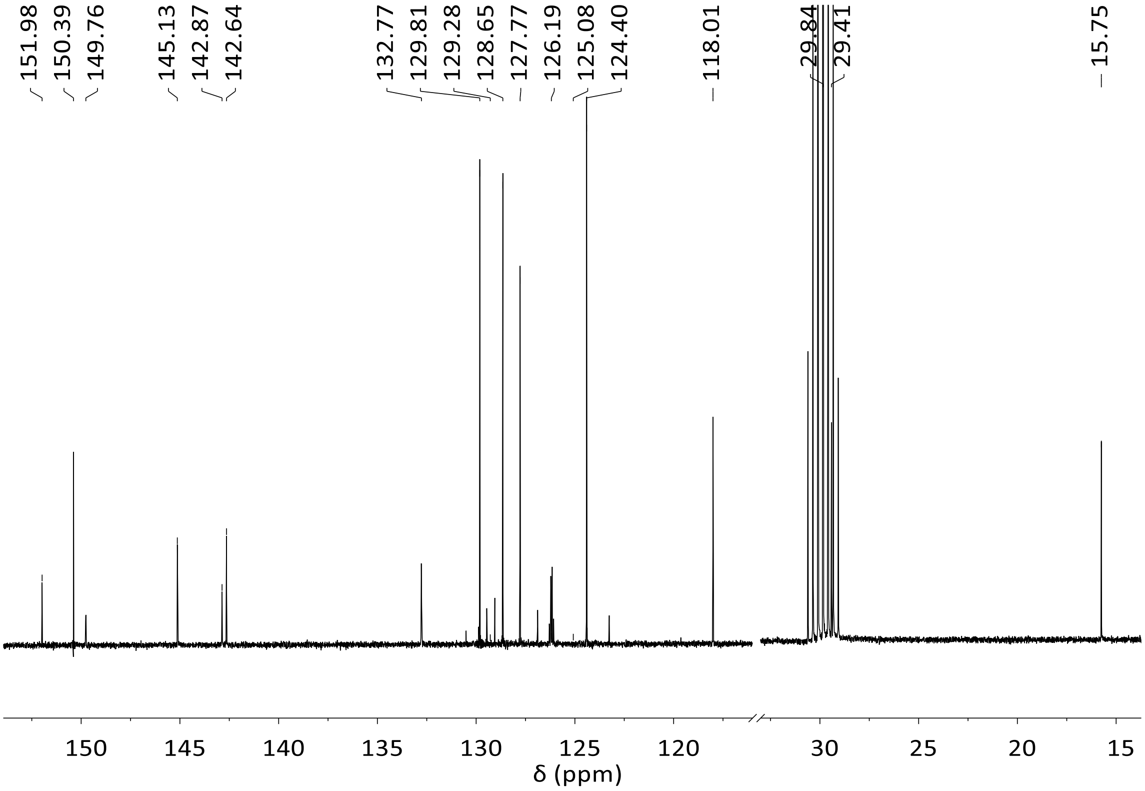

^{13}C NMR (75 MHz, acetone-d_6)

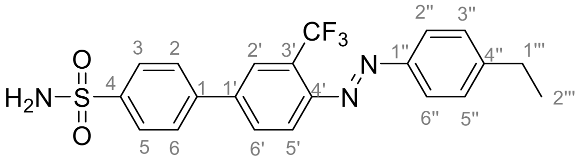

trans-**PC5**

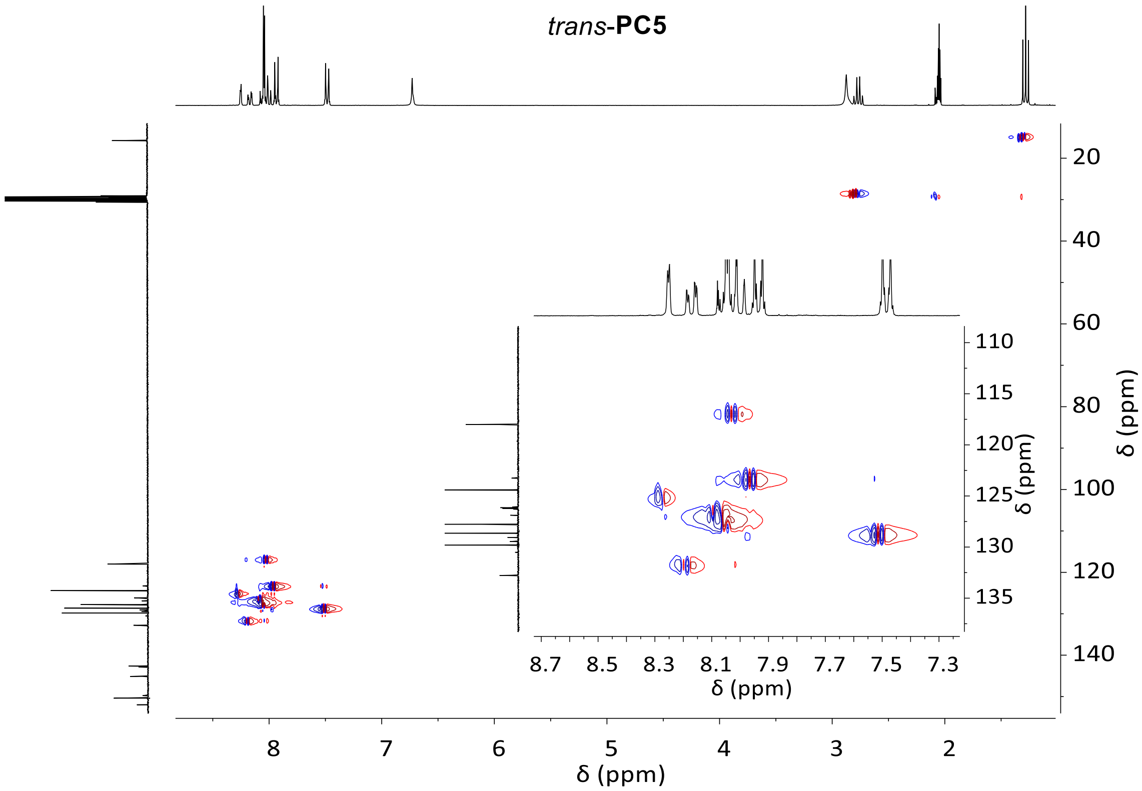

HSQC(ed) (300 MHz, 75 MHz, acetone-d_6)

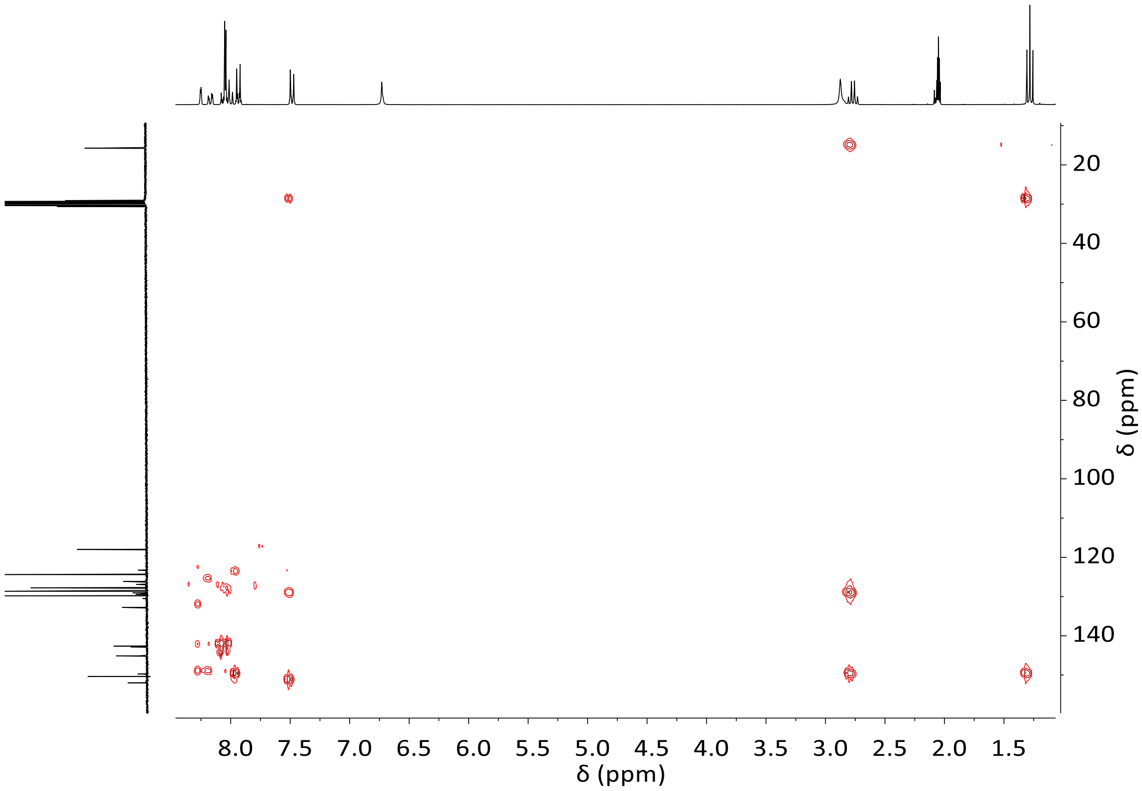

HMBC (300 MHz, 75 MHz, acetone-d_6)

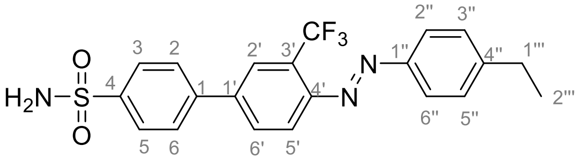

trans-**PC5**

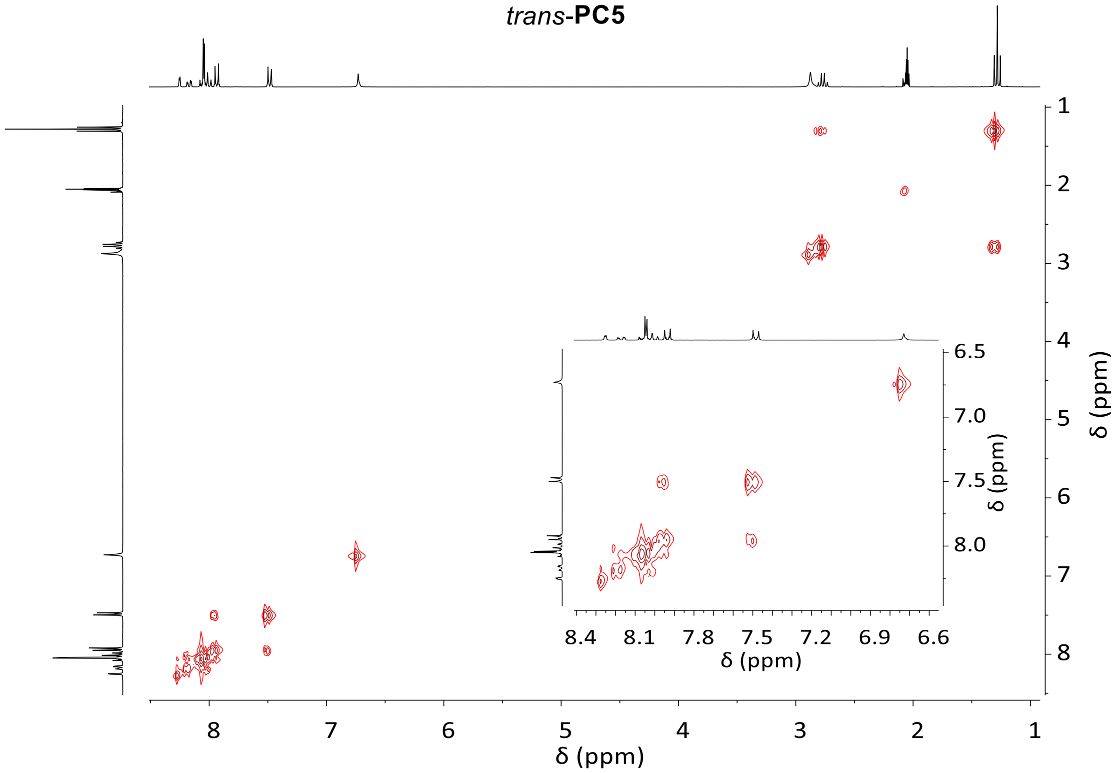

COSY (300 MHz, acetona-d_6)

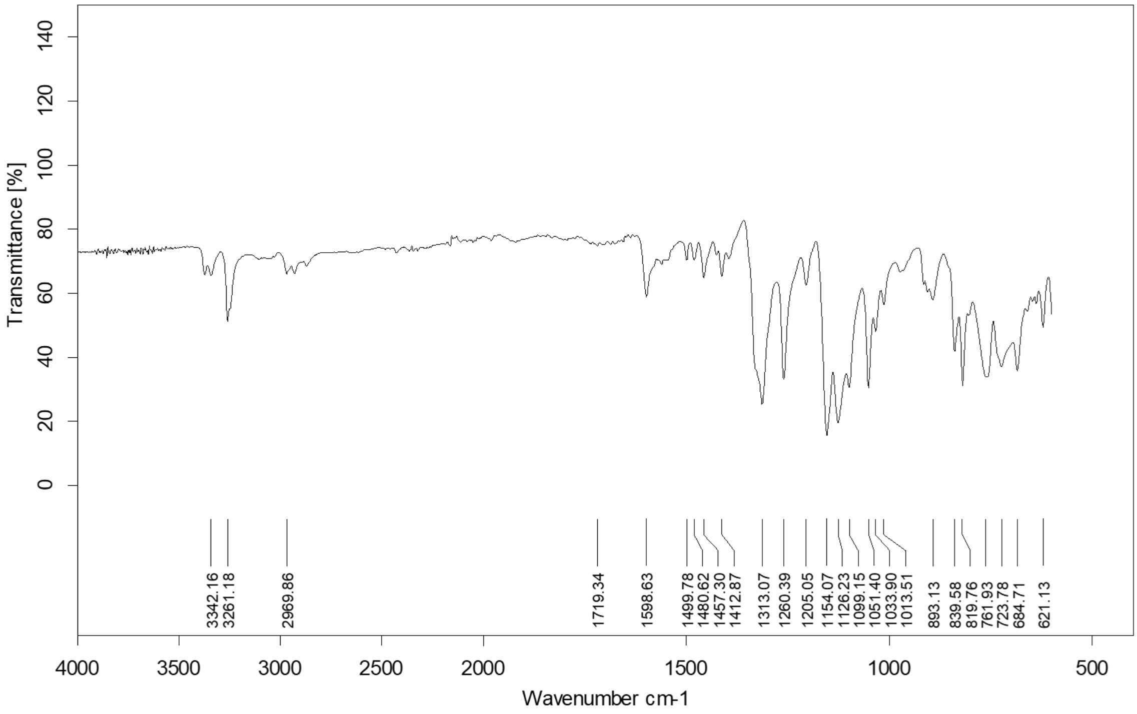

IR (ATR, cm^{-1})

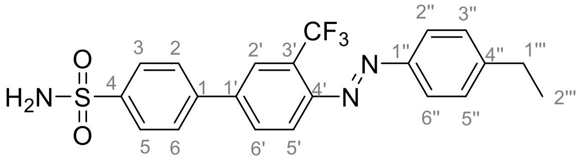

trans-**PC5**

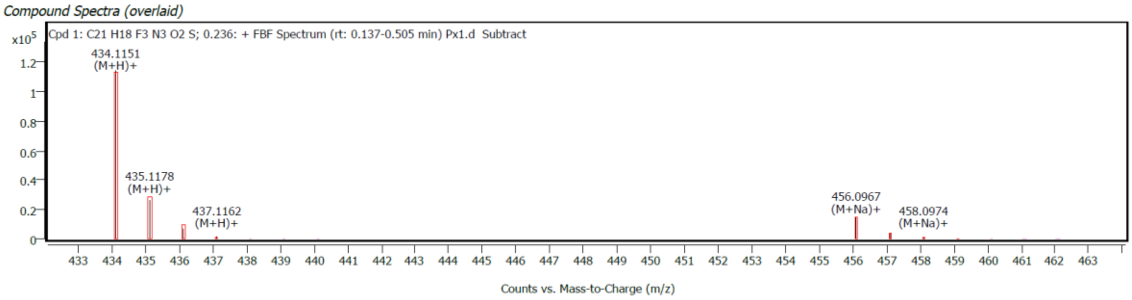

Spectrum Peaks Textbox28

m/z	m/z (Calc)	Diff (ppm)	Abund	Height %	Height % (Calc)	Z	Ion Species	Formula
434.1151	434.1145	1.46	114455	100.00	100.00	1	(M+H)+	C21H18F3N3O2S
435.1178	435.1174	0.86	26205	22.90	24.89	1	(M+H)+	C21H18F3N3O2S
436.1156	436.1145	2.47	7056	6.17	7.86	1	(M+H)+	C21H18F3N3O2S
437.1162	437.1156	1.41	1485	1.30	1.41	1	(M+H)+	C21H18F3N3O2S
456.0967	456.0964	0.65	14717	100.00	100.00	1	(M+Na)+	C21H18F3N3O2S
457.0997	457.0994	0.77	3624	24.62	24.88	1	(M+Na)+	C21H18F3N3O2S
458.0974	458.0965	1.96	1005	6.83	7.85	1	(M+Na)+	C21H18F3N3O2S
459.0969	459.0975	-1.32	125	0.85	1.41	1	(M+Na)+	C21H18F3N3O2S

HRMS (ESI(+))

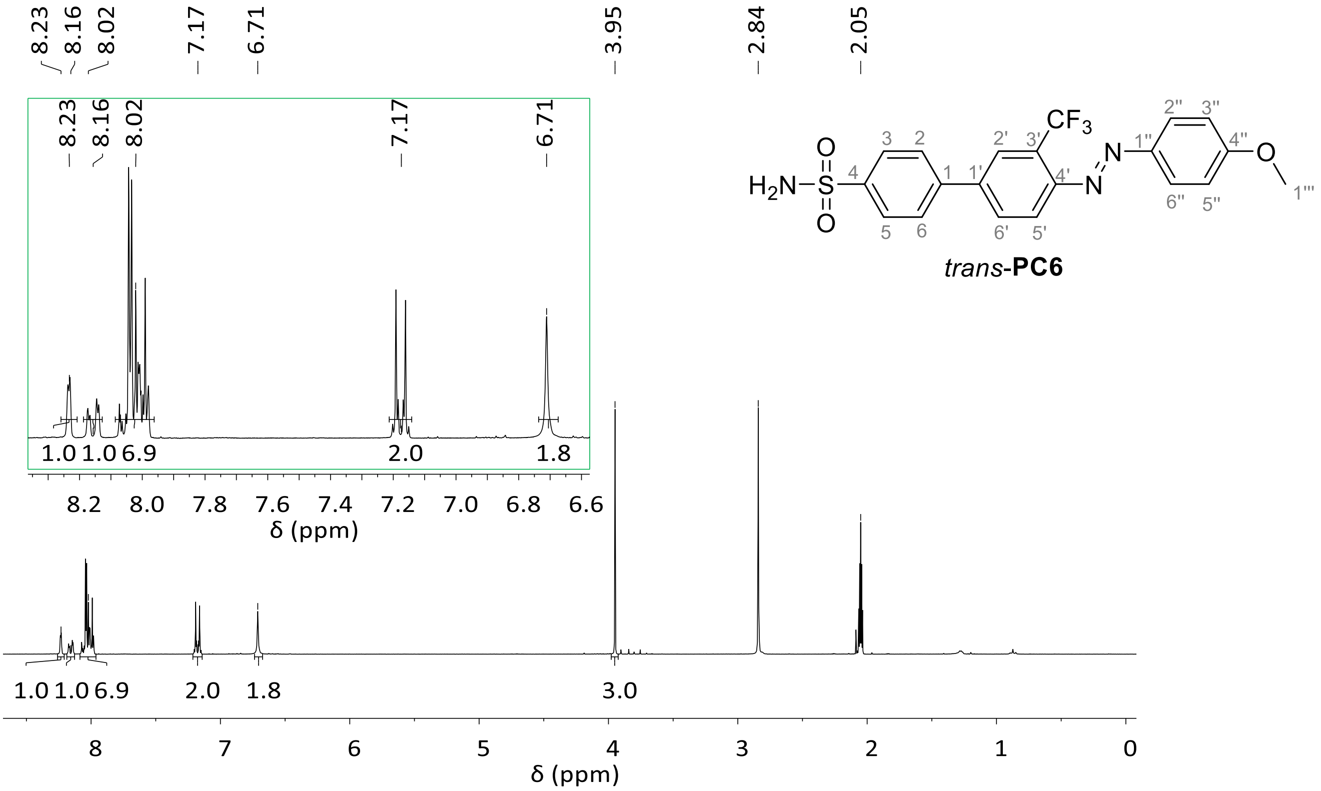

^{1}H NMR (300 MHz, acetone-d_6)

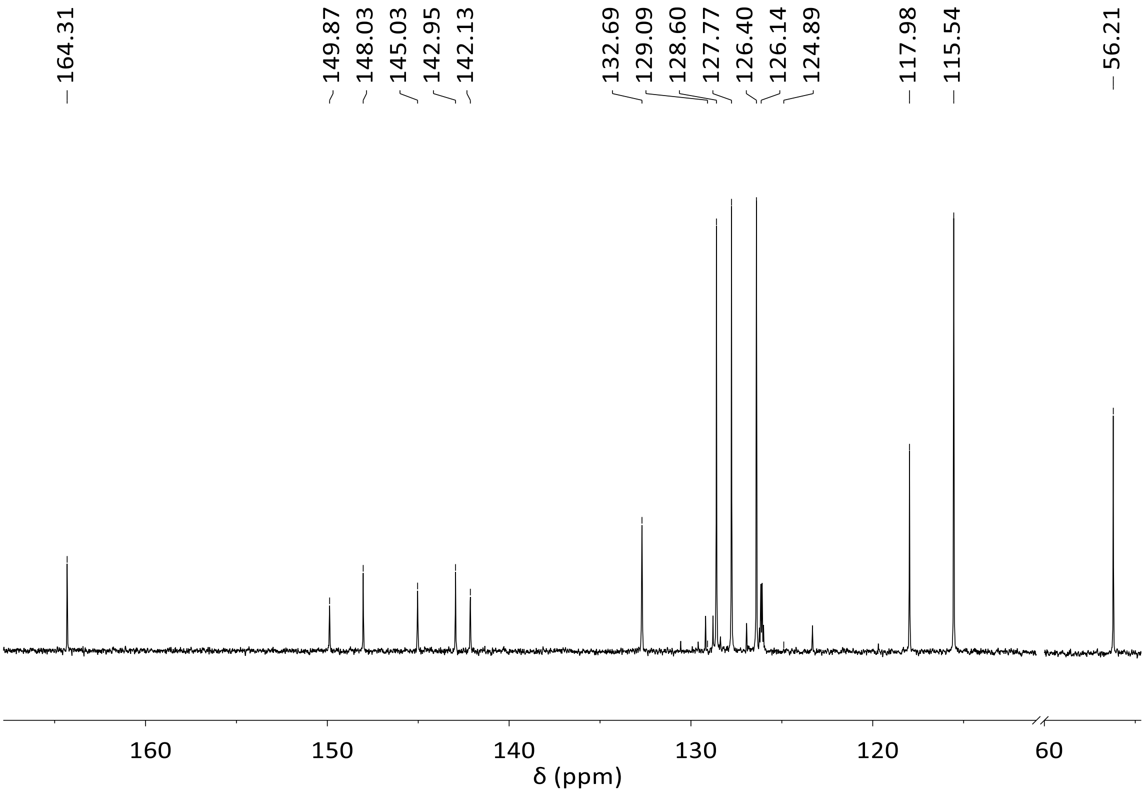

^{13}C NMR (75 MHz, acetone-d_6)

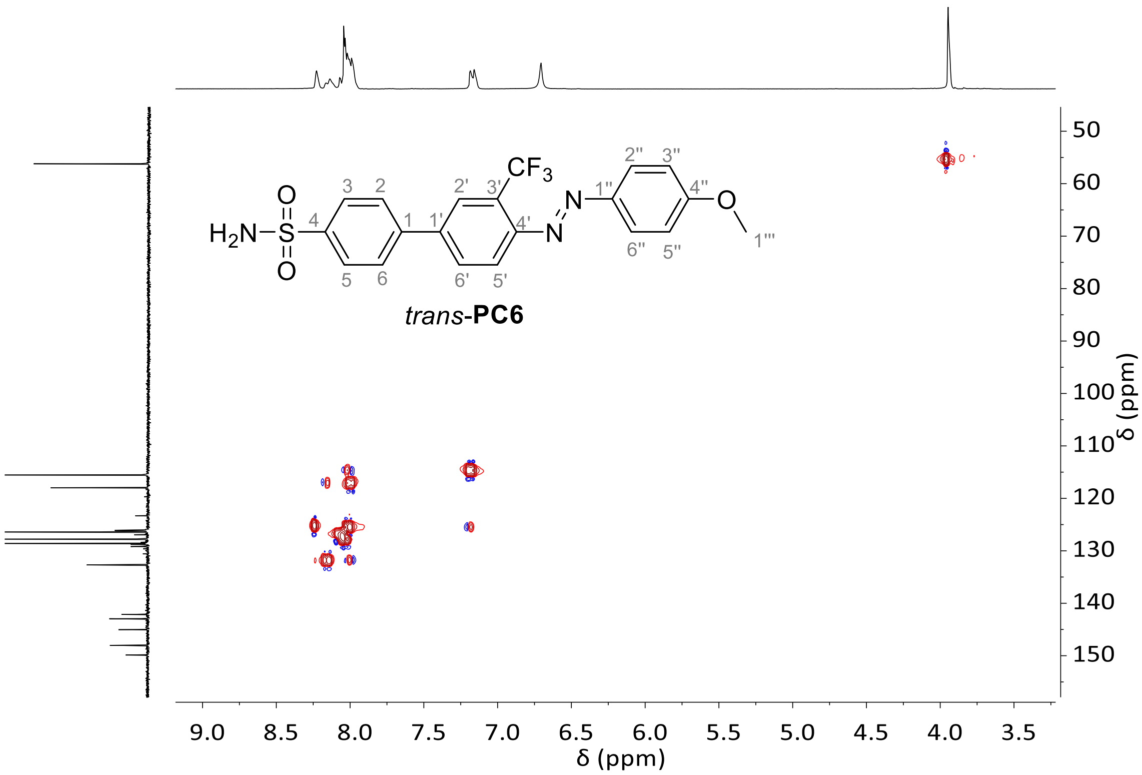

HSQC(ed) (300 MHz, 75 MHz, acetone-*d*6)

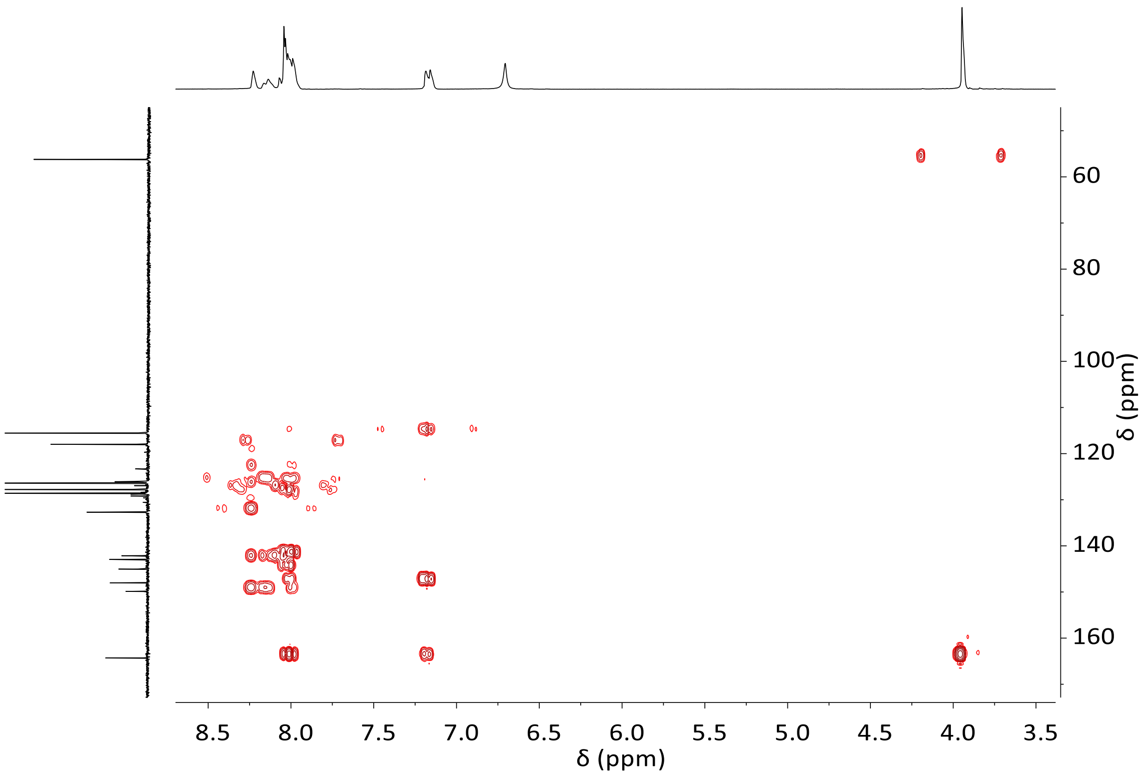

HMBC (300 MHz, 75 MHz, acetone-*d*6)

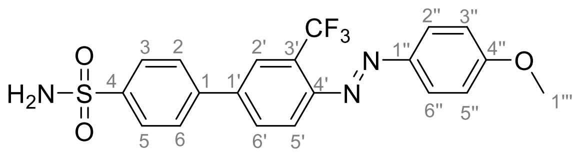

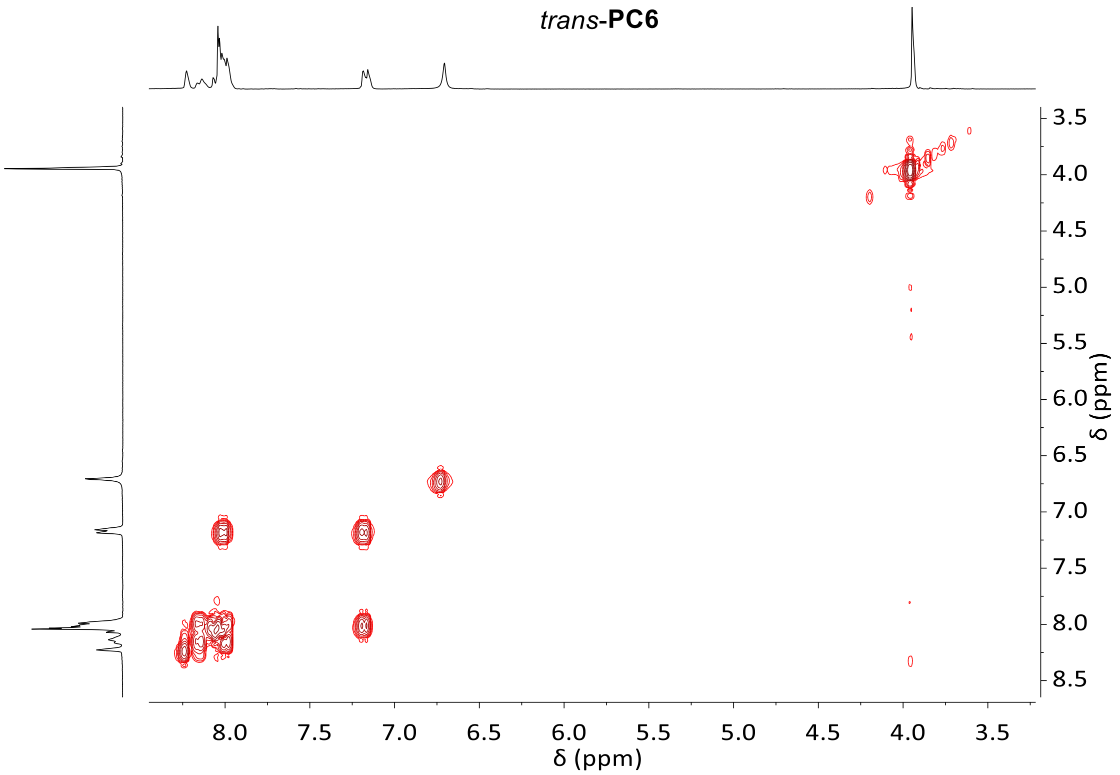

COSY (300 MHz, acetone-d_6)

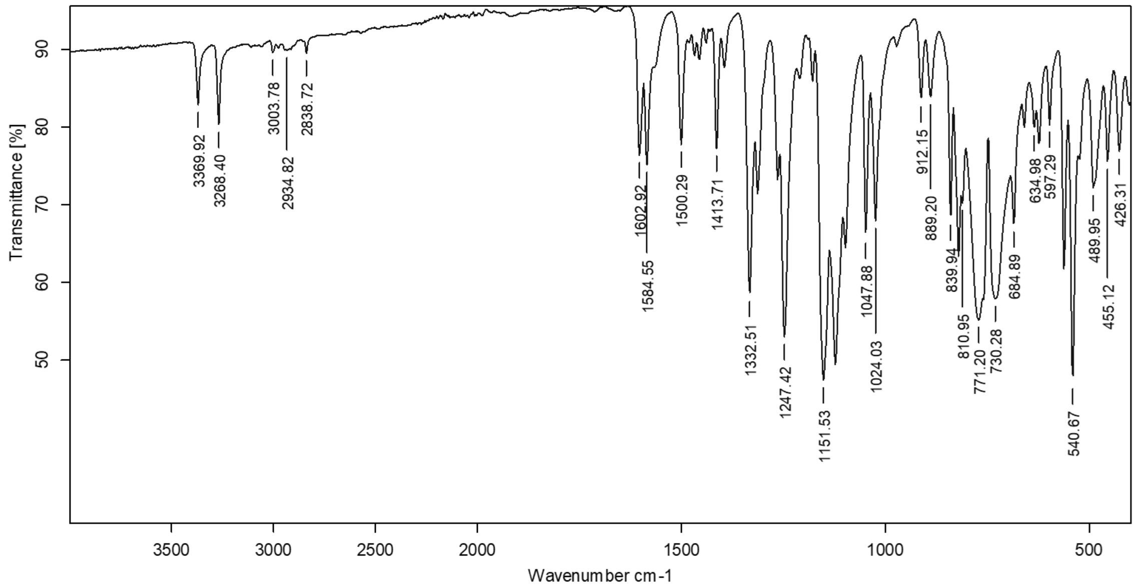

IR (ATR, cm^{-1})

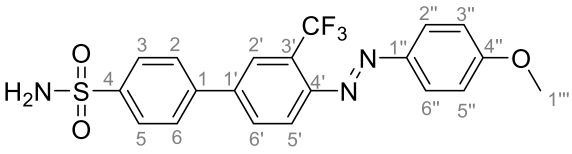

trans-**PC6**

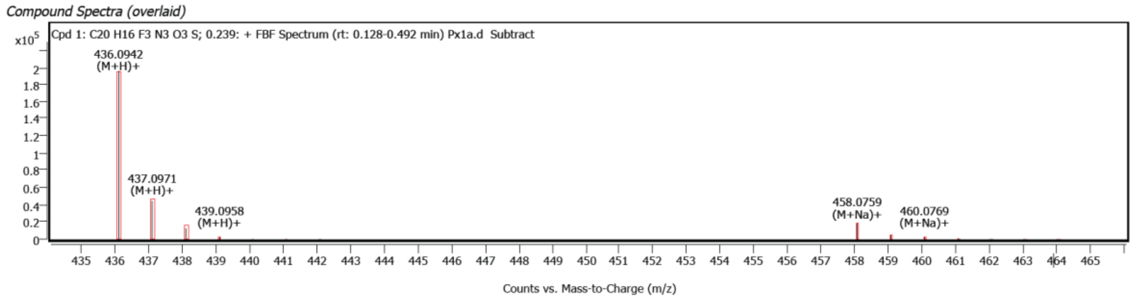

Spectrum Peaks Textbox28

m/z	m/z (Calc)	Diff (ppm)	Abund	Height %	Height % (Calc)	Z	Ion Species	Formula
436.0942	436.0937	1.14	195385	100.00	100.00	1	(M+H)+	C20H16F3N3O3S
437.0971	437.0967	0.89	44025	22.53	23.83	1	(M+H)+	C20H16F3N3O3S
438.0947	438.0937	2.38	11675	5.98	7.81	1	(M+H)+	C20H16F3N3O3S
439.0958	439.0949	1.94	2243	1.15	1.37	1	(M+H)+	C20H16F3N3O3S
458.0759	458.0757	0.42	18625	100.00	100.00	1	(M+Na)+	C20H16F3N3O3S
459.0790	459.0786	0.76	4479	24.05	23.82	1	(M+Na)+	C20H16F3N3O3S
460.0769	460.0756	2.77	1272	6.83	7.80	1	(M+Na)+	C20H16F3N3O3S
461.0771	461.0769	0.57	242	1.30	1.37	1	(M+Na)+	C20H16F3N3O3S

HRMS (ESI(+))

7. References

[1] Lucido M.J., Orlando B.J., Vecchio A.J., Malkowski M.G.. Crystal Structure of Aspirin-Acetylated Human Cyclooxygenase-2: Insight into the Formation of Products with Reversed Stereochemistry. *Biochemistry* **2016**, *55*, 1226-1238. https://doi.org/10.1021/acs.biochem.5b01378.

[2] H++ (web-based computational prediction of protonation states and pK of ionizable groups in macromolecules). Cs.vt.edu. http://newbiophysics.cs.vt.edu/H++/hppdetails.php (accessed 2025-12-04).

[3] Anandakrishnan, R.; Aguilar, B.; Onufriev, A. V. H++ 3.0: Automating pK Prediction and the Preparation of Biomolecular Structures for Atomistic Molecular Modeling and Simulations. *Nucleic Acids Res.* **2012**, *40*, W537-41. https://doi.org/10.1093/nar/gks375.

[4] Myers, J.; Grothaus, G.; Narayanan, S.; Onufriev, A. A Simple Clustering Algorithm Can Be Accurate Enough for Use in Calculations of pKs in Macromolecules. *Proteins* **2006**, *63*, 928–938. https://doi.org/10.1002/prot.20922.

[5] Gordon, J. C.; Myers, J. B.; Folta, T.; Shoja, V.; Heath, L. S.; Onufriev, A. H++: A Server for Estimating pKas and Adding Missing Hydrogens to Macromolecules. *Nucleic Acids Res.* **2005**, *33*, W368-71. https://doi.org/10.1093/nar/gki464.

[6] Jones, G.; Willett, P.; Glen, R. C.; Leach, A. R.; Taylor, R. Development and Validation of a Genetic Algorithm for Flexible Docking. *J. Mol. Biol.* **1997**, *267*, 727–748. https://doi.org/10.1006/jmbi.1996.0897.

[7] D.A. Case, H.M. Aktulga, K. Belfon, I.Y. Ben-Shalom, J.T. Berryman, S.R. Brozell, F.S. Carvahol, D.S. Cerutti, T.E. Cheatham, III, G.A. Cisneros, V.W.D. Cruzeiro, T.A. Darden, N. Forouzesh, M. Ghazimirsaeed, G. Giambaşu, T. Giese, M.K. Gilson, H. Gohlke, A.W. Goetz, J. Harris, Z. Huang, S. Izadi, S.A. Izmailov, K. Kasavajhala, M.C. Kaymak, I. Kolossv\'a ry, A. Kovalenko, T. Kurtzman, T.S. Lee, P. Li, Z. Li, C. Lin, J. Liu, T. Luchko, R. Luo, M. Machado, M. Manathunga, K.M. Merz, Y. Miao, O. Mikhailovskii, G. Monard, H. Nguyen, K.A. O'Hearn, A. Onufriev, F. Pan, S. Pantano, A. Rahnamoun, D.R. Roe, A. Roitberg, C. Sagui, S. Schott-Verdugo, A. Shajan, J. Shen, C.L. Simmerling, N.R. Skrynnikov, J. Smith, J. Swails, R.C. Walker, J. Wang, J. Wang, X. Wu, Y. Wu, Y. Xiong, Y. Xue, D.M. York, C. Zhao, Q. Zhu, and P.A. Kollman (2025), Amber 2025, University of California, San Francisco.

[8] Case, D. A.; Aktulga, H. M.; Belfon, K.; Cerutti, D. S.; Cisneros, G. A.; Cruzeiro, V. W. D.; Forouzesh, N.; Giese, T. J.; Götz, A. W.; Gohlke, H.; Izadi, S.; Kasavajhala, K.; Kaymak, M. C.; King, E.; Kurtzman, T.; Lee, T.-S.; Li, P.; Liu, J.; Luchko, T.; Luo, R.; Manathunga, M.; Machado, M. R.; Nguyen, H. M.; O'Hearn, K. A.; Onufriev, A. V.; Pan, F.; Pantano, S.; Qi, R.; Rahnamoun, A.; Risheh, A.; Schott-Verdugo, S.; Shajan, A.; Swails, J.; Wang, J.; Wei, H.; Wu, X.; Wu, Y.;

Zhang, S.; Zhao, S.; Zhu, Q.; Cheatham, T. E., 3rd; Roe, D. R.; Roitberg, A.; Simmerling, C.; York, D. M.; Nagan, M. C.; Merz, K. M., Jr. AmberTools. *J. Chem. Inf. Model.* **2023**, *63*, 6183–6191. https://doi.org/10.1021/acs.jcim.3c01153.

[9] Jones, G.; Willett, P.; Glen, R. C.; Leach, A. R.; Taylor, R. Development and Validation of a Genetic Algorithm for Flexible Docking. *J. Mol. Biol.* **1997**, *267*, 727–748. https://doi.org/10.1006/jmbi.1996.0897.

[10] Gaussian 16, Revision C.01, M. J. Frisch, G. W. Trucks, H. B. Schlegel, G. E. Scuseria, M. A. Robb, J. R. Cheeseman, G. Scalmani, V. Barone, G. A. Petersson, H. Nakatsuji, X. Li, M. Caricato, A. V. Marenich, J. Bloino, B. G. Janesko, R. Gomperts, B. Mennucci, H. P. Hratchian, J. V. Ortiz, A. F. Izmaylov, J. L. Sonnenberg, D. Williams-Young, F. Ding, F. Lipparini, F. Egidi, J. Goings, B. Peng, A. Petrone, T. Henderson, D. Ranasinghe, V. G. Zakrzewski, J. Gao, N. Rega, G. Zheng, W. Liang, M. Hada, M. Ehara, K. Toyota, R. Fukuda, J. Hasegawa, M. Ishida, T. Nakajima, Y. Honda, O. Kitao, H. Nakai, T. Vreven, K. Throssell, J. A. Montgomery, Jr., J. E. Peralta, F. Ogliaro, M. J. Bearpark, J. J. Heyd, E. N. Brothers, K. N. Kudin, V. N. Staroverov, T. A. Keith, R. Kobayashi, J. Normand, K. Raghavachari, A. P. Rendell, J. C. Burant, S. S. Iyengar, J. Tomasi, M. Cossi, J. M. Millam, M. Klene, C. Adamo, R. Cammi, J. W. Ochterski, R. L. Martin, K. Morokuma, O. Farkas, J. B. Foresman, and D. J. Fox, Gaussian, Inc., Wallingford CT, 2016.

[11] Duchstein, P.; Neiss, C.; Görling, A.; Zahn, D. Molecular Mechanics Modeling of Azobenzene-Based Photoswitches. *J. Mol. Model.* **2012**, *18*, 2479–2482. https://doi.org/10.1007/s00894-011-1270-6.

[12] Duan, L.; Liu, X.; Zhang, J. Z. H. Interaction Entropy: A New Paradigm for Highly Efficient and Reliable Computation of Protein-Ligand Binding Free Energy. *J. Am. Chem. Soc.* **2016**, *138*, 5722–5728. https://doi.org/10.1021/jacs.6b02682.

[13] Cruz Saez, A.; Pérez-Sánchez, Á. *Setup and calculation of entropy for MM-PBSA binding energies*; Zenodo, 2023.

[14] Lees, A. J. A Photochemical Procedure for Determining Reaction Quantum Efficiencies in Systems with Multicomponent Inner Filter Absorbances. *Anal. Chem.* **1996**, *68*, 226–229. https://doi.org/10.1021/ac9507653.

[15] Irie, M.; Lifka, T.; Kobatake, S.; Kato, N. Photochromism of 1,2-Bis(2-Methyl-5-Phenyl-3-Thienyl)Perfluorocyclopentene in a Single-Crystalline Phase. *J. Am. Chem. Soc.* **2000**, *122*, 4871–4876. https://doi.org/10.1021/ja993181h

[16] D'Avino, D.; Cerqua, I.; Ullah, H.; Spinelli, M.; Di Matteo, R.; Granato, E.; Capasso, R.; Maruccio, L.; Ialenti, A.; Daglia, M.; Roviezzo, F.; Rossi, A. Beneficial Effects of *Astragalus membranaceus* (Fisch.) Bunge Extract in Controlling Inflammatory Response and Preventing Asthma Features. *Int. J. Mol. Sci.* **2023**, *24*, 10954. https://doi.org/10.3390/ijms241310954.

[17] Cerqua, I.; Musella, S.; Peltner, L. K.; D'Avino, D.; Di Sarno, V.; Granato, E.; Vestuto, V.; Di Matteo, R.; Pace, S.; Ciaglia, T.; Bilancia, R.; Smaldone, G.; Di Matteo, F.; Di Micco, S.; Bifulco, G.; Pepe, G.; Basilicata, M. G.; Rodriquez, M.; Gomez-Monterrey, I. M.; Campiglia, P.; Ostacolo, C.; Roviezzo, F.; Werz, O.; Rossi, A.; Bertamino, A. Discovery and Optimization of Indoline-Based Compounds as Dual 5-LOX/sEH Inhibitors: In Vitro and In Vivo Anti-Inflammatory Characterization. *J. Med. Chem.* **2022**, *65*, 14456–14480. https://doi.org/10.1021/acs.jmedchem.2c00817.

[18] Kimmel, C. B.; Ballard, W. W.; Kimmel, S. R.; Ullmann, B.; Schilling, T. F. Stages of Embryonic Development of the Zebrafish. *Dev. Dyn.* **1995**, *203*, 253–310. https://doi.org/10.1002/aja.1002030302.

[19] Renshaw, S. A.; Loynes, C. A.; Trushell, D. M. I.; Elworthy, S.; Ingham, P. W.; Whyte, M. K. B. A Transgenic Zebrafish Model of Neutrophilic Inflammation. *Blood* **2006**, *108*, 3976–3978. https://doi.org/10.1182/blood-2006-05-024075.

[20] Isles, H. M.; Herman, K. D.; Robertson, A. L.; et al. The CXCL12/CXCR4 Signaling Axis Retains Neutrophils at Inflammatory Sites in Zebrafish. *Front. Immunol.* **2019**, *10*, 1784. https://doi.org/10.3389/fimmu.2019.01784.

[21] Kim, J.; Choi, J.; Lee, J.; Park, S.; Kim, H. S.; Jung, D.; Koh, J.S.; Kim, S.; Lee, J.; Song, H. Preparation of pyrrolo/pyrazolopyrimidine derivatives as LRRK2 inhibitors. Patent WO2019112269A1, June 13, **2013**.

[22] Maspero, A.; Giovenzana, G. B.; Monticelli, D.; Tagliapietra, S.; Palmisano, G.; Penoni, A. Filling the Gap: Chemistry of 3,5-bis(trifluoromethyl)-1H-pyrazoles. *J. Fluorine Chem.* **2012**, *139*, 53-57.

[23] Gross, R. S.; Guo, Z.; Dyck, B.; Coon, T.; Huang, C. Q.; Lowe, R. F.; Marinkovic, D.; Moorjani, M.; Nelson, J.; Zamani-Kord, S.; Grigoriadis, D. E.; Hoare, S. R. J.; Crowe, P. D.; Han Bu, J.; Haddach, M.; McCarthy, J.; Saunders, J.; Sullivan, R.; Chen, T. K.; Williams, J. P. Design and Synthesis of Tricyclic Corticotropin-Releasing Factor-1 Antagonists. *J. Med. Chem.* **2005**, *48*, 5780–5793. https://doi.org/10.1021/jm049085v

[24] Xu, P.; Shen, P.; Wang, H.; Qin, L.; Ren, J.; Sun, Q.; Ge, R.; Bian, J.; Zhong, Y..; Li, Z.; Wang, J.; Qiu, Z. Discovery of imidazopyrrolopyridines derivatives as novel and selective inhibitors of JAK2. *Eur. J. Med. Chem.* **2021**, *218*, 113394. https://doi.org/10.1016/j.ejmech.2021.113394.

[25] Xiao-Yu, H.; Jia, K.; Cao, Y.; Li, Y.; Qin, S.; Zhou, F.; Lin, C.; Zhang, D.; Wang, L. Dual Photo- and pH-Responsive Supramolecular Nanocarriers Based on Water-Soluble Pillar[6]arene and Different Azobenzene Derivatives for Intracellular Anticancer Drug Delivery. *Chem. Eur. J.* **2015**, *21*, 1208-1220. https://doi.org/10.1002/chem.201405095.
